# Supplementary material for: Genome-Wide Analysis of Alternative Splicing and Non-Coding RNAs Reveal Complicated Transcriptional Regulation in Cannabis sativa L
Source: Int J Mol Sci. 2021 Nov 5;22(21):11989. doi: 10.3390/ijms222111989 (PMC8584933; doi:10.3390/ijms222111989)
Supplement: Supplementary file 1 [file ijms-22-11989-s001.zip › Table S13. The cleavage site of miRNA targets was predicted by psRobot software..pdf]

```
>miR1508-3P
    Score: 2.5  Deg: 16:2290:44:6096   T_00052443

Query:          1 CTAGAAAGAGAGAGAGCCGT-T
 22
                  *|||||||||||:||||::*|*
Sbjct:       2299 AATCTTTCTCTCTTTCGGTGCAC 2277
                  || |   | |||| ||| |||||-------------------------------7
                  || |   | |||| ||| ||||--------------------------------11
                  || |   | |||| ||| |||---------------------------------3
                  || |   | |||| ||| ||----------------------------------3
                  || |   | |||| ||| |-----------------------------------3
                  || |   | |||| |||-------------------------------------2
                  || |   | |||| ||--------------------------------------3
                  || |   | |||| |---------------------------------------2
                  || |   | ||||-----------------------------------------1
                  || |   | |||------------------------------------------2
                  || |   | ||-------------------------------------------1
                  || |   | |--------------------------------------------16
                  || |   |----------------------------------------------1
                  || |--------------------------------------------------2
                  ||----------------------------------------------------1
                  |-----------------------------------------------------4

>miR1508-3P
    Score: 2.5  Deg: 7:788:7:222   T_00040034

Query:          1 CTAGAAAGAGAGAGAGCCGTT
 22
                  *||||||||||||||||*|***
Sbjct:        797 CATCTTTCTCTCTCTCGACTTT 776
                  ||||||||||||   | | | |-------------------------------2
                  ||||||||||||   | | |---------------------------------1
                  ||||||||||||   | |-----------------------------------2
                  ||||||||||||   |-------------------------------------1
                  ||||||||||||-----------------------------------------1
                  |||||||||||------------------------------------------1
                  ||||||||||-------------------------------------------7
                  |||||||||--------------------------------------------5
                  ||||||||---------------------------------------------3
                  |||||||----------------------------------------------1
                  ||||||-----------------------------------------------1
                  |||||------------------------------------------------1
                  ||||-------------------------------------------------2
                  |||--------------------------------------------------1
```

```
                    ||--------------------------------------------------2
                    |---------------------------------------------------3


>miR156a
    Score: 1.5  Deg: 10:1311:27:223T_00091783

Query:          1 TGACAGAAGAGAGTGAGCAC
 21
                  ||||||||||||*||||||*
Sbjct:       1319 ACTGTCTTCTCTCTCTCGTGT 1299
                         ||||    |    |------------------------------1
                         ||||    |-----------------------------------2
                         ||||----------------------------------------6
                         |||-----------------------------------------6
                         ||------------------------------------------10
                         |-------------------------------------------1

>miR156a
    Score: 1.5  Deg: 10:1668:27:226T_00091779

Query:          1 TGACAGAAGAGAGTGAGCAC
 21
                  ||||||||||||*||||||*
Sbjct:       1676 ACTGTCTTCTCTCTCTCGTGT 1656
                         ||||    |    |------------------------------1
                         ||||    |-----------------------------------2
                         ||||----------------------------------------6
                         |||-----------------------------------------6
                         ||------------------------------------------10
                         |-------------------------------------------1

>miR156a
    Score: 1.5  Deg: 10:2823:27:234T_00091781

Query:          1 TGACAGAAGAGAGTGAGCAC
 21
                  ||||||||||||*||||||*
Sbjct:       2831 ACTGTCTTCTCTCTCTCGTGT 2811
                         ||||    |    |------------------------------1
                         ||||    |-----------------------------------2
                         ||||----------------------------------------6
                         |||-----------------------------------------6
                         ||------------------------------------------10
```

```
                         |-------------------------------------------1

>miR156a
    Score: 1.5  Deg: 10:1470:27:223T_00091782

Query:          1 TGACAGAAGAGAGTGAGCAC
 21
                  ||||||||||||*||||||*
Sbjct:       1478 ACTGTCTTCTCTCTCTCGTGT 1458
                         ||||    |    |-------------------------------1
                         ||||    |------------------------------------2
                         ||||-----------------------------------------6
                         |||------------------------------------------6
                         ||-------------------------------------------10
                         |-------------------------------------------1

>miR156a
    Score: 1.5  Deg: 10:1199:27:223T_00091784

Query:          1 TGACAGAAGAGAGTGAGCAC
 21
                  ||||||||||||*||||||*
Sbjct:       1207 ACTGTCTTCTCTCTCTCGTGT 1187
                         ||||    |    |-------------------------------1
                         ||||    |------------------------------------2
                         ||||-----------------------------------------6
                         |||------------------------------------------6
                         ||-------------------------------------------10
                         |-------------------------------------------1

>miR156a
    Score: 1.5  Deg: 10:1496:27:226T_00091780

Query:          1 TGACAGAAGAGAGTGAGCAC
 21
                  ||||||||||||*||||||*
Sbjct:       1504 ACTGTCTTCTCTCTCTCGTGT 1484
                         ||||    |    |-------------------------------1
                         ||||    |------------------------------------2
                         ||||-----------------------------------------6
                         |||------------------------------------------6
                         ||-------------------------------------------10
                         |-------------------------------------------1

>miR156a
```

```
    Score: 2.5  Deg: 22:1017:24:363T_00090332

Query:          1 TGACAGAAGAGAGTGAGCAC
 21
                  ||||||||||*||*||||||*
Sbjct:       1025 ACTGTCTTCTATCTCTCGTGC 1005
                      | | ||||||  |-----------------------------------2
                      | | ||||||--------------------------------------1
                      | | |||||---------------------------------------1
                      | | ||||----------------------------------------1
                      | | |||-----------------------------------------2
                      | | ||------------------------------------------3
                      | | |-------------------------------------------22
                      | |---------------------------------------------1
                      |-----------------------------------------------2

>miR156a
    Score: 2.5  Deg: 22:1088:24:371T_00090336

Query:          1 TGACAGAAGAGAGTGAGCAC
 21
                  ||||||||||*||*||||||*
Sbjct:       1096 ACTGTCTTCTATCTCTCGTGC 1076
                      | | ||||||  |-----------------------------------2
                      | | ||||||--------------------------------------1
                      | | |||||---------------------------------------1
                      | | ||||----------------------------------------1
                      | | |||-----------------------------------------2
                      | | ||------------------------------------------3
                      | | |-------------------------------------------22
                      | |---------------------------------------------1
                      |-----------------------------------------------2

>miR156a
    Score: 2.5  Deg: 22:1171:24:378T_00090333

Query:          1 TGACAGAAGAGAGTGAGCAC
 21
                  ||||||||||*||*||||||*
Sbjct:       1179 ACTGTCTTCTATCTCTCGTGC 1159
                      | | ||||||  |-----------------------------------2
                      | | ||||||--------------------------------------1
                      | | |||||---------------------------------------1
                      | | ||||----------------------------------------1
                      | | |||-----------------------------------------2
```

```
                    | | ||----------------------------------------3
                    | | |-----------------------------------------22
                    | |-------------------------------------------1
                    |---------------------------------------------2

>miR156a
    Score: 2.5  Deg: 22:1129:24:363T_00090331

Query:          1 TGACAGAAGAGAGTGAGCAC
 21
                  ||||||||||*||*||||||*
Sbjct:       1137 ACTGTCTTCTATCTCTCGTGC 1117
                    | | ||||||  |---------------------------------2
                    | | ||||||------------------------------------1
                    | | |||||-------------------------------------1
                    | | ||||--------------------------------------1
                    | | |||---------------------------------------2
                    | | ||----------------------------------------3
                    | | |-----------------------------------------22
                    | |-------------------------------------------1
                    |---------------------------------------------2

>miR156a
    Score: 2.5  Deg: 22:958:24:363 T_00090335

Query:          1 TGACAGAAGAGAGTGAGCAC
 21
                  ||||||||||*||*||||||*
Sbjct:        966 ACTGTCTTCTATCTCTCGTGC 946
                    | | ||||||  |---------------------------------2
                    | | ||||||------------------------------------1
                    | | |||||-------------------------------------1
                    | | ||||--------------------------------------1
                    | | |||---------------------------------------2
                    | | ||----------------------------------------3
                    | | |-----------------------------------------22
                    | |-------------------------------------------1
                    |---------------------------------------------2

>miR156a
    Score: 2.5  Deg: 22:941:24:361 T_00090334

Query:          1 TGACAGAAGAGAGTGAGCAC
 21
                  ||||||||||*||*||||||*
```

```
Sbjct:        949 ACTGTCTTCTATCTCTCGTGC 929
                      | | ||||||  |-----------------------------------2
                      | | ||||||---------------------------------------1
                      | | |||||----------------------------------------1
                      | | ||||-----------------------------------------1
                      | | |||------------------------------------------2
                      | | ||-------------------------------------------3
                      | | |--------------------------------------------22
                      | |----------------------------------------------1
                      |------------------------------------------------2

>miR156a
    Score: 2.0  Deg: 79:1218:79:519T_00059648

Query:          1 TGACAGAAGAGAGTGAGCAC
 21
                  |||||||||||||*|||||**
Sbjct:       1226 ACTGTCTTCTCTCTCTCGTAC 1206
                  ||   ||||||    |||----------------------------------1
                  ||   ||||||    ||-----------------------------------3
                  ||   ||||||    |------------------------------------2
                  ||   ||||||-----------------------------------------2
                  ||   |||||------------------------------------------50
                  ||   ||||-------------------------------------------79
                  ||   |||--------------------------------------------3
                  ||   ||---------------------------------------------3
                  ||   |----------------------------------------------1
                  ||--------------------------------------------------1
                  |---------------------------------------------------1

>miR156a
    Score: 1.5  Deg: 9:1100:9:46   T_00080616

Query:          1 TGACAGAAGAGAGTGAGCAC
 21
                  |||||||||||||*||||||*
Sbjct:       1109 ACTGTCTTCTCTCTCTCGTGT 1089
                          ||   |    | |------------------------------1
                          ||   |    |--------------------------------1
                          ||   |-------------------------------------6
                          ||-----------------------------------------9
                          |------------------------------------------4

>miR156a
    Score: 1.5  Deg: 9:1250:9:33   T_00080613
```

```
Query:          1 TGACAGAAGAGAGTGAGCAC
 21
                  ||||||||||||*|||||*
Sbjct:       1259 ACTGTCTTCTCTCTCTCGTGT 1239
                          ||   |    | |-------------------------------1
                          ||   |    |---------------------------------1
                          ||   |-------------------------------------6
                          ||-----------------------------------------9
                          |------------------------------------------4

>miR156a
    Score: 1.5  Deg: 9:1195:9:64   T_00080614

Query:          1 TGACAGAAGAGAGTGAGCAC
 21
                  ||||||||||||*|||||*
Sbjct:       1204 ACTGTCTTCTCTCTCTCGTGT 1184
                          ||   |    | |-------------------------------1
                          ||   |    |---------------------------------1
                          ||   |-------------------------------------6
                          ||-----------------------------------------9
                          |------------------------------------------4

>miR156a
    Score: 1.5  Deg: 9:1124:9:46   T_00080615

Query:          1 TGACAGAAGAGAGTGAGCAC
 21
                  ||||||||||||*|||||*
Sbjct:       1133 ACTGTCTTCTCTCTCTCGTGT 1113
                          ||   |    | |-------------------------------1
                          ||   |    |---------------------------------1
                          ||   |-------------------------------------6
                          ||-----------------------------------------9
                          |------------------------------------------4

>miR156a
    Score: 1.5  Deg: 9:1250:9:64   T_00080612

Query:          1 TGACAGAAGAGAGTGAGCAC
 21
                  ||||||||||||*|||||*
Sbjct:       1259 ACTGTCTTCTCTCTCTCGTGT 1239
                          ||   |    | |-------------------------------1
```

```
                  ||    |    |--------------------------------1
                  ||    |-------------------------------------6
                  ||-----------------------------------------9
                  |------------------------------------------4

>miRl56a
    Score: 1.5  Deg: 12:1418:12:64 T_00046313

Query:          1 TGACAGAAGAGAGTGAGCAC
 21
                  ||||||||||||*|||||*
Sbjct:       1427 ACTGTCTTCTCTCTCTCGTGT 1407
                  |   |||  |   |  |------------------------------1
                  |   |||  |   |---------------------------------1
                  |   |||  |-------------------------------------4
                  |   |||----------------------------------------1
                  |   ||-----------------------------------------12
                  |   |------------------------------------------10
                  |----------------------------------------------2

>miRl56a
    Score: 1.5  Deg: 77:2106:83:749T_00052738

Query:          1 TGACAGAAGAGAGTGAGCAC
 21
                  ||||||||||||*|||||*
Sbjct:       2114 ACTGTCTTCTCTCTCTCGTGC 2094
                  | ||||||| |||--------------------------------1
                  | ||||||| ||---------------------------------1
                  | ||||||| |----------------------------------1
                  | |||||||------------------------------------1
                  | ||||||-------------------------------------1
                  | |||||--------------------------------------1
                  | ||||---------------------------------------1
                  | |||----------------------------------------9
                  | ||-----------------------------------------56
                  | |------------------------------------------77
                  |--------------------------------------------1

>miRl56a
    Score: 1.5  Deg: 77:443:77:327 T_00052739

Query:          1 TGACAGAAGAGAGTGAGCAC
 21
                  ||||||||||||*|||||*
```

```
Sbjct:        451 ACTGTCTTCTCTCTCTCGTGC 431
                        | ||||||| |||-------------------------------1
                        | ||||||| ||--------------------------------1
                        | ||||||| |---------------------------------1
                        | |||||||-----------------------------------1
                        | ||||||------------------------------------1
                        | |||||-------------------------------------1
                        | ||||--------------------------------------1
                        | |||---------------------------------------9
                        | ||----------------------------------------56
                        | |-----------------------------------------77
                        |-------------------------------------------1

>miR156a
    Score: 1.5  Deg: 77:2114:83:749T_00052737

Query:          1 TGACAGAAGAGAGTGAGCAC
 21
                  |||||||||||||*||||||*
Sbjct:       2122 ACTGTCTTCTCTCTCTCGTGC 2102
                        | ||||||| |||-------------------------------1
                        | ||||||| ||--------------------------------1
                        | ||||||| |---------------------------------1
                        | |||||||-----------------------------------1
                        | ||||||------------------------------------1
                        | |||||-------------------------------------1
                        | ||||--------------------------------------1
                        | |||---------------------------------------9
                        | ||----------------------------------------56
                        | |-----------------------------------------77
                        |-------------------------------------------1

>miR156a
    Score: 1.5  Deg: 5:2012:5:54   T_00071663

Query:          1 TGACAGAAGAGAGTGAGCAC
 21
                  |||||||||||||*||||||*
Sbjct:       2021 ACTGTCTTCTCTCTCTCGTGT 2001
                      |   ||     | |  |-----------------------------1
                      |   ||     | |--------------------------------1
                      |   ||     |----------------------------------1
                      |   ||----------------------------------------5
                      |   |-----------------------------------------4
                      |---------------------------------------------1
```

```
>miR156a
    Score: 1.5  Deg: 5:1901:5:45   T_00071657

Query:          1 TGACAGAAGAGAGTGAGCAC
 21
                  |||||||||||||*||||||*
Sbjct:       1910 ACTGTCTTCTCTCTCTCGTGT 1890
                      |   ||      | |  |-------------------------------1
                      |   ||      | |----------------------------------1
                      |   ||      |------------------------------------1
                      |   ||-------------------------------------------5
                      |   |--------------------------------------------4
                      |------------------------------------------------1

>miR156a
    Score: 1.5  Deg: 5:1222:5:44   T_00071664

Query:          1 TGACAGAAGAGAGTGAGCAC
 21
                  |||||||||||||*||||||*
Sbjct:       1231 ACTGTCTTCTCTCTCTCGTGT 1211
                      |   ||      | |  |-------------------------------1
                      |   ||      | |----------------------------------1
                      |   ||      |------------------------------------1
                      |   ||-------------------------------------------5
                      |   |--------------------------------------------4
                      |------------------------------------------------1

>miR156a
    Score: 1.5  Deg: 5:2528:631:10547  T_00071658

Query:          1 TGACAGAAGAGAGTGAGCAC
 21
                  |||||||||||||*||||||*
Sbjct:       2537 ACTGTCTTCTCTCTCTCGTGT 2517
                      |   ||      | |  |-------------------------------1
                      |   ||      | |----------------------------------1
                      |   ||      |------------------------------------1
                      |   ||-------------------------------------------5
                      |   |--------------------------------------------4
                      |------------------------------------------------1

>miR156a
    Score: 1.5  Deg: 5:1457:5:53   T_00071665
```

```
Query:          1 TGACAGAAGAGAGTGAGCAC
 21
                  ||||||||||||*||||||*
Sbjct:       1466 ACTGTCTTCTCTCTCTCGTGT 1446
                      |   ||     | |  |-------------------------------1
                      |   ||     | |---------------------------------1
                      |   ||     |-----------------------------------1
                      |   ||-----------------------------------------5
                      |   |------------------------------------------4
                      |----------------------------------------------1

>miR156a
    Score: 1.5  Deg: 5:2084:631:10538  T_00071656

Query:          1 TGACAGAAGAGAGTGAGCAC
 21
                  ||||||||||||*||||||*
Sbjct:       2093 ACTGTCTTCTCTCTCTCGTGT 2073
                      |   ||     | |  |-------------------------------1
                      |   ||     | |---------------------------------1
                      |   ||     |-----------------------------------1
                      |   ||-----------------------------------------5
                      |   |------------------------------------------4
                      |----------------------------------------------1

>miR156a
    Score: 1.5  Deg: 5:1826:5:53   T_00071661

Query:          1 TGACAGAAGAGAGTGAGCAC
 21
                  ||||||||||||*||||||*
Sbjct:       1835 ACTGTCTTCTCTCTCTCGTGT 1815
                      |   ||     | |  |-------------------------------1
                      |   ||     | |---------------------------------1
                      |   ||     |-----------------------------------1
                      |   ||-----------------------------------------5
                      |   |------------------------------------------4
                      |----------------------------------------------1

>miR156a
    Score: 1.5  Deg: 5:1160:5:44   T_00071667

Query:          1 TGACAGAAGAGAGTGAGCAC
 21
```

```
                  |||||||||||||*||||||*
Sbjct:       1169 ACTGTCTTCTCTCTCTCGTGT 1149
                      |   ||      | |  |--------------------------------1
                      |   ||      | |-----------------------------------1
                      |   ||      |-------------------------------------1
                      |   ||-------------------------------------------5
                      |   |--------------------------------------------4
                      |------------------------------------------------1

>miR156a
    Score: 1.5  Deg: 5:2345:5:54   T_00071659

Query:              1 TGACAGAAGAGAGTGAGCAC
 21
                  |||||||||||||*||||||*
Sbjct:       2354 ACTGTCTTCTCTCTCTCGTGT 2334
                      |   ||      | |  |--------------------------------1
                      |   ||      | |-----------------------------------1
                      |   ||      |-------------------------------------1
                      |   ||-------------------------------------------5
                      |   |--------------------------------------------4
                      |------------------------------------------------1

>miR156a
    Score: 1.5  Deg: 5:1931:631:10538  T_00071660

Query:              1 TGACAGAAGAGAGTGAGCAC
 21
                  |||||||||||||*||||||*
Sbjct:       1940 ACTGTCTTCTCTCTCTCGTGT 1920
                      |   ||      | |  |--------------------------------1
                      |   ||      | |-----------------------------------1
                      |   ||      |-------------------------------------1
                      |   ||-------------------------------------------5
                      |   |--------------------------------------------4
                      |------------------------------------------------1

>miR156a
    Score: 1.5  Deg: 5:2217:631:10547  T_00071662

Query:              1 TGACAGAAGAGAGTGAGCAC
 21
                  |||||||||||||*||||||*
Sbjct:       2226 ACTGTCTTCTCTCTCTCGTGT 2206
                      |   ||      | |  |--------------------------------1
```

```
                      |   ||     | |----------------------------------1
                      |   ||     |------------------------------------1
                      |   ||------------------------------------------5
                      |   |-------------------------------------------4
                      |-----------------------------------------------1

>miR156a
    Score: 1.5  Deg: 5:1161:5:44   T_00071666

Query:          1 TGACAGAAGAGAGTGAGCAC
 21
                  |||||||||||||*||||||*
Sbjct:       1170 ACTGTCTTCTCTCTCTCGTGT 1150
                      |   ||     | |  |-------------------------------1
                      |   ||     | |----------------------------------1
                      |   ||     |------------------------------------1
                      |   ||------------------------------------------5
                      |   |-------------------------------------------4
                      |-----------------------------------------------1

>miR156b-5p
    Score: 0.8  Deg: 10:1311:27:223T_00091783

Query:          1 TTGACAGAAGAGAGAGAGCAC
 22
                  :||||||||||||||||||||*
Sbjct:       1320 GACTGTCTTCTCTCTCTCGTGT 1299
                          ||||    |    |-------------------------------1
                          ||||    |------------------------------------2
                          ||||-----------------------------------------6
                          |||------------------------------------------6
                          ||-------------------------------------------10
                          |--------------------------------------------1

>miR156b-5p
    Score: 0.8  Deg: 10:1668:27:226T_00091779

Query:          1 TTGACAGAAGAGAGAGAGCAC
 22
                  :||||||||||||||||||||*
Sbjct:       1677 GACTGTCTTCTCTCTCTCGTGT 1656
                          ||||    |    |-------------------------------1
                          ||||    |------------------------------------2
                          ||||-----------------------------------------6
                          |||------------------------------------------6
```

```
                        ||------------------------------------------10
                        |-------------------------------------------1

>miR156b-5p
    Score: 0.8  Deg: 10:2823:27:234T_00091781

Query:          1 TTGACAGAAGAGAGAGAGCAC
 22
                  :||||||||||||||||||||*
Sbjct:       2832 GACTGTCTTCTCTCTCTCGTGT 2811
                        ||||    |    |------------------------------1
                        ||||    |-----------------------------------2
                        ||||----------------------------------------6
                        |||-----------------------------------------6
                        ||------------------------------------------10
                        |-------------------------------------------1

>miR156b-5p
    Score: 0.8  Deg: 10:1470:27:223T_00091782

Query:          1 TTGACAGAAGAGAGAGAGCAC
 22
                  :||||||||||||||||||||*
Sbjct:       1479 GACTGTCTTCTCTCTCTCGTGT 1458
                        ||||    |    |------------------------------1
                        ||||    |-----------------------------------2
                        ||||----------------------------------------6
                        |||-----------------------------------------6
                        ||------------------------------------------10
                        |-------------------------------------------1

>miR156b-5p
    Score: 0.8  Deg: 10:1199:27:223T_00091784

Query:          1 TTGACAGAAGAGAGAGAGCAC
 22
                  :||||||||||||||||||||*
Sbjct:       1208 GACTGTCTTCTCTCTCTCGTGT 1187
                        ||||    |    |------------------------------1
                        ||||    |-----------------------------------2
                        ||||----------------------------------------6
                        |||-----------------------------------------6
                        ||------------------------------------------10
                        |-------------------------------------------1
```

```
>miR156b-5p
    Score: 0.8  Deg: 10:1496:27:226T_00091780

Query:          1 TTGACAGAAGAGAGAGAGCAC
 22
                  :||||||||||||||||||||*
Sbjct:       1505 GACTGTCTTCTCTCTCTCGTGT 1484
                          ||||    |    |-------------------------------1
                          ||||    |------------------------------------2
                          ||||-----------------------------------------6
                          |||------------------------------------------6
                          ||-------------------------------------------10
                          |--------------------------------------------1

>miR156b-5p
    Score: 1.5  Deg: 22:1017:24:363T_00090332

Query:          1 TTGACAGAAGAGAGAGAGCAC
 22
                  |||||||||||*|||||||||*
Sbjct:       1026 AACTGTCTTCTATCTCTCGTGC 1005
                       | | ||||||  |-----------------------------------2
                       | | ||||||--------------------------------------1
                       | | |||||---------------------------------------1
                       | | ||||----------------------------------------1
                       | | |||-----------------------------------------2
                       | | ||------------------------------------------3
                       | | |-------------------------------------------22
                       | |---------------------------------------------1
                       |-----------------------------------------------2

>miR156b-5p
    Score: 1.5  Deg: 22:1088:24:371T_00090336

Query:          1 TTGACAGAAGAGAGAGAGCAC
 22
                  |||||||||||*|||||||||*
Sbjct:       1097 AACTGTCTTCTATCTCTCGTGC 1076
                       | | ||||||  |-----------------------------------2
                       | | ||||||--------------------------------------1
                       | | |||||---------------------------------------1
                       | | ||||----------------------------------------1
                       | | |||-----------------------------------------2
                       | | ||------------------------------------------3
                       | | |-------------------------------------------22
```

```
                       | |---------------------------------------------1
                       |-----------------------------------------------2

>miR156b-5p
    Score: 1.5  Deg: 22:1171:24:378T_00090333

Query:           1 TTGACAGAAGAGAGAGAGCAC
 22
                  |||||||||||*|||||||||*
Sbjct:        1180 AACTGTCTTCTATCTCTCGTGC 1159
                       | | ||||||  |-----------------------------------2
                       | | ||||||----------------------------------------1
                       | | |||||-----------------------------------------1
                       | | ||||------------------------------------------1
                       | | |||-------------------------------------------2
                       | | ||--------------------------------------------3
                       | | |---------------------------------------------22
                       | |-----------------------------------------------1
                       |-------------------------------------------------2

>miR156b-5p
    Score: 1.5  Deg: 22:1129:24:363T_00090331

Query:           1 TTGACAGAAGAGAGAGAGCAC
 22
                  |||||||||||*|||||||||*
Sbjct:        1138 AACTGTCTTCTATCTCTCGTGC 1117
                       | | ||||||  |-----------------------------------2
                       | | ||||||----------------------------------------1
                       | | |||||-----------------------------------------1
                       | | ||||------------------------------------------1
                       | | |||-------------------------------------------2
                       | | ||--------------------------------------------3
                       | | |---------------------------------------------22
                       | |-----------------------------------------------1
                       |-------------------------------------------------2

>miR156b-5p
    Score: 1.5  Deg: 22:958:24:363 T_00090335

Query:           1 TTGACAGAAGAGAGAGAGCAC
 22
                  |||||||||||*|||||||||*
Sbjct:         967 AACTGTCTTCTATCTCTCGTGC 946
                       | | ||||||  |-----------------------------------2
```

```
                      | | ||||||----------------------------------1
                      | | |||||-----------------------------------1
                      | | ||||------------------------------------1
                      | | |||-------------------------------------2
                      | | ||--------------------------------------3
                      | | |---------------------------------------22
                      | |-----------------------------------------1
                      |-------------------------------------------2

>miR156b-5p
    Score: 1.5  Deg: 22:941:24:361 T_00090334

Query:           1 TTGACAGAAGAGAGAGAGCAC
 22
                   |||||||||||*|||||||||*
Sbjct:         950 AACTGTCTTCTATCTCTCGTGC 929
                      | | ||||||  |-------------------------------2
                      | | ||||||----------------------------------1
                      | | |||||-----------------------------------1
                      | | ||||------------------------------------1
                      | | |||-------------------------------------2
                      | | ||--------------------------------------3
                      | | |---------------------------------------22
                      | |-----------------------------------------1
                      |-------------------------------------------2

>miR156b-5p
    Score: 1.5  Deg: 79:1218:79:519T_00059648

Query:           1 TTGACAGAAGAGAGAGAGCAC
 22
                   *||||||||||||||||||**
Sbjct:        1227 TACTGTCTTCTCTCTCTCGTAC 1206
                    ||   ||||||    |||--------------------------------1
                    ||   ||||||    ||---------------------------------3
                    ||   ||||||    |----------------------------------2
                    ||   ||||||---------------------------------------2
                    ||   |||||----------------------------------------50
                    ||   ||||-----------------------------------------79
                    ||   |||------------------------------------------3
                    ||   ||-------------------------------------------3
                    ||   |--------------------------------------------1
                    ||------------------------------------------------1
                    |-------------------------------------------------1
```

```
>miR156b-5p
    Score: 0.5  Deg: 9:1100:9:46   T_00080616

Query:          1 TTGACAGAAGAGAGAGAGCAC
 22
                  ||||||||||||||||||||*
Sbjct:       1110 AACTGTCTTCTCTCTCTCGTGT 1089
                           ||   |    | |------------------------------1
                           ||   |    |--------------------------------1
                           ||   |-------------------------------------6
                           ||-----------------------------------------9
                           |------------------------------------------4

>miR156b-5p
    Score: 0.5  Deg: 9:1250:9:33   T_00080613

Query:          1 TTGACAGAAGAGAGAGAGCAC
 22
                  ||||||||||||||||||||*
Sbjct:       1260 AACTGTCTTCTCTCTCTCGTGT 1239
                           ||   |    | |------------------------------1
                           ||   |    |--------------------------------1
                           ||   |-------------------------------------6
                           ||-----------------------------------------9
                           |------------------------------------------4

>miR156b-5p
    Score: 0.5  Deg: 9:1195:9:64   T_00080614

Query:          1 TTGACAGAAGAGAGAGAGCAC
 22
                  ||||||||||||||||||||*
Sbjct:       1205 AACTGTCTTCTCTCTCTCGTGT 1184
                           ||   |    | |------------------------------1
                           ||   |    |--------------------------------1
                           ||   |-------------------------------------6
                           ||-----------------------------------------9
                           |------------------------------------------4

>miR156b-5p
    Score: 0.5  Deg: 9:1124:9:46   T_00080615

Query:          1 TTGACAGAAGAGAGAGAGCAC
 22
                  ||||||||||||||||||||*
```

```
Sbjct:       1134 AACTGTCTTCTCTCTCTCGTGT 1113
                           ||    |    | |------------------------------1
                           ||    |    |--------------------------------1
                           ||    |-------------------------------------6
                           ||-----------------------------------------9
                           |------------------------------------------4
```

>miR156b-5p
    Score: 0.5  Deg: 9:1250:9:64   T_00080612

```
Query:          1 TTGACAGAAGAGAGAGAGCAC
 22
                  ||||||||||||||||||||*
Sbjct:       1260 AACTGTCTTCTCTCTCTCGTGT 1239
                           ||    |    | |------------------------------1
                           ||    |    |--------------------------------1
                           ||    |-------------------------------------6
                           ||-----------------------------------------9
                           |------------------------------------------4
```

>miR156b-5p
    Score: 1.0  Deg: 12:1418:12:64 T_00046313

```
Query:          1 TTGACAGAAGAGAGAGAGCAC
 22
                  *|||||||||||||||||||*
Sbjct:       1428 TACTGTCTTCTCTCTCTCGTGT 1407
                       |   |||  |   |  |------------------------------1
                       |   |||  |   |---------------------------------1
                       |   |||  |-------------------------------------4
                       |   |||----------------------------------------1
                       |   ||-----------------------------------------12
                       |   |------------------------------------------10
                       |----------------------------------------------2
```

>miR156b-5p
    Score: 0.5  Deg: 77:2106:83:749T_00052738

```
Query:          1 TTGACAGAAGAGAGAGAGCAC
 22
                  ||||||||||||||||||||*
Sbjct:       2115 AACTGTCTTCTCTCTCTCGTGC 2094
                         | ||||||| |||--------------------------------1
                         | ||||||| ||---------------------------------1
```

```
                         | ||||||| |---------------------------------1
                         | |||||||-----------------------------------1
                         | ||||||------------------------------------1
                         | |||||-------------------------------------1
                         | ||||--------------------------------------1
                         | |||---------------------------------------9
                         | ||----------------------------------------56
                         | |-----------------------------------------77
                         |-------------------------------------------1

>miR156b-5p
    Score: 0.5  Deg: 77:443:77:327 T_00052739

Query:          1 TTGACAGAAGAGAGAGAGCAC
 22
                  ||||||||||||||||||||*
Sbjct:        452 AACTGTCTTCTCTCTCTCGTGC 431
                         | ||||||| |||-------------------------------1
                         | ||||||| ||--------------------------------1
                         | ||||||| |---------------------------------1
                         | |||||||-----------------------------------1
                         | ||||||------------------------------------1
                         | |||||-------------------------------------1
                         | ||||--------------------------------------1
                         | |||---------------------------------------9
                         | ||----------------------------------------56
                         | |-----------------------------------------77
                         |-------------------------------------------1

>miR156b-5p
    Score: 0.5  Deg: 77:2114:83:749T_00052737

Query:          1 TTGACAGAAGAGAGAGAGCAC
 22
                  ||||||||||||||||||||*
Sbjct:       2123 AACTGTCTTCTCTCTCTCGTGC 2102
                         | ||||||| |||-------------------------------1
                         | ||||||| ||--------------------------------1
                         | ||||||| |---------------------------------1
                         | |||||||-----------------------------------1
                         | ||||||------------------------------------1
                         | |||||-------------------------------------1
                         | ||||--------------------------------------1
                         | |||---------------------------------------9
                         | ||----------------------------------------56
```

```
                     | |-----------------------------------------77
                     |-------------------------------------------1

>miR156b-5p
    Score: 1.0  Deg: 5:2012:5:54   T_00071663

Query:          1 TTGACAGAAGAGAGAGAGCAC
 22
                  *|||||||||||||||||||*
Sbjct:       2022 TACTGTCTTCTCTCTCTCGTGT 2001
                       |   ||      | |  |-------------------------------1
                       |   ||      | |----------------------------------1
                       |   ||      |------------------------------------1
                       |   ||-------------------------------------------5
                       |   |--------------------------------------------4
                       |------------------------------------------------1

>miR156b-5p
    Score: 1.0  Deg: 5:1901:5:45   T_00071657

Query:          1 TTGACAGAAGAGAGAGAGCAC
 22
                  *|||||||||||||||||||*
Sbjct:       1911 TACTGTCTTCTCTCTCTCGTGT 1890
                       |   ||      | |  |-------------------------------1
                       |   ||      | |----------------------------------1
                       |   ||      |------------------------------------1
                       |   ||-------------------------------------------5
                       |   |--------------------------------------------4
                       |------------------------------------------------1

>miR156b-5p
    Score: 1.0  Deg: 5:1222:5:44   T_00071664

Query:          1 TTGACAGAAGAGAGAGAGCAC
 22
                  *|||||||||||||||||||*
Sbjct:       1232 TACTGTCTTCTCTCTCTCGTGT 1211
                       |   ||      | |  |-------------------------------1
                       |   ||      | |----------------------------------1
                       |   ||      |------------------------------------1
                       |   ||-------------------------------------------5
                       |   |--------------------------------------------4
                       |------------------------------------------------1
```

```
>miR156b-5p
    Score: 1.0  Deg: 5:2528:631:10547  T_00071658

Query:          1 TTGACAGAAGAGAGAGAGCAC
 22
                  *|||||||||||||||||||*
Sbjct:       2538 TACTGTCTTCTCTCTCTCGTGT 2517
                       |   ||      | |  |------------------------------1
                       |   ||      | |----------------------------------1
                       |   ||      |------------------------------------1
                       |   ||-----------------------------------------5
                       |   |------------------------------------------4
                       |----------------------------------------------1

>miR156b-5p
    Score: 1.0  Deg: 5:1457:5:53   T_00071665

Query:          1 TTGACAGAAGAGAGAGAGCAC
 22
                  *|||||||||||||||||||*
Sbjct:       1467 TACTGTCTTCTCTCTCTCGTGT 1446
                       |   ||      | |  |------------------------------1
                       |   ||      | |----------------------------------1
                       |   ||      |------------------------------------1
                       |   ||-----------------------------------------5
                       |   |------------------------------------------4
                       |----------------------------------------------1

>miR156b-5p
    Score: 1.0  Deg: 5:2084:631:10538  T_00071656

Query:          1 TTGACAGAAGAGAGAGAGCAC
 22
                  *|||||||||||||||||||*
Sbjct:       2094 TACTGTCTTCTCTCTCTCGTGT 2073
                       |   ||      | |  |------------------------------1
                       |   ||      | |----------------------------------1
                       |   ||      |------------------------------------1
                       |   ||-----------------------------------------5
                       |   |------------------------------------------4
                       |----------------------------------------------1

>miR156b-5p
    Score: 1.0  Deg: 5:1826:5:53   T_00071661
```

```
Query:          1 TTGACAGAAGAGAGAGAGCAC
 22
                  *|||||||||||||||||||*
Sbjct:       1836 TACTGTCTTCTCTCTCTCGTGT 1815
                       |   ||      | |  |-------------------------------1
                       |   ||      | |----------------------------------1
                       |   ||      |------------------------------------1
                       |   ||-------------------------------------------5
                       |   |--------------------------------------------4
                       |------------------------------------------------1

>miR156b-5p
    Score: 1.0  Deg: 5:1160:5:44   T_00071667

Query:          1 TTGACAGAAGAGAGAGAGCAC
 22
                  *|||||||||||||||||||*
Sbjct:       1170 TACTGTCTTCTCTCTCTCGTGT 1149
                       |   ||      | |  |-------------------------------1
                       |   ||      | |----------------------------------1
                       |   ||      |------------------------------------1
                       |   ||-------------------------------------------5
                       |   |--------------------------------------------4
                       |------------------------------------------------1

>miR156b-5p
    Score: 1.0  Deg: 5:2345:5:54   T_00071659

Query:          1 TTGACAGAAGAGAGAGAGCAC
 22
                  *|||||||||||||||||||*
Sbjct:       2355 TACTGTCTTCTCTCTCTCGTGT 2334
                       |   ||      | |  |-------------------------------1
                       |   ||      | |----------------------------------1
                       |   ||      |------------------------------------1
                       |   ||-------------------------------------------5
                       |   |--------------------------------------------4
                       |------------------------------------------------1

>miR156b-5p
    Score: 1.0  Deg: 5:1931:631:10538  T_00071660

Query:          1 TTGACAGAAGAGAGAGAGCAC
 22
                  *|||||||||||||||||||*
```

```
Sbjct:       1941 TACTGTCTTCTCTCTCTCGTGT 1920
                       |    ||      | |  |-------------------------------1
                       |    ||      | |----------------------------------1
                       |    ||      |------------------------------------1
                       |    ||------------------------------------------5
                       |    |-------------------------------------------4
                       |-----------------------------------------------1

>miR156b-5p
    Score: 1.0  Deg: 5:2217:631:10547  T_00071662

Query:          1 TTGACAGAAGAGAGAGAGCAC
 22
                  *|||||||||||||||||||*
Sbjct:       2227 TACTGTCTTCTCTCTCTCGTGT 2206
                       |    ||      | |  |-------------------------------1
                       |    ||      | |----------------------------------1
                       |    ||      |------------------------------------1
                       |    ||------------------------------------------5
                       |    |-------------------------------------------4
                       |-----------------------------------------------1

>miR156b-5p
    Score: 1.0  Deg: 5:1161:5:44   T_00071666

Query:          1 TTGACAGAAGAGAGAGAGCAC
 22
                  *|||||||||||||||||||*
Sbjct:       1171 TACTGTCTTCTCTCTCTCGTGT 1150
                       |    ||      | |  |-------------------------------1
                       |    ||      | |----------------------------------1
                       |    ||      |------------------------------------1
                       |    ||------------------------------------------5
                       |    |-------------------------------------------4
                       |-----------------------------------------------1

>miR156c-5p
    Score: 0.8  Deg: 10:1311:27:223T_00091783

Query:          1 TTGACAGAAGAGAGAGAGCAC
 22
                  :|||||||||||||||||||*
Sbjct:       1320 GACTGTCTTCTCTCTCTCGTGT 1299
                          ||||    |    |-------------------------------1
                          ||||    |-----------------------------------2
```

```
                          ||||-----------------------------------------6
                          |||------------------------------------------6
                          ||-------------------------------------------10
                          |--------------------------------------------1

>miR156c-5p
    Score: 0.8  Deg: 10:1668:27:226T_00091779

Query:          1 TTGACAGAAGAGAGAGAGCAC
 22
                  :||||||||||||||||||||*
Sbjct:       1677 GACTGTCTTCTCTCTCTCGTGT 1656
                          ||||    |    |-------------------------------1
                          ||||    |------------------------------------2
                          ||||-----------------------------------------6
                          |||------------------------------------------6
                          ||-------------------------------------------10
                          |--------------------------------------------1

>miR156c-5p
    Score: 0.8  Deg: 10:2823:27:234T_00091781

Query:          1 TTGACAGAAGAGAGAGAGCAC
 22
                  :||||||||||||||||||||*
Sbjct:       2832 GACTGTCTTCTCTCTCTCGTGT 2811
                          ||||    |    |-------------------------------1
                          ||||    |------------------------------------2
                          ||||-----------------------------------------6
                          |||------------------------------------------6
                          ||-------------------------------------------10
                          |--------------------------------------------1

>miR156c-5p
    Score: 0.8  Deg: 10:1470:27:223T_00091782

Query:          1 TTGACAGAAGAGAGAGAGCAC
 22
                  :||||||||||||||||||||*
Sbjct:       1479 GACTGTCTTCTCTCTCTCGTGT 1458
                          ||||    |    |-------------------------------1
                          ||||    |------------------------------------2
                          ||||-----------------------------------------6
                          |||------------------------------------------6
                          ||-------------------------------------------10
```

```
                         |--------------------------------------------1

>miR156c-5p
    Score: 0.8  Deg: 10:1199:27:223T_00091784

Query:          1 TTGACAGAAGAGAGAGAGCAC
 22
                  :||||||||||||||||||||*
Sbjct:       1208 GACTGTCTTCTCTCTCTCGTGT 1187
                         ||||    |    |-------------------------------1
                         ||||    |------------------------------------2
                         ||||-----------------------------------------6
                         |||------------------------------------------6
                         ||-------------------------------------------10
                         |--------------------------------------------1

>miR156c-5p
    Score: 0.8  Deg: 10:1496:27:226T_00091780

Query:          1 TTGACAGAAGAGAGAGAGCAC
 22
                  :||||||||||||||||||||*
Sbjct:       1505 GACTGTCTTCTCTCTCTCGTGT 1484
                         ||||    |    |-------------------------------1
                         ||||    |------------------------------------2
                         ||||-----------------------------------------6
                         |||------------------------------------------6
                         ||-------------------------------------------10
                         |--------------------------------------------1

>miR156c-5p
    Score: 1.5  Deg: 22:1017:24:363T_00090332

Query:          1 TTGACAGAAGAGAGAGAGCAC
 22
                  |||||||||||*|||||||||*
Sbjct:       1026 AACTGTCTTCTATCTCTCGTGC 1005
                      | | ||||||  |-----------------------------------2
                      | | ||||||--------------------------------------1
                      | | |||||---------------------------------------1
                      | | ||||----------------------------------------1
                      | | |||-----------------------------------------2
                      | | ||------------------------------------------3
                      | | |-------------------------------------------22
                      | |---------------------------------------------1
```

```
                       |-----------------------------------------------2

>miR156c-5p
    Score: 1.5  Deg: 22:1088:24:371T_00090336

Query:          1 TTGACAGAAGAGAGAGAGCAC
 22
                  |||||||||||*|||||||||*
Sbjct:       1097 AACTGTCTTCTATCTCTCGTGC 1076
                       | | ||||||  |-----------------------------------2
                       | | ||||||--------------------------------------1
                       | | |||||---------------------------------------1
                       | | ||||----------------------------------------1
                       | | |||-----------------------------------------2
                       | | ||------------------------------------------3
                       | | |-------------------------------------------22
                       | |---------------------------------------------1
                       |-----------------------------------------------2

>miR156c-5p
    Score: 1.5  Deg: 22:1171:24:378T_00090333

Query:          1 TTGACAGAAGAGAGAGAGCAC
 22
                  |||||||||||*|||||||||*
Sbjct:       1180 AACTGTCTTCTATCTCTCGTGC 1159
                       | | ||||||  |-----------------------------------2
                       | | ||||||--------------------------------------1
                       | | |||||---------------------------------------1
                       | | ||||----------------------------------------1
                       | | |||-----------------------------------------2
                       | | ||------------------------------------------3
                       | | |-------------------------------------------22
                       | |---------------------------------------------1
                       |-----------------------------------------------2

>miR156c-5p
    Score: 1.5  Deg: 22:1129:24:363T_00090331

Query:          1 TTGACAGAAGAGAGAGAGCAC
 22
                  |||||||||||*|||||||||*
Sbjct:       1138 AACTGTCTTCTATCTCTCGTGC 1117
                       | | ||||||  |-----------------------------------2
                       | | ||||||--------------------------------------1
```

```
                       | | |||||--------------------------------------1
                       | | ||||---------------------------------------1
                       | | |||----------------------------------------2
                       | | ||-----------------------------------------3
                       | | |------------------------------------------22
                       | |--------------------------------------------1
                       |----------------------------------------------2

>miR156c-5p
    Score: 1.5  Deg: 22:958:24:363 T_00090335

Query:          1 TTGACAGAAGAGAGAGAGCAC
 22
                  |||||||||||*|||||||||*
Sbjct:        967 AACTGTCTTCTATCTCTCGTGC 946
                       | | ||||||  |----------------------------------2
                       | | ||||||---------------------------------------1
                       | | |||||--------------------------------------1
                       | | ||||---------------------------------------1
                       | | |||----------------------------------------2
                       | | ||-----------------------------------------3
                       | | |------------------------------------------22
                       | |--------------------------------------------1
                       |----------------------------------------------2

>miR156c-5p
    Score: 1.5  Deg: 22:941:24:361 T_00090334

Query:          1 TTGACAGAAGAGAGAGAGCAC
 22
                  |||||||||||*|||||||||*
Sbjct:        950 AACTGTCTTCTATCTCTCGTGC 929
                       | | ||||||  |----------------------------------2
                       | | ||||||---------------------------------------1
                       | | |||||--------------------------------------1
                       | | ||||---------------------------------------1
                       | | |||----------------------------------------2
                       | | ||-----------------------------------------3
                       | | |------------------------------------------22
                       | |--------------------------------------------1
                       |----------------------------------------------2

>miR156c-5p
    Score: 1.5  Deg: 79:1218:79:519T_00059648
```

```
Query:          1 TTGACAGAAGAGAGAGAGCAC
 22
                  *|||||||||||||||||||**
Sbjct:       1227 TACTGTCTTCTCTCTCTCGTAC 1206
                   ||   ||||||    |||----------------------------------1
                   ||   ||||||    ||-----------------------------------3
                   ||   ||||||    |------------------------------------2
                   ||   ||||||-----------------------------------------2
                   ||   |||||------------------------------------------50
                   ||   ||||-------------------------------------------79
                   ||   |||--------------------------------------------3
                   ||   ||---------------------------------------------3
                   ||   |----------------------------------------------1
                   ||--------------------------------------------------1
                   |---------------------------------------------------1
```

```
>miR156c-5p
    Score: 0.5  Deg: 9:1100:9:46   T_00080616
```

```
Query:          1 TTGACAGAAGAGAGAGAGCAC
 22
                  ||||||||||||||||||||*
Sbjct:       1110 AACTGTCTTCTCTCTCTCGTGT 1089
                           ||    |    | |-------------------------------1
                           ||    |    |---------------------------------1
                           ||    |--------------------------------------6
                           ||-------------------------------------------9
                           |--------------------------------------------4
```

```
>miR156c-5p
    Score: 0.5  Deg: 9:1250:9:33   T_00080613
```

```
Query:          1 TTGACAGAAGAGAGAGAGCAC
 22
                  ||||||||||||||||||||*
Sbjct:       1260 AACTGTCTTCTCTCTCTCGTGT 1239
                           ||    |    | |-------------------------------1
                           ||    |    |---------------------------------1
                           ||    |--------------------------------------6
                           ||-------------------------------------------9
                           |--------------------------------------------4
```

```
>miR156c-5p
    Score: 0.5  Deg: 9:1195:9:64   T_00080614
```

```
Query:          1 TTGACAGAAGAGAGAGAGCAC
 22
                  ||||||||||||||||||||*
Sbjct:       1205 AACTGTCTTCTCTCTCTCGTGT 1184
                           ||   |    | |-------------------------------1
                           ||   |    |---------------------------------1
                           ||   |--------------------------------------6
                           ||------------------------------------------9
                           |-------------------------------------------4

>miR156c-5p
    Score: 0.5  Deg: 9:1124:9:46   T_00080615

Query:          1 TTGACAGAAGAGAGAGAGCAC
 22
                  ||||||||||||||||||||*
Sbjct:       1134 AACTGTCTTCTCTCTCTCGTGT 1113
                           ||   |    | |-------------------------------1
                           ||   |    |---------------------------------1
                           ||   |--------------------------------------6
                           ||------------------------------------------9
                           |-------------------------------------------4

>miR156c-5p
    Score: 0.5  Deg: 9:1250:9:64   T_00080612

Query:          1 TTGACAGAAGAGAGAGAGCAC
 22
                  ||||||||||||||||||||*
Sbjct:       1260 AACTGTCTTCTCTCTCTCGTGT 1239
                           ||   |    | |-------------------------------1
                           ||   |    |---------------------------------1
                           ||   |--------------------------------------6
                           ||------------------------------------------9
                           |-------------------------------------------4

>miR156c-5p
    Score: 1.0  Deg: 12:1418:12:64 T_00046313

Query:          1 TTGACAGAAGAGAGAGAGCAC
 22
                  *|||||||||||||||||||*
Sbjct:       1428 TACTGTCTTCTCTCTCTCGTGT 1407
                       |   |||  |   |  |-------------------------------1
                       |   |||  |   |---------------------------------1
```

```
                         |    |||   |------------------------------------4
                         |    |||----------------------------------------1
                         |    ||-----------------------------------------12
                         |    |------------------------------------------10
                         |-----------------------------------------------2
```

```
>miR156c-5p
    Score: 0.5  Deg: 77:2106:83:749T_00052738

Query:          1 TTGACAGAAGAGAGAGAGCAC
 22
                  ||||||||||||||||||||*
Sbjct:       2115 AACTGTCTTCTCTCTCTCGTGC 2094
                        | ||||||| |||----------------------------------1
                        | ||||||| ||-----------------------------------1
                        | ||||||| |------------------------------------1
                        | |||||||--------------------------------------1
                        | ||||||---------------------------------------1
                        | |||||----------------------------------------1
                        | ||||-----------------------------------------1
                        | |||------------------------------------------9
                        | ||-------------------------------------------56
                        | |--------------------------------------------77
                        |----------------------------------------------1

>miR156c-5p
    Score: 0.5  Deg: 77:443:77:327 T_00052739

Query:          1 TTGACAGAAGAGAGAGAGCAC
 22
                  ||||||||||||||||||||*
Sbjct:        452 AACTGTCTTCTCTCTCTCGTGC 431
                        | ||||||| |||----------------------------------1
                        | ||||||| ||-----------------------------------1
                        | ||||||| |------------------------------------1
                        | |||||||--------------------------------------1
                        | ||||||---------------------------------------1
                        | |||||----------------------------------------1
                        | ||||-----------------------------------------1
                        | |||------------------------------------------9
                        | ||-------------------------------------------56
                        | |--------------------------------------------77
                        |----------------------------------------------1
```

```
>miR156c-5p
    Score: 0.5  Deg: 77:2114:83:749T_00052737

Query:          1 TTGACAGAAGAGAGAGAGCAC
 22
                  |||||||||||||||||||||*
Sbjct:       2123 AACTGTCTTCTCTCTCTCGTGC 2102
                         | ||||||| |||---------------------------------1
                         | ||||||| ||----------------------------------1
                         | ||||||| |-----------------------------------1
                         | |||||||-------------------------------------1
                         | ||||||--------------------------------------1
                         | |||||---------------------------------------1
                         | ||||----------------------------------------1
                         | |||-----------------------------------------9
                         | ||------------------------------------------56
                         | |-------------------------------------------77
                         |---------------------------------------------1

>miR156c-5p
    Score: 1.0  Deg: 5:2012:5:54   T_00071663

Query:          1 TTGACAGAAGAGAGAGAGCAC
 22
                  *||||||||||||||||||||*
Sbjct:       2022 TACTGTCTTCTCTCTCTCGTGT 2001
                       |   ||     | |  |-------------------------------1
                       |   ||     | |----------------------------------1
                       |   ||     |------------------------------------1
                       |   ||------------------------------------------5
                       |   |-------------------------------------------4
                       |-----------------------------------------------1

>miR156c-5p
    Score: 1.0  Deg: 5:1901:5:45   T_00071657

Query:          1 TTGACAGAAGAGAGAGAGCAC
 22
                  *||||||||||||||||||||*
Sbjct:       1911 TACTGTCTTCTCTCTCTCGTGT 1890
                       |   ||     | |  |-------------------------------1
                       |   ||     | |----------------------------------1
                       |   ||     |------------------------------------1
                       |   ||------------------------------------------5
                       |   |-------------------------------------------4
```

```
                       |-----------------------------------------------1

>miR156c-5p
    Score: 1.0 Deg: 5:1222:5:44   T_00071664

Query:          1 TTGACAGAAGAGAGAGAGCAC
 22
                  *||||||||||||||||||||*
Sbjct:       1232 TACTGTCTTCTCTCTCTCGTGT 1211
                       |   ||     | |  |-------------------------------1
                       |   ||     | |----------------------------------1
                       |   ||     |------------------------------------1
                       |   ||------------------------------------------5
                       |   |-------------------------------------------4
                       |-----------------------------------------------1

>miR156c-5p
    Score: 1.0 Deg: 5:2528:631:10547  T_00071658

Query:          1 TTGACAGAAGAGAGAGAGCAC
 22
                  *||||||||||||||||||||*
Sbjct:       2538 TACTGTCTTCTCTCTCTCGTGT 2517
                       |   ||     | |  |-------------------------------1
                       |   ||     | |----------------------------------1
                       |   ||     |------------------------------------1
                       |   ||------------------------------------------5
                       |   |-------------------------------------------4
                       |-----------------------------------------------1

>miR156c-5p
    Score: 1.0 Deg: 5:1457:5:53   T_00071665

Query:          1 TTGACAGAAGAGAGAGAGCAC
 22
                  *||||||||||||||||||||*
Sbjct:       1467 TACTGTCTTCTCTCTCTCGTGT 1446
                       |   ||     | |  |-------------------------------1
                       |   ||     | |----------------------------------1
                       |   ||     |------------------------------------1
                       |   ||------------------------------------------5
                       |   |-------------------------------------------4
                       |-----------------------------------------------1

>miR156c-5p
```

```
    Score: 1.0  Deg: 5:2084:631:10538  T_00071656

Query:          1 TTGACAGAAGAGAGAGAGCAC
 22
                  *|||||||||||||||||||*
Sbjct:       2094 TACTGTCTTCTCTCTCTCGTGT 2073
                       |   ||      | |  |-------------------------------1
                       |   ||      | |-----------------------------------1
                       |   ||      |-------------------------------------1
                       |   ||-------------------------------------------5
                       |   |--------------------------------------------4
                       |------------------------------------------------1

>miR156c-5p
    Score: 1.0  Deg: 5:1826:5:53   T_00071661

Query:          1 TTGACAGAAGAGAGAGAGCAC
 22
                  *|||||||||||||||||||*
Sbjct:       1836 TACTGTCTTCTCTCTCTCGTGT 1815
                       |   ||      | |  |-------------------------------1
                       |   ||      | |-----------------------------------1
                       |   ||      |-------------------------------------1
                       |   ||-------------------------------------------5
                       |   |--------------------------------------------4
                       |------------------------------------------------1

>miR156c-5p
    Score: 1.0  Deg: 5:1160:5:44   T_00071667

Query:          1 TTGACAGAAGAGAGAGAGCAC
 22
                  *|||||||||||||||||||*
Sbjct:       1170 TACTGTCTTCTCTCTCTCGTGT 1149
                       |   ||      | |  |-------------------------------1
                       |   ||      | |-----------------------------------1
                       |   ||      |-------------------------------------1
                       |   ||-------------------------------------------5
                       |   |--------------------------------------------4
                       |------------------------------------------------1

>miR156c-5p
    Score: 1.0  Deg: 5:2345:5:54   T_00071659

Query:          1 TTGACAGAAGAGAGAGAGCAC
```

```
 22
                 *|||||||||||||||||||*
Sbjct:       2355 TACTGTCTTCTCTCTCTCGTGT 2334
                       |    ||      | |  |--------------------------------1
                       |    ||      | |-----------------------------------1
                       |    ||      |-------------------------------------1
                       |    ||-------------------------------------------5
                       |    |--------------------------------------------4
                       |-------------------------------------------------1

>miR156c-5p
    Score: 1.0  Deg: 5:1931:631:10538  T_00071660

Query:           1 TTGACAGAAGAGAGAGAGCAC
 22
                 *|||||||||||||||||||*
Sbjct:       1941 TACTGTCTTCTCTCTCTCGTGT 1920
                       |    ||      | |  |--------------------------------1
                       |    ||      | |-----------------------------------1
                       |    ||      |-------------------------------------1
                       |    ||-------------------------------------------5
                       |    |--------------------------------------------4
                       |-------------------------------------------------1

>miR156c-5p
    Score: 1.0  Deg: 5:2217:631:10547  T_00071662

Query:           1 TTGACAGAAGAGAGAGAGCAC
 22
                 *|||||||||||||||||||*
Sbjct:       2227 TACTGTCTTCTCTCTCTCGTGT 2206
                       |    ||      | |  |--------------------------------1
                       |    ||      | |-----------------------------------1
                       |    ||      |-------------------------------------1
                       |    ||-------------------------------------------5
                       |    |--------------------------------------------4
                       |-------------------------------------------------1

>miR156c-5p
    Score: 1.0  Deg: 5:1161:5:44   T_00071666

Query:           1 TTGACAGAAGAGAGAGAGCAC
 22
                 *|||||||||||||||||||*
Sbjct:       1171 TACTGTCTTCTCTCTCTCGTGT 1150
```

```
                                  |    ||      | |  |------------------------------1
                                  |    ||      | |---------------------------------1
                                  |    ||      |-----------------------------------1
                                  |    ||-----------------------------------------5
                                  |    |------------------------------------------4
                                  |-----------------------------------------------1
```

```
>miR156d-5p
    Score: 1.5  Deg: 10:1311:27:223T_00091783

Query:          1 TGACAGAAGAGAGTGAGCAC
 21
                  |||||||||||||*||||||*
Sbjct:       1319 ACTGTCTTCTCTCTCTCGTGT 1299
                         ||||    |    |------------------------------1
                         ||||    |-----------------------------------2
                         ||||-----------------------------------------6
                         |||------------------------------------------6
                         ||-------------------------------------------10
                         |--------------------------------------------1
```

```
>miR156d-5p
    Score: 1.5  Deg: 10:1668:27:226T_00091779

Query:          1 TGACAGAAGAGAGTGAGCAC
 21
                  |||||||||||||*||||||*
Sbjct:       1676 ACTGTCTTCTCTCTCTCGTGT 1656
                         ||||    |    |------------------------------1
                         ||||    |-----------------------------------2
                         ||||-----------------------------------------6
                         |||------------------------------------------6
                         ||-------------------------------------------10
                         |--------------------------------------------1
```

```
>miR156d-5p
    Score: 1.5  Deg: 10:2823:27:234T_00091781

Query:          1 TGACAGAAGAGAGTGAGCAC
 21
                  |||||||||||||*||||||*
Sbjct:       2831 ACTGTCTTCTCTCTCTCGTGT 2811
                         ||||    |    |------------------------------1
                         ||||    |-----------------------------------2
                         ||||-----------------------------------------6
```

```
                       |||------------------------------------------6
                       ||-------------------------------------------10
                       |--------------------------------------------1

>miR156d-5p
    Score: 1.5  Deg: 10:1470:27:223T_00091782

Query:          1 TGACAGAAGAGAGTGAGCAC
 21
                  |||||||||||||*||||||*
Sbjct:       1478 ACTGTCTTCTCTCTCTCGTGT 1458
                       ||||     |    |------------------------------1
                       ||||     |-----------------------------------2
                       ||||-----------------------------------------6
                       |||------------------------------------------6
                       ||-------------------------------------------10
                       |--------------------------------------------1

>miR156d-5p
    Score: 1.5  Deg: 10:1199:27:223T_00091784

Query:          1 TGACAGAAGAGAGTGAGCAC
 21
                  |||||||||||||*||||||*
Sbjct:       1207 ACTGTCTTCTCTCTCTCGTGT 1187
                       ||||     |    |------------------------------1
                       ||||     |-----------------------------------2
                       ||||-----------------------------------------6
                       |||------------------------------------------6
                       ||-------------------------------------------10
                       |--------------------------------------------1

>miR156d-5p
    Score: 1.5  Deg: 10:1496:27:226T_00091780

Query:          1 TGACAGAAGAGAGTGAGCAC
 21
                  |||||||||||||*||||||*
Sbjct:       1504 ACTGTCTTCTCTCTCTCGTGT 1484
                       ||||     |    |------------------------------1
                       ||||     |-----------------------------------2
                       ||||-----------------------------------------6
                       |||------------------------------------------6
                       ||-------------------------------------------10
                       |--------------------------------------------1
```

```
>miR156d-5p
    Score: 2.5  Deg: 22:1017:24:363T_00090332

Query:          1 TGACAGAAGAGAGTGAGCAC
 21
                  ||||||||||*||*||||||*
Sbjct:       1025 ACTGTCTTCTATCTCTCGTGC 1005
                       | | ||||||  |-----------------------------------2
                       | | ||||||--------------------------------------1
                       | | |||||---------------------------------------1
                       | | ||||----------------------------------------1
                       | | |||-----------------------------------------2
                       | | ||------------------------------------------3
                       | | |-------------------------------------------22
                       | |---------------------------------------------1
                       |-----------------------------------------------2

>miR156d-5p
    Score: 2.5  Deg: 22:1088:24:371T_00090336

Query:          1 TGACAGAAGAGAGTGAGCAC
 21
                  ||||||||||*||*||||||*
Sbjct:       1096 ACTGTCTTCTATCTCTCGTGC 1076
                       | | ||||||  |-----------------------------------2
                       | | ||||||--------------------------------------1
                       | | |||||---------------------------------------1
                       | | ||||----------------------------------------1
                       | | |||-----------------------------------------2
                       | | ||------------------------------------------3
                       | | |-------------------------------------------22
                       | |---------------------------------------------1
                       |-----------------------------------------------2

>miR156d-5p
    Score: 2.5  Deg: 22:1171:24:378T_00090333

Query:          1 TGACAGAAGAGAGTGAGCAC
 21
                  ||||||||||*||*||||||*
Sbjct:       1179 ACTGTCTTCTATCTCTCGTGC 1159
                       | | ||||||  |-----------------------------------2
                       | | ||||||--------------------------------------1
                       | | |||||---------------------------------------1
```

```
                    | | ||||-----------------------------------------1
                    | | |||------------------------------------------2
                    | | ||-------------------------------------------3
                    | | |--------------------------------------------22
                    | |----------------------------------------------1
                    |------------------------------------------------2
```

>miR156d-5p
Score: 2.5 Deg: 22:1129:24:363T_00090331

```
Query:          1 TGACAGAAGAGAGTGAGCAC
 21
                  ||||||||||*||*|||||*
Sbjct:       1137 ACTGTCTTCTATCTCTCGTGC 1117
                    | | ||||||  |-----------------------------------2
                    | | ||||||--------------------------------------1
                    | | |||||---------------------------------------1
                    | | ||||----------------------------------------1
                    | | |||-----------------------------------------2
                    | | ||------------------------------------------3
                    | | |-------------------------------------------22
                    | |---------------------------------------------1
                    |-----------------------------------------------2
```

>miR156d-5p
Score: 2.5 Deg: 22:958:24:363 T_00090335

```
Query:          1 TGACAGAAGAGAGTGAGCAC
 21
                  ||||||||||*||*|||||*
Sbjct:        966 ACTGTCTTCTATCTCTCGTGC 946
                    | | ||||||  |-----------------------------------2
                    | | ||||||--------------------------------------1
                    | | |||||---------------------------------------1
                    | | ||||----------------------------------------1
                    | | |||-----------------------------------------2
                    | | ||------------------------------------------3
                    | | |-------------------------------------------22
                    | |---------------------------------------------1
                    |-----------------------------------------------2
```

>miR156d-5p
Score: 2.5 Deg: 22:941:24:361 T_00090334

```
Query:          1 TGACAGAAGAGAGTGAGCAC
```

```
 21
                  ||||||||||*||*||||||*
Sbjct:        949 ACTGTCTTCTATCTCTCGTGC 929
                      | | ||||||  |----------------------------------2
                      | | ||||||---------------------------------------1
                      | | |||||----------------------------------------1
                      | | ||||-----------------------------------------1
                      | | |||------------------------------------------2
                      | | ||-------------------------------------------3
                      | | |--------------------------------------------22
                      | |----------------------------------------------1
                      |------------------------------------------------2

>miR156d-5p
    Score: 2.0  Deg: 79:1218:79:519T_00059648

Query:          1 TGACAGAAGAGAGTGAGCAC
 21
                  ||||||||||||*|||||**
Sbjct:       1226 ACTGTCTTCTCTCTCTCGTAC 1206
                  ||   ||||||    |||---------------------------------1
                  ||   ||||||    ||----------------------------------3
                  ||   ||||||    |-----------------------------------2
                  ||   ||||||----------------------------------------2
                  ||   |||||-----------------------------------------50
                  ||   ||||------------------------------------------79
                  ||   |||-------------------------------------------3
                  ||   ||--------------------------------------------3
                  ||   |---------------------------------------------1
                  ||-------------------------------------------------1
                  |--------------------------------------------------1

>miR156d-5p
    Score: 1.5  Deg: 9:1100:9:46   T_00080616

Query:          1 TGACAGAAGAGAGTGAGCAC
 21
                  ||||||||||||*||||||*
Sbjct:       1109 ACTGTCTTCTCTCTCTCGTGT 1089
                          ||   |     | |-------------------------------1
                          ||   |     |---------------------------------1
                          ||   |---------------------------------------6
                          ||-------------------------------------------9
                          |--------------------------------------------4
```

```
>miR156d-5p
    Score: 1.5  Deg: 9:1250:9:33   T_00080613

Query:          1 TGACAGAAGAGAGTGAGCAC
 21
                  ||||||||||||*|||||*
Sbjct:       1259 ACTGTCTTCTCTCTCTCGTGT 1239
                          ||   |    | |-------------------------------1
                          ||   |    |---------------------------------1
                          ||   |--------------------------------------6
                          ||------------------------------------------9
                          |-------------------------------------------4

>miR156d-5p
    Score: 1.5  Deg: 9:1195:9:64   T_00080614

Query:          1 TGACAGAAGAGAGTGAGCAC
 21
                  ||||||||||||*|||||*
Sbjct:       1204 ACTGTCTTCTCTCTCTCGTGT 1184
                          ||   |    | |-------------------------------1
                          ||   |    |---------------------------------1
                          ||   |--------------------------------------6
                          ||------------------------------------------9
                          |-------------------------------------------4

>miR156d-5p
    Score: 1.5  Deg: 9:1124:9:46   T_00080615

Query:          1 TGACAGAAGAGAGTGAGCAC
 21
                  ||||||||||||*|||||*
Sbjct:       1133 ACTGTCTTCTCTCTCTCGTGT 1113
                          ||   |    | |-------------------------------1
                          ||   |    |---------------------------------1
                          ||   |--------------------------------------6
                          ||------------------------------------------9
                          |-------------------------------------------4

>miR156d-5p
    Score: 1.5  Deg: 9:1250:9:64   T_00080612

Query:          1 TGACAGAAGAGAGTGAGCAC
 21
                  ||||||||||||*|||||*
```

```
Sbjct:       1259 ACTGTCTTCTCTCTCTCGTGT 1239
                          ||    |     | |------------------------------1
                          ||    |     |--------------------------------1
                          ||    |-------------------------------------6
                          ||-----------------------------------------9
                          |------------------------------------------4

>miR156d-5p
    Score: 1.5  Deg: 12:1418:12:64 T_00046313

Query:          1 TGACAGAAGAGAGTGAGCAC
 21
                  |||||||||||||*||||||*
Sbjct:       1427 ACTGTCTTCTCTCTCTCGTGT 1407
                      |   |||  |    |  |-------------------------------1
                      |   |||  |    |---------------------------------1
                      |   |||  |--------------------------------------4
                      |   |||-----------------------------------------1
                      |   ||------------------------------------------12
                      |   |-------------------------------------------10
                      |-----------------------------------------------2

>miR156d-5p
    Score: 1.5  Deg: 77:2106:83:749T_00052738

Query:          1 TGACAGAAGAGAGTGAGCAC
 21
                  |||||||||||||*||||||*
Sbjct:       2114 ACTGTCTTCTCTCTCTCGTGC 2094
                        | ||||||| |||--------------------------------1
                        | ||||||| ||---------------------------------1
                        | ||||||| |----------------------------------1
                        | |||||||------------------------------------1
                        | ||||||-------------------------------------1
                        | |||||--------------------------------------1
                        | ||||---------------------------------------1
                        | |||----------------------------------------9
                        | ||-----------------------------------------56
                        | |------------------------------------------77
                        |--------------------------------------------1

>miR156d-5p
    Score: 1.5  Deg: 77:443:77:327 T_00052739

Query:          1 TGACAGAAGAGAGTGAGCAC
```

```
 21
                  |||||||||||||*||||||*
Sbjct:        451 ACTGTCTTCTCTCTCTCGTGC 431
                        | ||||||| |||--------------------------------1
                        | ||||||| ||---------------------------------1
                        | ||||||| |----------------------------------1
                        | |||||||------------------------------------1
                        | ||||||-------------------------------------1
                        | |||||--------------------------------------1
                        | ||||---------------------------------------1
                        | |||----------------------------------------9
                        | ||-----------------------------------------56
                        | |------------------------------------------77
                        |--------------------------------------------1
```

```
>miR156d-5p
    Score: 1.5  Deg: 77:2114:83:749T_00052737

Query:          1 TGACAGAAGAGAGTGAGCAC
 21
                  |||||||||||||*||||||*
Sbjct:       2122 ACTGTCTTCTCTCTCTCGTGC 2102
                        | ||||||| |||--------------------------------1
                        | ||||||| ||---------------------------------1
                        | ||||||| |----------------------------------1
                        | |||||||------------------------------------1
                        | ||||||-------------------------------------1
                        | |||||--------------------------------------1
                        | ||||---------------------------------------1
                        | |||----------------------------------------9
                        | ||-----------------------------------------56
                        | |------------------------------------------77
                        |--------------------------------------------1
```

```
>miR156d-5p
    Score: 1.5  Deg: 5:2012:5:54   T_00071663

Query:          1 TGACAGAAGAGAGTGAGCAC
 21
                  |||||||||||||*||||||*
Sbjct:       2021 ACTGTCTTCTCTCTCTCGTGT 2001
                      |   ||     | |  |------------------------------1
                      |   ||     | |---------------------------------1
                      |   ||     |-----------------------------------1
                      |   ||-----------------------------------------5
```

```
                     |   |----------------------------------------4
                     |--------------------------------------------1

>miR156d-5p
    Score: 1.5  Deg: 5:1901:5:45   T_00071657

Query:          1 TGACAGAAGAGAGTGAGCAC
 21
                  ||||||||||||*|||||*
Sbjct:       1910 ACTGTCTTCTCTCTCTCGTGT 1890
                     |   ||     | |  |------------------------------1
                     |   ||     | |----------------------------------1
                     |   ||     |------------------------------------1
                     |   ||---------------------------------------5
                     |   |----------------------------------------4
                     |--------------------------------------------1

>miR156d-5p
    Score: 1.5  Deg: 5:1222:5:44   T_00071664

Query:          1 TGACAGAAGAGAGTGAGCAC
 21
                  ||||||||||||*|||||*
Sbjct:       1231 ACTGTCTTCTCTCTCTCGTGT 1211
                     |   ||     | |  |------------------------------1
                     |   ||     | |----------------------------------1
                     |   ||     |------------------------------------1
                     |   ||---------------------------------------5
                     |   |----------------------------------------4
                     |--------------------------------------------1

>miR156d-5p
    Score: 1.5  Deg: 5:2528:631:10547  T_00071658

Query:          1 TGACAGAAGAGAGTGAGCAC
 21
                  ||||||||||||*|||||*
Sbjct:       2537 ACTGTCTTCTCTCTCTCGTGT 2517
                     |   ||     | |  |------------------------------1
                     |   ||     | |----------------------------------1
                     |   ||     |------------------------------------1
                     |   ||---------------------------------------5
                     |   |----------------------------------------4
                     |--------------------------------------------1
```

```
>miR156d-5p
    Score: 1.5  Deg: 5:1457:5:53   T_00071665

Query:          1 TGACAGAAGAGAGTGAGCAC
 21
                  |||||||||||||*||||||*
Sbjct:       1466 ACTGTCTTCTCTCTCTCGTGT 1446
                      |   ||     | |  |-------------------------------1
                      |   ||     | |----------------------------------1
                      |   ||     |------------------------------------1
                      |   ||------------------------------------------5
                      |   |-------------------------------------------4
                      |-----------------------------------------------1

>miR156d-5p
    Score: 1.5  Deg: 5:2084:631:10538  T_00071656

Query:          1 TGACAGAAGAGAGTGAGCAC
 21
                  |||||||||||||*||||||*
Sbjct:       2093 ACTGTCTTCTCTCTCTCGTGT 2073
                      |   ||     | |  |-------------------------------1
                      |   ||     | |----------------------------------1
                      |   ||     |------------------------------------1
                      |   ||------------------------------------------5
                      |   |-------------------------------------------4
                      |-----------------------------------------------1

>miR156d-5p
    Score: 1.5  Deg: 5:1826:5:53   T_00071661

Query:          1 TGACAGAAGAGAGTGAGCAC
 21
                  |||||||||||||*||||||*
Sbjct:       1835 ACTGTCTTCTCTCTCTCGTGT 1815
                      |   ||     | |  |-------------------------------1
                      |   ||     | |----------------------------------1
                      |   ||     |------------------------------------1
                      |   ||------------------------------------------5
                      |   |-------------------------------------------4
                      |-----------------------------------------------1

>miR156d-5p
    Score: 1.5  Deg: 5:1160:5:44   T_00071667
```

```
Query:          1 TGACAGAAGAGAGTGAGCAC
 21
                  |||||||||||||*||||||*
Sbjct:       1169 ACTGTCTTCTCTCTCTCGTGT 1149
                      |   ||      | |  |-------------------------------1
                      |   ||      | |---------------------------------1
                      |   ||      |-----------------------------------1
                      |   ||-----------------------------------------5
                      |   |------------------------------------------4
                      |-----------------------------------------------1

>miR156d-5p
    Score: 1.5  Deg: 5:2345:5:54   T_00071659

Query:          1 TGACAGAAGAGAGTGAGCAC
 21
                  |||||||||||||*||||||*
Sbjct:       2354 ACTGTCTTCTCTCTCTCGTGT 2334
                      |   ||      | |  |-------------------------------1
                      |   ||      | |---------------------------------1
                      |   ||      |-----------------------------------1
                      |   ||-----------------------------------------5
                      |   |------------------------------------------4
                      |-----------------------------------------------1

>miR156d-5p
    Score: 1.5  Deg: 5:1931:631:10538  T_00071660

Query:          1 TGACAGAAGAGAGTGAGCAC
 21
                  |||||||||||||*||||||*
Sbjct:       1940 ACTGTCTTCTCTCTCTCGTGT 1920
                      |   ||      | |  |-------------------------------1
                      |   ||      | |---------------------------------1
                      |   ||      |-----------------------------------1
                      |   ||-----------------------------------------5
                      |   |------------------------------------------4
                      |-----------------------------------------------1

>miR156d-5p
    Score: 1.5  Deg: 5:2217:631:10547  T_00071662

Query:          1 TGACAGAAGAGAGTGAGCAC
 21
                  |||||||||||||*||||||*
```

```
Sbjct:       2226 ACTGTCTTCTCTCTCTCGTGT 2206
                      |   ||     | |  |-------------------------------1
                      |   ||     | |----------------------------------1
                      |   ||     |------------------------------------1
                      |   ||------------------------------------------5
                      |   |-------------------------------------------4
                      |-----------------------------------------------1

>miR156d-5p
    Score: 1.5  Deg: 5:1161:5:44   T_00071666

Query:          1 TGACAGAAGAGAGTGAGCAC
 21
                  ||||||||||||*||||||*
Sbjct:       1170 ACTGTCTTCTCTCTCTCGTGT 1150
                      |   ||     | |  |-------------------------------1
                      |   ||     | |----------------------------------1
                      |   ||     |------------------------------------1
                      |   ||------------------------------------------5
                      |   |-------------------------------------------4
                      |-----------------------------------------------1


>miR156e-5p
    Score: 0.8  Deg: 10:1311:27:223T_00091783

Query:          1 TTGACAGAAGAGAGAGAGCAC
 22
                  :||||||||||||||||||||*
Sbjct:       1320 GACTGTCTTCTCTCTCTCGTGT 1299
                          ||||    |    |-------------------------------1
                          ||||    |-----------------------------------2
                          ||||-----------------------------------------6
                          |||------------------------------------------6
                          ||-------------------------------------------10
                          |--------------------------------------------1

>miR156e-5p
    Score: 0.8  Deg: 10:1668:27:226T_00091779

Query:          1 TTGACAGAAGAGAGAGAGCAC
 22
                  :||||||||||||||||||||*
Sbjct:       1677 GACTGTCTTCTCTCTCTCGTGT 1656
                          ||||    |    |-------------------------------1
```

```
                          ||||    |-----------------------------------2
                          ||||----------------------------------------6
                          |||-----------------------------------------6
                          ||------------------------------------------10
                          |-------------------------------------------1

>miR156e-5p
    Score: 0.8  Deg: 10:2823:27:234T_00091781

Query:          1 TTGACAGAAGAGAGAGAGCAC
 22
                  :||||||||||||||||||||*
Sbjct:       2832 GACTGTCTTCTCTCTCTCGTGT 2811
                          ||||    |    |------------------------------1
                          ||||    |-----------------------------------2
                          ||||----------------------------------------6
                          |||-----------------------------------------6
                          ||------------------------------------------10
                          |-------------------------------------------1

>miR156e-5p
    Score: 0.8  Deg: 10:1470:27:223T_00091782

Query:          1 TTGACAGAAGAGAGAGAGCAC
 22
                  :||||||||||||||||||||*
Sbjct:       1479 GACTGTCTTCTCTCTCTCGTGT 1458
                          ||||    |    |------------------------------1
                          ||||    |-----------------------------------2
                          ||||----------------------------------------6
                          |||-----------------------------------------6
                          ||------------------------------------------10
                          |-------------------------------------------1

>miR156e-5p
    Score: 0.8  Deg: 10:1199:27:223T_00091784

Query:          1 TTGACAGAAGAGAGAGAGCAC
 22
                  :||||||||||||||||||||*
Sbjct:       1208 GACTGTCTTCTCTCTCTCGTGT 1187
                          ||||    |    |------------------------------1
                          ||||    |-----------------------------------2
                          ||||----------------------------------------6
                          |||-----------------------------------------6
```

```
                     ||-----------------------------------------10
                     |------------------------------------------1

>miR156e-5p
   Score: 0.8  Deg: 10:1496:27:226T_00091780

Query:         1 TTGACAGAAGAGAGAGAGCAC
 22
                 :||||||||||||||||||||*
Sbjct:      1505 GACTGTCTTCTCTCTCTCGTGT 1484
                         ||||    |    |-------------------------1
                         ||||    |------------------------------2
                         ||||-----------------------------------6
                         |||------------------------------------6
                         ||-------------------------------------10
                         |--------------------------------------1

>miR156e-5p
   Score: 1.5  Deg: 22:1017:24:363T_00090332

Query:         1 TTGACAGAAGAGAGAGAGCAC
 22
                 ||||||||||*||||||||*
Sbjct:      1026 AACTGTCTTCTATCTCTCGTGC 1005
                      | | ||||||  |----------------------------2
                      | | ||||||---------------------------------1
                      | | |||||----------------------------------1
                      | | ||||-----------------------------------1
                      | | |||------------------------------------2
                      | | ||-------------------------------------3
                      | | |--------------------------------------22
                      | |----------------------------------------1
                      |------------------------------------------2

>miR156e-5p
   Score: 1.5  Deg: 22:1088:24:371T_00090336

Query:         1 TTGACAGAAGAGAGAGAGCAC
 22
                 ||||||||||*||||||||*
Sbjct:      1097 AACTGTCTTCTATCTCTCGTGC 1076
                      | | ||||||  |----------------------------2
                      | | ||||||---------------------------------1
                      | | |||||----------------------------------1
                      | | ||||-----------------------------------1
```

```
                       | | |||-----------------------------------------2
                       | | ||------------------------------------------3
                       | | |-------------------------------------------22
                       | |---------------------------------------------1
                       |-----------------------------------------------2

>miR156e-5p
    Score: 1.5  Deg: 22:1171:24:378T_00090333

Query:          1 TTGACAGAAGAGAGAGAGCAC
 22
                  |||||||||||*|||||||||*
Sbjct:       1180 AACTGTCTTCTATCTCTCGTGC 1159
                       | | ||||||  |-----------------------------------2
                       | | ||||||--------------------------------------1
                       | | |||||---------------------------------------1
                       | | ||||----------------------------------------1
                       | | |||-----------------------------------------2
                       | | ||------------------------------------------3
                       | | |-------------------------------------------22
                       | |---------------------------------------------1
                       |-----------------------------------------------2

>miR156e-5p
    Score: 1.5  Deg: 22:1129:24:363T_00090331

Query:          1 TTGACAGAAGAGAGAGAGCAC
 22
                  |||||||||||*|||||||||*
Sbjct:       1138 AACTGTCTTCTATCTCTCGTGC 1117
                       | | ||||||  |-----------------------------------2
                       | | ||||||--------------------------------------1
                       | | |||||---------------------------------------1
                       | | ||||----------------------------------------1
                       | | |||-----------------------------------------2
                       | | ||------------------------------------------3
                       | | |-------------------------------------------22
                       | |---------------------------------------------1
                       |-----------------------------------------------2

>miR156e-5p
    Score: 1.5  Deg: 22:958:24:363 T_00090335

Query:          1 TTGACAGAAGAGAGAGAGCAC
 22
```

```
                  |||||||||||*|||||||||*
Sbjct:        967 AACTGTCTTCTATCTCTCGTGC 946
                       | | ||||||  |-------------------------------------2
                       | | ||||||----------------------------------------1
                       | | |||||-----------------------------------------1
                       | | ||||------------------------------------------1
                       | | |||-------------------------------------------2
                       | | ||--------------------------------------------3
                       | | |---------------------------------------------22
                       | |-----------------------------------------------1
                       |-------------------------------------------------2
```

>miR156e-5p
    Score: 1.5  Deg: 22:941:24:361 T_00090334

```
Query:          1 TTGACAGAAGAGAGAGAGCAC
 22
                  |||||||||||*|||||||||*
Sbjct:        950 AACTGTCTTCTATCTCTCGTGC 929
                       | | ||||||  |-------------------------------------2
                       | | ||||||----------------------------------------1
                       | | |||||-----------------------------------------1
                       | | ||||------------------------------------------1
                       | | |||-------------------------------------------2
                       | | ||--------------------------------------------3
                       | | |---------------------------------------------22
                       | |-----------------------------------------------1
                       |-------------------------------------------------2
```

>miR156e-5p
    Score: 1.5  Deg: 79:1218:79:519T_00059648

```
Query:          1 TTGACAGAAGAGAGAGAGCAC
 22
                  *|||||||||||||||||||**
Sbjct:       1227 TACTGTCTTCTCTCTCTCGTAC 1206
                   ||   ||||||    |||------------------------------------1
                   ||   ||||||    ||-------------------------------------3
                   ||   ||||||    |--------------------------------------2
                   ||   ||||||-------------------------------------------2
                   ||   |||||--------------------------------------------50
                   ||   ||||---------------------------------------------79
                   ||   |||----------------------------------------------3
                   ||   ||-----------------------------------------------3
                   ||   |------------------------------------------------1
```

```
                   ||---------------------------------------------------1
                   |----------------------------------------------------1

>miR156e-5p
    Score: 0.5  Deg: 9:1100:9:46   T_00080616

Query:          1 TTGACAGAAGAGAGAGAGCAC
 22
                  ||||||||||||||||||||*
Sbjct:       1110 AACTGTCTTCTCTCTCTCGTGT 1089
                           ||   |    | |--------------------------------1
                           ||   |    |----------------------------------1
                           ||   |---------------------------------------6
                           ||-------------------------------------------9
                           |--------------------------------------------4

>miR156e-5p
    Score: 0.5  Deg: 9:1250:9:33   T_00080613

Query:          1 TTGACAGAAGAGAGAGAGCAC
 22
                  ||||||||||||||||||||*
Sbjct:       1260 AACTGTCTTCTCTCTCTCGTGT 1239
                           ||   |    | |--------------------------------1
                           ||   |    |----------------------------------1
                           ||   |---------------------------------------6
                           ||-------------------------------------------9
                           |--------------------------------------------4

>miR156e-5p
    Score: 0.5  Deg: 9:1195:9:64   T_00080614

Query:          1 TTGACAGAAGAGAGAGAGCAC
 22
                  ||||||||||||||||||||*
Sbjct:       1205 AACTGTCTTCTCTCTCTCGTGT 1184
                           ||   |    | |--------------------------------1
                           ||   |    |----------------------------------1
                           ||   |---------------------------------------6
                           ||-------------------------------------------9
                           |--------------------------------------------4

>miR156e-5p
    Score: 0.5  Deg: 9:1124:9:46   T_00080615
```

```
Query:          1 TTGACAGAAGAGAGAGAGCAC
 22
                  ||||||||||||||||||||*
Sbjct:       1134 AACTGTCTTCTCTCTCTCGTGT 1113
                           ||   |    | |-------------------------------1
                           ||   |    |---------------------------------1
                           ||   |--------------------------------------6
                           ||------------------------------------------9
                           |-------------------------------------------4

>miR156e-5p
    Score: 0.5  Deg: 9:1250:9:64   T_00080612

Query:          1 TTGACAGAAGAGAGAGAGCAC
 22
                  ||||||||||||||||||||*
Sbjct:       1260 AACTGTCTTCTCTCTCTCGTGT 1239
                           ||   |    | |-------------------------------1
                           ||   |    |---------------------------------1
                           ||   |--------------------------------------6
                           ||------------------------------------------9
                           |-------------------------------------------4

>miR156e-5p
    Score: 1.0  Deg: 12:1418:12:64 T_00046313

Query:          1 TTGACAGAAGAGAGAGAGCAC
 22
                  *|||||||||||||||||||*
Sbjct:       1428 TACTGTCTTCTCTCTCTCGTGT 1407
                       |   |||  |   |  |-------------------------------1
                       |   |||  |   |----------------------------------1
                       |   |||  |--------------------------------------4
                       |   |||-----------------------------------------1
                       |   ||------------------------------------------12
                       |   |-------------------------------------------10
                       |-----------------------------------------------2


>miR156e-5p
    Score: 0.5  Deg: 77:2106:83:749T_00052738

Query:          1 TTGACAGAAGAGAGAGAGCAC
 22
                  ||||||||||||||||||||*
```

```
Sbjct:       2115 AACTGTCTTCTCTCTCTCGTGC 2094
                         | ||||||| |||---------------------------------1
                         | ||||||| ||----------------------------------1
                         | ||||||| |-----------------------------------1
                         | |||||||-------------------------------------1
                         | ||||||--------------------------------------1
                         | |||||---------------------------------------1
                         | ||||----------------------------------------1
                         | |||-----------------------------------------9
                         | ||------------------------------------------56
                         | |-------------------------------------------77
                         |---------------------------------------------1

>miR156e-5p
    Score: 0.5  Deg: 77:443:77:327 T_00052739

Query:          1 TTGACAGAAGAGAGAGAGCAC
 22
                  ||||||||||||||||||||*
Sbjct:        452 AACTGTCTTCTCTCTCTCGTGC 431
                         | ||||||| |||---------------------------------1
                         | ||||||| ||----------------------------------1
                         | ||||||| |-----------------------------------1
                         | |||||||-------------------------------------1
                         | ||||||--------------------------------------1
                         | |||||---------------------------------------1
                         | ||||----------------------------------------1
                         | |||-----------------------------------------9
                         | ||------------------------------------------56
                         | |-------------------------------------------77
                         |---------------------------------------------1

>miR156e-5p
    Score: 0.5  Deg: 77:2114:83:749T_00052737

Query:          1 TTGACAGAAGAGAGAGAGCAC
 22
                  ||||||||||||||||||||*
Sbjct:       2123 AACTGTCTTCTCTCTCTCGTGC 2102
                         | ||||||| |||---------------------------------1
                         | ||||||| ||----------------------------------1
                         | ||||||| |-----------------------------------1
                         | |||||||-------------------------------------1
                         | ||||||--------------------------------------1
                         | |||||---------------------------------------1
```

```
                       | ||||----------------------------------------1
                       | |||-----------------------------------------9
                       | ||------------------------------------------56
                       | |-------------------------------------------77
                       |---------------------------------------------1

>miR156e-5p
    Score: 1.0  Deg: 5:2012:5:54   T_00071663

Query:          1 TTGACAGAAGAGAGAGAGCAC
 22
                  *|||||||||||||||||||*
Sbjct:       2022 TACTGTCTTCTCTCTCTCGTGT 2001
                       |   ||     | |  |-------------------------------1
                       |   ||     | |----------------------------------1
                       |   ||     |------------------------------------1
                       |   ||------------------------------------------5
                       |   |-------------------------------------------4
                       |-----------------------------------------------1

>miR156e-5p
    Score: 1.0  Deg: 5:1901:5:45   T_00071657

Query:          1 TTGACAGAAGAGAGAGAGCAC
 22
                  *|||||||||||||||||||*
Sbjct:       1911 TACTGTCTTCTCTCTCTCGTGT 1890
                       |   ||     | |  |-------------------------------1
                       |   ||     | |----------------------------------1
                       |   ||     |------------------------------------1
                       |   ||------------------------------------------5
                       |   |-------------------------------------------4
                       |-----------------------------------------------1

>miR156e-5p
    Score: 1.0  Deg: 5:1222:5:44   T_00071664

Query:          1 TTGACAGAAGAGAGAGAGCAC
 22
                  *|||||||||||||||||||*
Sbjct:       1232 TACTGTCTTCTCTCTCTCGTGT 1211
                       |   ||     | |  |-------------------------------1
                       |   ||     | |----------------------------------1
                       |   ||     |------------------------------------1
                       |   ||------------------------------------------5
```

```
                        |   |-------------------------------------------4
                        |-----------------------------------------------1

>miR156e-5p
    Score: 1.0  Deg: 5:2528:631:10547  T_00071658

Query:           1 TTGACAGAAGAGAGAGAGCAC
 22
                  *||||||||||||||||||||*
Sbjct:       2538 TACTGTCTTCTCTCTCTCGTGT 2517
                        |   ||     | |  |-------------------------------1
                        |   ||     | |----------------------------------1
                        |   ||     |------------------------------------1
                        |   ||------------------------------------------5
                        |   |-------------------------------------------4
                        |-----------------------------------------------1

>miR156e-5p
    Score: 1.0  Deg: 5:1457:5:53   T_00071665

Query:           1 TTGACAGAAGAGAGAGAGCAC
 22
                  *||||||||||||||||||||*
Sbjct:       1467 TACTGTCTTCTCTCTCTCGTGT 1446
                        |   ||     | |  |-------------------------------1
                        |   ||     | |----------------------------------1
                        |   ||     |------------------------------------1
                        |   ||------------------------------------------5
                        |   |-------------------------------------------4
                        |-----------------------------------------------1

>miR156e-5p
    Score: 1.0  Deg: 5:2084:631:10538  T_00071656

Query:           1 TTGACAGAAGAGAGAGAGCAC
 22
                  *||||||||||||||||||||*
Sbjct:       2094 TACTGTCTTCTCTCTCTCGTGT 2073
                        |   ||     | |  |-------------------------------1
                        |   ||     | |----------------------------------1
                        |   ||     |------------------------------------1
                        |   ||------------------------------------------5
                        |   |-------------------------------------------4
                        |-----------------------------------------------1
```

```
>miR156e-5p
    Score: 1.0  Deg: 5:1826:5:53   T_00071661

Query:          1 TTGACAGAAGAGAGAGAGCAC
 22
                  *|||||||||||||||||||*
Sbjct:       1836 TACTGTCTTCTCTCTCTCGTGT 1815
                       |   ||      | |  |------------------------------1
                       |   ||      | |----------------------------------1
                       |   ||      |------------------------------------1
                       |   ||------------------------------------------5
                       |   |-------------------------------------------4
                       |-----------------------------------------------1

>miR156e-5p
    Score: 1.0  Deg: 5:1160:5:44   T_00071667

Query:          1 TTGACAGAAGAGAGAGAGCAC
 22
                  *|||||||||||||||||||*
Sbjct:       1170 TACTGTCTTCTCTCTCTCGTGT 1149
                       |   ||      | |  |------------------------------1
                       |   ||      | |----------------------------------1
                       |   ||      |------------------------------------1
                       |   ||------------------------------------------5
                       |   |-------------------------------------------4
                       |-----------------------------------------------1

>miR156e-5p
    Score: 1.0  Deg: 5:2345:5:54   T_00071659

Query:          1 TTGACAGAAGAGAGAGAGCAC
 22
                  *|||||||||||||||||||*
Sbjct:       2355 TACTGTCTTCTCTCTCTCGTGT 2334
                       |   ||      | |  |------------------------------1
                       |   ||      | |----------------------------------1
                       |   ||      |------------------------------------1
                       |   ||------------------------------------------5
                       |   |-------------------------------------------4
                       |-----------------------------------------------1

>miR156e-5p
    Score: 1.0  Deg: 5:1931:631:10538  T_00071660
```

```
Query:          1 TTGACAGAAGAGAGAGAGCAC
 22
                  *||||||||||||||||||||*
Sbjct:       1941 TACTGTCTTCTCTCTCTCGTGT 1920
                       |   ||     | |  |-------------------------------1
                       |   ||     | |----------------------------------1
                       |   ||     |------------------------------------1
                       |   ||-----------------------------------------5
                       |   |------------------------------------------4
                       |----------------------------------------------1

>miR156e-5p
    Score: 1.0  Deg: 5:2217:631:10547  T_00071662

Query:          1 TTGACAGAAGAGAGAGAGCAC
 22
                  *||||||||||||||||||||*
Sbjct:       2227 TACTGTCTTCTCTCTCTCGTGT 2206
                       |   ||     | |  |-------------------------------1
                       |   ||     | |----------------------------------1
                       |   ||     |------------------------------------1
                       |   ||-----------------------------------------5
                       |   |------------------------------------------4
                       |----------------------------------------------1

>miR156e-5p
    Score: 1.0  Deg: 5:1161:5:44   T_00071666

Query:          1 TTGACAGAAGAGAGAGAGCAC
 22
                  *||||||||||||||||||||*
Sbjct:       1171 TACTGTCTTCTCTCTCTCGTGT 1150
                       |   ||     | |  |-------------------------------1
                       |   ||     | |----------------------------------1
                       |   ||     |------------------------------------1
                       |   ||-----------------------------------------5
                       |   |------------------------------------------4
                       |----------------------------------------------1

>miR156f-5p
    Score: 1.5  Deg: 10:1311:27:223T_00091783

Query:          1 TGACAGAAGAGAGTGAGCAC
 21
                  |||||||||||||*||||||*
```

```
Sbjct:       1319 ACTGTCTTCTCTCTCTCGTGT 1299
                         ||||    |    |------------------------------1
                         ||||    |-----------------------------------2
                         ||||-----------------------------------------6
                         |||------------------------------------------6
                         ||-------------------------------------------10
                         |--------------------------------------------1

>miR156f-5p
    Score: 1.5  Deg: 10:1668:27:226T_00091779

Query:          1 TGACAGAAGAGAGTGAGCAC
 21
                  ||||||||||||*||||||*
Sbjct:       1676 ACTGTCTTCTCTCTCTCGTGT 1656
                         ||||    |    |------------------------------1
                         ||||    |-----------------------------------2
                         ||||-----------------------------------------6
                         |||------------------------------------------6
                         ||-------------------------------------------10
                         |--------------------------------------------1

>miR156f-5p
    Score: 1.5  Deg: 10:2823:27:234T_00091781

Query:          1 TGACAGAAGAGAGTGAGCAC
 21
                  ||||||||||||*||||||*
Sbjct:       2831 ACTGTCTTCTCTCTCTCGTGT 2811
                         ||||    |    |------------------------------1
                         ||||    |-----------------------------------2
                         ||||-----------------------------------------6
                         |||------------------------------------------6
                         ||-------------------------------------------10
                         |--------------------------------------------1

>miR156f-5p
    Score: 1.5  Deg: 10:1470:27:223T_00091782

Query:          1 TGACAGAAGAGAGTGAGCAC
 21
                  ||||||||||||*||||||*
Sbjct:       1478 ACTGTCTTCTCTCTCTCGTGT 1458
                         ||||    |    |------------------------------1
                         ||||    |-----------------------------------2
```

```
                         ||||-----------------------------------------6
                         |||------------------------------------------6
                         ||-------------------------------------------10
                         |--------------------------------------------1

>miR156f-5p
    Score: 1.5  Deg: 10:1199:27:223T_00091784

Query:          1 TGACAGAAGAGAGTGAGCAC
 21
                  |||||||||||||*||||||*
Sbjct:       1207 ACTGTCTTCTCTCTCTCGTGT 1187
                         ||||    |    |-------------------------------1
                         ||||    |------------------------------------2
                         ||||-----------------------------------------6
                         |||------------------------------------------6
                         ||-------------------------------------------10
                         |--------------------------------------------1

>miR156f-5p
    Score: 1.5  Deg: 10:1496:27:226T_00091780

Query:          1 TGACAGAAGAGAGTGAGCAC
 21
                  |||||||||||||*||||||*
Sbjct:       1504 ACTGTCTTCTCTCTCTCGTGT 1484
                         ||||    |    |-------------------------------1
                         ||||    |------------------------------------2
                         ||||-----------------------------------------6
                         |||------------------------------------------6
                         ||-------------------------------------------10
                         |--------------------------------------------1

>miR156f-5p
    Score: 2.5  Deg: 22:1017:24:363T_00090332

Query:          1 TGACAGAAGAGAGTGAGCAC
 21
                  ||||||||||*||*||||||*
Sbjct:       1025 ACTGTCTTCTATCTCTCGTGC 1005
                      | | ||||||  |-----------------------------------2
                      | | ||||||--------------------------------------1
                      | | |||||---------------------------------------1
                      | | ||||----------------------------------------1
                      | | |||-----------------------------------------2
```

```
                    | | ||------------------------------------------3
                    | | |-------------------------------------------22
                    | |---------------------------------------------1
                    |-----------------------------------------------2

>miR156f-5p
    Score: 2.5  Deg: 22:1088:24:371T_00090336

Query:          1 TGACAGAAGAGAGTGAGCAC
 21
                  ||||||||||*||*||||||*
Sbjct:       1096 ACTGTCTTCTATCTCTCGTGC 1076
                    | | ||||||  |-----------------------------------2
                    | | ||||||--------------------------------------1
                    | | |||||---------------------------------------1
                    | | ||||----------------------------------------1
                    | | |||-----------------------------------------2
                    | | ||------------------------------------------3
                    | | |-------------------------------------------22
                    | |---------------------------------------------1
                    |-----------------------------------------------2

>miR156f-5p
    Score: 2.5  Deg: 22:1171:24:378T_00090333

Query:          1 TGACAGAAGAGAGTGAGCAC
 21
                  ||||||||||*||*||||||*
Sbjct:       1179 ACTGTCTTCTATCTCTCGTGC 1159
                    | | ||||||  |-----------------------------------2
                    | | ||||||--------------------------------------1
                    | | |||||---------------------------------------1
                    | | ||||----------------------------------------1
                    | | |||-----------------------------------------2
                    | | ||------------------------------------------3
                    | | |-------------------------------------------22
                    | |---------------------------------------------1
                    |-----------------------------------------------2

>miR156f-5p
    Score: 2.5  Deg: 22:1129:24:363T_00090331

Query:          1 TGACAGAAGAGAGTGAGCAC
 21
                  ||||||||||*||*||||||*
```

```
Sbjct:       1137 ACTGTCTTCTATCTCTCGTGC 1117
                      | | ||||||  |-----------------------------------2
                      | | ||||||--------------------------------------1
                      | | |||||---------------------------------------1
                      | | ||||----------------------------------------1
                      | | |||-----------------------------------------2
                      | | ||------------------------------------------3
                      | | |-------------------------------------------22
                      | |---------------------------------------------1
                      |-----------------------------------------------2

>miR156f-5p
    Score: 2.5  Deg: 22:958:24:363 T_00090335

Query:          1 TGACAGAAGAGAGTGAGCAC
 21
                  ||||||||||*||*||||||*
Sbjct:        966 ACTGTCTTCTATCTCTCGTGC 946
                      | | ||||||  |-----------------------------------2
                      | | ||||||--------------------------------------1
                      | | |||||---------------------------------------1
                      | | ||||----------------------------------------1
                      | | |||-----------------------------------------2
                      | | ||------------------------------------------3
                      | | |-------------------------------------------22
                      | |---------------------------------------------1
                      |-----------------------------------------------2

>miR156f-5p
    Score: 2.5  Deg: 22:941:24:361 T_00090334

Query:          1 TGACAGAAGAGAGTGAGCAC
 21
                  ||||||||||*||*||||||*
Sbjct:        949 ACTGTCTTCTATCTCTCGTGC 929
                      | | ||||||  |-----------------------------------2
                      | | ||||||--------------------------------------1
                      | | |||||---------------------------------------1
                      | | ||||----------------------------------------1
                      | | |||-----------------------------------------2
                      | | ||------------------------------------------3
                      | | |-------------------------------------------22
                      | |---------------------------------------------1
                      |-----------------------------------------------2
```

```
>miR156f-5p
    Score: 2.0  Deg: 79:1218:79:519T_00059648

Query:          1 TGACAGAAGAGAGTGAGCAC
 21
                  |||||||||||||*|||||**
Sbjct:       1226 ACTGTCTTCTCTCTCTCGTAC 1206
                  ||   ||||||    |||----------------------------------1
                  ||   ||||||    ||-----------------------------------3
                  ||   ||||||    |------------------------------------2
                  ||   ||||||-----------------------------------------2
                  ||   |||||------------------------------------------50
                  ||   ||||-------------------------------------------79
                  ||   |||--------------------------------------------3
                  ||   ||---------------------------------------------3
                  ||   |----------------------------------------------1
                  ||--------------------------------------------------1
                  |---------------------------------------------------1

>miR156f-5p
    Score: 1.5  Deg: 9:1100:9:46   T_00080616

Query:          1 TGACAGAAGAGAGTGAGCAC
 21
                  |||||||||||||*||||||*
Sbjct:       1109 ACTGTCTTCTCTCTCTCGTGT 1089
                          ||   |    | |-------------------------------1
                          ||   |    |---------------------------------1
                          ||   |--------------------------------------6
                          ||------------------------------------------9
                          |-------------------------------------------4

>miR156f-5p
    Score: 1.5  Deg: 9:1250:9:33   T_00080613

Query:          1 TGACAGAAGAGAGTGAGCAC
 21
                  |||||||||||||*||||||*
Sbjct:       1259 ACTGTCTTCTCTCTCTCGTGT 1239
                          ||   |    | |-------------------------------1
                          ||   |    |---------------------------------1
                          ||   |--------------------------------------6
                          ||------------------------------------------9
                          |-------------------------------------------4
```

```
>miR156f-5p
    Score: 1.5  Deg: 9:1195:9:64   T_00080614

Query:          1 TGACAGAAGAGAGTGAGCAC
 21
                  ||||||||||||*||||||*
Sbjct:       1204 ACTGTCTTCTCTCTCTCGTGT 1184
                          ||   |    | |-------------------------------1
                          ||   |    |---------------------------------1
                          ||   |------------------------------------6
                          ||-------------------------------------------9
                          |--------------------------------------------4

>miR156f-5p
    Score: 1.5  Deg: 9:1124:9:46   T_00080615

Query:          1 TGACAGAAGAGAGTGAGCAC
 21
                  ||||||||||||*||||||*
Sbjct:       1133 ACTGTCTTCTCTCTCTCGTGT 1113
                          ||   |    | |-------------------------------1
                          ||   |    |---------------------------------1
                          ||   |------------------------------------6
                          ||-------------------------------------------9
                          |--------------------------------------------4

>miR156f-5p
    Score: 1.5  Deg: 9:1250:9:64   T_00080612

Query:          1 TGACAGAAGAGAGTGAGCAC
 21
                  ||||||||||||*||||||*
Sbjct:       1259 ACTGTCTTCTCTCTCTCGTGT 1239
                          ||   |    | |-------------------------------1
                          ||   |    |---------------------------------1
                          ||   |------------------------------------6
                          ||-------------------------------------------9
                          |--------------------------------------------4

>miR156f-5p
    Score: 1.5  Deg: 12:1418:12:64 T_00046313

Query:          1 TGACAGAAGAGAGTGAGCAC
 21
                  ||||||||||||*||||||*
```

```
Sbjct:       1427 ACTGTCTTCTCTCTCTCGTGT 1407
                           |    |||  |    |  |------------------------------1
                           |    |||  |    |---------------------------------1
                           |    |||  |--------------------------------------4
                           |    |||-----------------------------------------1
                           |    ||------------------------------------------12
                           |    |-------------------------------------------10
                           |------------------------------------------------2

>miR156f-5p
    Score: 1.5  Deg: 77:2106:83:749T_00052738

Query:          1 TGACAGAAGAGAGTGAGCAC
 21
                  |||||||||||||*||||||*
Sbjct:       2114 ACTGTCTTCTCTCTCTCGTGC 2094
                            | ||||||| |||-------------------------------1
                            | ||||||| ||--------------------------------1
                            | ||||||| |---------------------------------1
                            | |||||||-----------------------------------1
                            | ||||||------------------------------------1
                            | |||||-------------------------------------1
                            | ||||--------------------------------------1
                            | |||---------------------------------------9
                            | ||----------------------------------------56
                            | |-----------------------------------------77
                            |-------------------------------------------1

>miR156f-5p
    Score: 1.5  Deg: 77:443:77:327 T_00052739

Query:          1 TGACAGAAGAGAGTGAGCAC
 21
                  |||||||||||||*||||||*
Sbjct:        451 ACTGTCTTCTCTCTCTCGTGC 431
                            | ||||||| |||-------------------------------1
                            | ||||||| ||--------------------------------1
                            | ||||||| |---------------------------------1
                            | |||||||-----------------------------------1
                            | ||||||------------------------------------1
                            | |||||-------------------------------------1
                            | ||||--------------------------------------1
                            | |||---------------------------------------9
                            | ||----------------------------------------56
                            | |-----------------------------------------77
```

```
                        |--------------------------------------------1

>miR156f-5p
    Score: 1.5  Deg: 77:2114:83:749T_00052737

Query:          1 TGACAGAAGAGAGTGAGCAC
 21
                  ||||||||||||*|||||*
Sbjct:       2122 ACTGTCTTCTCTCTCTCGTGC 2102
                        | ||||||| |||---------------------------------1
                        | ||||||| ||----------------------------------1
                        | ||||||| |-----------------------------------1
                        | |||||||-------------------------------------1
                        | ||||||--------------------------------------1
                        | |||||---------------------------------------1
                        | ||||----------------------------------------1
                        | |||-----------------------------------------9
                        | ||------------------------------------------56
                        | |-------------------------------------------77
                        |--------------------------------------------1

>miR156f-5p
    Score: 1.5  Deg: 5:2012:5:54   T_00071663

Query:          1 TGACAGAAGAGAGTGAGCAC
 21
                  ||||||||||||*|||||*
Sbjct:       2021 ACTGTCTTCTCTCTCTCGTGT 2001
                      |   ||      | |  |-------------------------------1
                      |   ||      | |----------------------------------1
                      |   ||      |------------------------------------1
                      |   ||------------------------------------------5
                      |   |-------------------------------------------4
                      |-----------------------------------------------1

>miR156f-5p
    Score: 1.5  Deg: 5:1901:5:45   T_00071657

Query:          1 TGACAGAAGAGAGTGAGCAC
 21
                  ||||||||||||*|||||*
Sbjct:       1910 ACTGTCTTCTCTCTCTCGTGT 1890
                      |   ||      | |  |-------------------------------1
                      |   ||      | |----------------------------------1
                      |   ||      |------------------------------------1
```

```
                      |   ||------------------------------------------5
                      |   |-------------------------------------------4
                      |-----------------------------------------------1

>miR156f-5p
    Score: 1.5  Deg: 5:1222:5:44   T_00071664

Query:          1 TGACAGAAGAGAGTGAGCAC
 21
                  |||||||||||||*||||||*
Sbjct:       1231 ACTGTCTTCTCTCTCTCGTGT 1211
                      |   ||     | |  |-------------------------------1
                      |   ||     | |----------------------------------1
                      |   ||     |------------------------------------1
                      |   ||------------------------------------------5
                      |   |-------------------------------------------4
                      |-----------------------------------------------1

>miR156f-5p
    Score: 1.5  Deg: 5:2528:631:10547  T_00071658

Query:          1 TGACAGAAGAGAGTGAGCAC
 21
                  |||||||||||||*||||||*
Sbjct:       2537 ACTGTCTTCTCTCTCTCGTGT 2517
                      |   ||     | |  |-------------------------------1
                      |   ||     | |----------------------------------1
                      |   ||     |------------------------------------1
                      |   ||------------------------------------------5
                      |   |-------------------------------------------4
                      |-----------------------------------------------1

>miR156f-5p
    Score: 1.5  Deg: 5:1457:5:53   T_00071665

Query:          1 TGACAGAAGAGAGTGAGCAC
 21
                  |||||||||||||*||||||*
Sbjct:       1466 ACTGTCTTCTCTCTCTCGTGT 1446
                      |   ||     | |  |-------------------------------1
                      |   ||     | |----------------------------------1
                      |   ||     |------------------------------------1
                      |   ||------------------------------------------5
                      |   |-------------------------------------------4
                      |-----------------------------------------------1
```

```
>miR156f-5p
    Score: 1.5  Deg: 5:2084:631:10538  T_00071656

Query:          1 TGACAGAAGAGAGTGAGCAC
 21
                  ||||||||||||*||||||*
Sbjct:       2093 ACTGTCTTCTCTCTCTCGTGT 2073
                      |   ||     | |  |-------------------------------1
                      |   ||     | |----------------------------------1
                      |   ||     |------------------------------------1
                      |   ||------------------------------------------5
                      |   |-------------------------------------------4
                      |-----------------------------------------------1

>miR156f-5p
    Score: 1.5  Deg: 5:1826:5:53   T_00071661

Query:          1 TGACAGAAGAGAGTGAGCAC
 21
                  ||||||||||||*||||||*
Sbjct:       1835 ACTGTCTTCTCTCTCTCGTGT 1815
                      |   ||     | |  |-------------------------------1
                      |   ||     | |----------------------------------1
                      |   ||     |------------------------------------1
                      |   ||------------------------------------------5
                      |   |-------------------------------------------4
                      |-----------------------------------------------1

>miR156f-5p
    Score: 1.5  Deg: 5:1160:5:44   T_00071667

Query:          1 TGACAGAAGAGAGTGAGCAC
 21
                  ||||||||||||*||||||*
Sbjct:       1169 ACTGTCTTCTCTCTCTCGTGT 1149
                      |   ||     | |  |-------------------------------1
                      |   ||     | |----------------------------------1
                      |   ||     |------------------------------------1
                      |   ||------------------------------------------5
                      |   |-------------------------------------------4
                      |-----------------------------------------------1

>miR156f-5p
    Score: 1.5  Deg: 5:2345:5:54   T_00071659
```

```
Query:          1 TGACAGAAGAGAGTGAGCAC
 21
                  |||||||||||||*||||||*
Sbjct:       2354 ACTGTCTTCTCTCTCTCGTGT 2334
                      |   ||     | |  |-------------------------------1
                      |   ||     | |----------------------------------1
                      |   ||     |------------------------------------1
                      |   ||------------------------------------------5
                      |   |-------------------------------------------4
                      |-----------------------------------------------1

>miR156f-5p
    Score: 1.5  Deg: 5:1931:631:10538  T_00071660

Query:          1 TGACAGAAGAGAGTGAGCAC
 21
                  |||||||||||||*||||||*
Sbjct:       1940 ACTGTCTTCTCTCTCTCGTGT 1920
                      |   ||     | |  |-------------------------------1
                      |   ||     | |----------------------------------1
                      |   ||     |------------------------------------1
                      |   ||------------------------------------------5
                      |   |-------------------------------------------4
                      |-----------------------------------------------1

>miR156f-5p
    Score: 1.5  Deg: 5:2217:631:10547  T_00071662

Query:          1 TGACAGAAGAGAGTGAGCAC
 21
                  |||||||||||||*||||||*
Sbjct:       2226 ACTGTCTTCTCTCTCTCGTGT 2206
                      |   ||     | |  |-------------------------------1
                      |   ||     | |----------------------------------1
                      |   ||     |------------------------------------1
                      |   ||------------------------------------------5
                      |   |-------------------------------------------4
                      |-----------------------------------------------1

>miR156f-5p
    Score: 1.5  Deg: 5:1161:5:44   T_00071666

Query:          1 TGACAGAAGAGAGTGAGCAC
 21
```

```
                 |||||||||||||*||||||*
Sbjct:       1170 ACTGTCTTCTCTCTCTCGTGT 1150
                     |   ||     | |  |-------------------------------1
                     |   ||     | |----------------------------------1
                     |   ||     |------------------------------------1
                     |   ||------------------------------------------5
                     |   |-------------------------------------------4
                     |-----------------------------------------------1

>miR156g
    Score: 0.8  Deg: 10:1311:27:223T_00091783

Query:          1 TTGACAGAAGAGAGAGAGCAC
 22
                  :|||||||||||||||||||*
Sbjct:       1320 GACTGTCTTCTCTCTCTCGTGT 1299
                          ||||    |    |-------------------------------1
                          ||||    |------------------------------------2
                          ||||-----------------------------------------6
                          |||------------------------------------------6
                          ||-------------------------------------------10
                          |--------------------------------------------1

>miR156g
    Score: 0.8  Deg: 10:1668:27:226T_00091779

Query:          1 TTGACAGAAGAGAGAGAGCAC
 22
                  :|||||||||||||||||||*
Sbjct:       1677 GACTGTCTTCTCTCTCTCGTGT 1656
                          ||||    |    |-------------------------------1
                          ||||    |------------------------------------2
                          ||||-----------------------------------------6
                          |||------------------------------------------6
                          ||-------------------------------------------10
                          |--------------------------------------------1

>miR156g
    Score: 0.8  Deg: 10:2823:27:234T_00091781

Query:          1 TTGACAGAAGAGAGAGAGCAC
 22
                  :|||||||||||||||||||*
Sbjct:       2832 GACTGTCTTCTCTCTCTCGTGT 2811
                          ||||    |    |-------------------------------1
```

```
                          ||||    |-----------------------------------2
                          ||||---------------------------------------6
                          |||----------------------------------------6
                          ||-----------------------------------------10
                          |------------------------------------------1
```

>miR156g
    Score: 0.8  Deg: 10:1470:27:223T_00091782

```
Query:          1 TTGACAGAAGAGAGAGAGCAC
 22
                  :|||||||||||||||||||*
Sbjct:       1479 GACTGTCTTCTCTCTCTCGTGT 1458
                          ||||    |    |------------------------------1
                          ||||    |-----------------------------------2
                          ||||---------------------------------------6
                          |||----------------------------------------6
                          ||-----------------------------------------10
                          |------------------------------------------1
```

>miR156g
    Score: 0.8  Deg: 10:1199:27:223T_00091784

```
Query:          1 TTGACAGAAGAGAGAGAGCAC
 22
                  :|||||||||||||||||||*
Sbjct:       1208 GACTGTCTTCTCTCTCTCGTGT 1187
                          ||||    |    |------------------------------1
                          ||||    |-----------------------------------2
                          ||||---------------------------------------6
                          |||----------------------------------------6
                          ||-----------------------------------------10
                          |------------------------------------------1
```

>miR156g
    Score: 0.8  Deg: 10:1496:27:226T_00091780

```
Query:          1 TTGACAGAAGAGAGAGAGCAC
 22
                  :|||||||||||||||||||*
Sbjct:       1505 GACTGTCTTCTCTCTCTCGTGT 1484
                          ||||    |    |------------------------------1
                          ||||    |-----------------------------------2
                          ||||---------------------------------------6
                          |||----------------------------------------6
```

```
                        ||-------------------------------------------10
                        |--------------------------------------------1

>miR156g
    Score: 1.5  Deg: 22:1017:24:363T_00090332

Query:           1 TTGACAGAAGAGAGAGAGCAC
 22
                   |||||||||||*|||||||||*
Sbjct:        1026 AACTGTCTTCTATCTCTCGTGC 1005
                        | | ||||||  |-----------------------------------2
                        | | ||||||----------------------------------------1
                        | | |||||-----------------------------------------1
                        | | ||||------------------------------------------1
                        | | |||-------------------------------------------2
                        | | ||--------------------------------------------3
                        | | |---------------------------------------------22
                        | |-----------------------------------------------1
                        |-------------------------------------------------2

>miR156g
    Score: 1.5  Deg: 22:1088:24:371T_00090336

Query:           1 TTGACAGAAGAGAGAGAGCAC
 22
                   |||||||||||*|||||||||*
Sbjct:        1097 AACTGTCTTCTATCTCTCGTGC 1076
                        | | ||||||  |-----------------------------------2
                        | | ||||||----------------------------------------1
                        | | |||||-----------------------------------------1
                        | | ||||------------------------------------------1
                        | | |||-------------------------------------------2
                        | | ||--------------------------------------------3
                        | | |---------------------------------------------22
                        | |-----------------------------------------------1
                        |-------------------------------------------------2

>miR156g
    Score: 1.5  Deg: 22:1171:24:378T_00090333

Query:           1 TTGACAGAAGAGAGAGAGCAC
 22
                   |||||||||||*|||||||||*
Sbjct:        1180 AACTGTCTTCTATCTCTCGTGC 1159
                        | | ||||||  |-----------------------------------2
```

```
                    | | ||||||-------------------------------------1
                    | | |||||--------------------------------------1
                    | | ||||---------------------------------------1
                    | | |||----------------------------------------2
                    | | ||-----------------------------------------3
                    | | |------------------------------------------22
                    | |--------------------------------------------1
                    |----------------------------------------------2

>miR156g
    Score: 1.5  Deg: 22:1129:24:363T_00090331

Query:          1 TTGACAGAAGAGAGAGAGCAC
 22
                  |||||||||||*|||||||||*
Sbjct:       1138 AACTGTCTTCTATCTCTCGTGC 1117
                    | | ||||||  |----------------------------------2
                    | | ||||||-------------------------------------1
                    | | |||||--------------------------------------1
                    | | ||||---------------------------------------1
                    | | |||----------------------------------------2
                    | | ||-----------------------------------------3
                    | | |------------------------------------------22
                    | |--------------------------------------------1
                    |----------------------------------------------2

>miR156g
    Score: 1.5  Deg: 22:958:24:363 T_00090335

Query:          1 TTGACAGAAGAGAGAGAGCAC
 22
                  |||||||||||*|||||||||*
Sbjct:        967 AACTGTCTTCTATCTCTCGTGC 946
                    | | ||||||  |----------------------------------2
                    | | ||||||-------------------------------------1
                    | | |||||--------------------------------------1
                    | | ||||---------------------------------------1
                    | | |||----------------------------------------2
                    | | ||-----------------------------------------3
                    | | |------------------------------------------22
                    | |--------------------------------------------1
                    |----------------------------------------------2

>miR156g
    Score: 1.5  Deg: 22:941:24:361 T_00090334
```

```
Query:          1 TTGACAGAAGAGAGAGAGCAC
 22
                  |||||||||||*|||||||||*
Sbjct:        950 AACTGTCTTCTATCTCTCGTGC 929
                       | | ||||||  |-----------------------------------2
                       | | ||||||--------------------------------------1
                       | | |||||---------------------------------------1
                       | | ||||----------------------------------------1
                       | | |||-----------------------------------------2
                       | | ||------------------------------------------3
                       | | |-------------------------------------------22
                       | |---------------------------------------------1
                       |-----------------------------------------------2

>miR156g
    Score: 1.5  Deg: 79:1218:79:519T_00059648

Query:          1 TTGACAGAAGAGAGAGAGCAC
 22
                  *|||||||||||||||||||**
Sbjct:       1227 TACTGTCTTCTCTCTCTCGTAC 1206
                   ||   ||||||    |||----------------------------------1
                   ||   ||||||    ||-----------------------------------3
                   ||   ||||||    |------------------------------------2
                   ||   ||||||-----------------------------------------2
                   ||   |||||------------------------------------------50
                   ||   ||||-------------------------------------------79
                   ||   |||--------------------------------------------3
                   ||   ||---------------------------------------------3
                   ||   |----------------------------------------------1
                   ||--------------------------------------------------1
                   |---------------------------------------------------1

>miR156g
    Score: 0.5  Deg: 9:1100:9:46   T_00080616

Query:          1 TTGACAGAAGAGAGAGAGCAC
 22
                  |||||||||||||||||||||*
Sbjct:       1110 AACTGTCTTCTCTCTCTCGTGT 1089
                           ||   |    | |-------------------------------1
                           ||   |    |---------------------------------1
                           ||   |--------------------------------------6
                           ||------------------------------------------9
```

```
                           |------------------------------------------4

>miR156g
    Score: 0.5  Deg: 9:1250:9:33   T_00080613

Query:          1 TTGACAGAAGAGAGAGAGCAC
 22
                  ||||||||||||||||||||*
Sbjct:       1260 AACTGTCTTCTCTCTCTCGTGT 1239
                           ||    |    | |------------------------------1
                           ||    |    |--------------------------------1
                           ||    |-------------------------------------6
                           ||-----------------------------------------9
                           |------------------------------------------4

>miR156g
    Score: 0.5  Deg: 9:1195:9:64   T_00080614

Query:          1 TTGACAGAAGAGAGAGAGCAC
 22
                  ||||||||||||||||||||*
Sbjct:       1205 AACTGTCTTCTCTCTCTCGTGT 1184
                           ||    |    | |------------------------------1
                           ||    |    |--------------------------------1
                           ||    |-------------------------------------6
                           ||-----------------------------------------9
                           |------------------------------------------4

>miR156g
    Score: 0.5  Deg: 9:1124:9:46   T_00080615

Query:          1 TTGACAGAAGAGAGAGAGCAC
 22
                  ||||||||||||||||||||*
Sbjct:       1134 AACTGTCTTCTCTCTCTCGTGT 1113
                           ||    |    | |------------------------------1
                           ||    |    |--------------------------------1
                           ||    |-------------------------------------6
                           ||-----------------------------------------9
                           |------------------------------------------4

>miR156g
    Score: 0.5  Deg: 9:1250:9:64   T_00080612

Query:          1 TTGACAGAAGAGAGAGAGCAC
```

```
 22
                  ||||||||||||||||||||*
Sbjct:       1260 AACTGTCTTCTCTCTCTCGTGT 1239
                           ||   |    | |-------------------------------1
                           ||   |    |---------------------------------1
                           ||   |--------------------------------------6
                           ||------------------------------------------9
                           |-------------------------------------------4
```

```
>miR156g
    Score: 1.0  Deg: 12:1418:12:64 T_00046313

Query:          1 TTGACAGAAGAGAGAGAGCAC
 22
                  *|||||||||||||||||||*
Sbjct:       1428 TACTGTCTTCTCTCTCTCGTGT 1407
                       |   |||  |   |  |-------------------------------1
                       |   |||  |   |----------------------------------1
                       |   |||  |--------------------------------------4
                       |   |||-----------------------------------------1
                       |   ||------------------------------------------12
                       |   |-------------------------------------------10
                       |-----------------------------------------------2
```

```
>miR156g
    Score: 0.5  Deg: 77:2106:83:749T_00052738

Query:          1 TTGACAGAAGAGAGAGAGCAC
 22
                  ||||||||||||||||||||*
Sbjct:       2115 AACTGTCTTCTCTCTCTCGTGC 2094
                         | ||||||| |||---------------------------------1
                         | ||||||| ||----------------------------------1
                         | ||||||| |-----------------------------------1
                         | |||||||-------------------------------------1
                         | ||||||--------------------------------------1
                         | |||||---------------------------------------1
                         | ||||----------------------------------------1
                         | |||-----------------------------------------9
                         | ||------------------------------------------56
                         | |-------------------------------------------77
                         |---------------------------------------------1
```

>miR156g

```
    Score: 0.5  Deg: 77:443:77:327 T_00052739

Query:          1 TTGACAGAAGAGAGAGAGCAC
 22
                  |||||||||||||||||||||*
Sbjct:        452 AACTGTCTTCTCTCTCTCGTGC 431
                         | ||||||| |||---------------------------------1
                         | ||||||| ||----------------------------------1
                         | ||||||| |-----------------------------------1
                         | |||||||-------------------------------------1
                         | ||||||--------------------------------------1
                         | |||||---------------------------------------1
                         | ||||----------------------------------------1
                         | |||-----------------------------------------9
                         | ||------------------------------------------56
                         | |-------------------------------------------77
                         |---------------------------------------------1

>miR156g
    Score: 0.5  Deg: 77:2114:83:749T_00052737

Query:          1 TTGACAGAAGAGAGAGAGCAC
 22
                  |||||||||||||||||||||*
Sbjct:       2123 AACTGTCTTCTCTCTCTCGTGC 2102
                         | ||||||| |||---------------------------------1
                         | ||||||| ||----------------------------------1
                         | ||||||| |-----------------------------------1
                         | |||||||-------------------------------------1
                         | ||||||--------------------------------------1
                         | |||||---------------------------------------1
                         | ||||----------------------------------------1
                         | |||-----------------------------------------9
                         | ||------------------------------------------56
                         | |-------------------------------------------77
                         |---------------------------------------------1

>miR156g
    Score: 1.0  Deg: 5:2012:5:54   T_00071663

Query:          1 TTGACAGAAGAGAGAGAGCAC
 22
                  *||||||||||||||||||||*
Sbjct:       2022 TACTGTCTTCTCTCTCTCGTGT 2001
                       |   ||     | |  |-------------------------------1
```

```
                    |    ||      | |-----------------------------------1
                    |    ||      |-------------------------------------1
                    |    ||-------------------------------------------5
                    |    |--------------------------------------------4
                    |-------------------------------------------------1

>miR156g
    Score: 1.0  Deg: 5:1901:5:45   T_00071657

Query:          1 TTGACAGAAGAGAGAGAGCAC
 22
                  *|||||||||||||||||||*
Sbjct:       1911 TACTGTCTTCTCTCTCTCGTGT 1890
                    |    ||      | |  |--------------------------------1
                    |    ||      | |-----------------------------------1
                    |    ||      |-------------------------------------1
                    |    ||-------------------------------------------5
                    |    |--------------------------------------------4
                    |-------------------------------------------------1

>miR156g
    Score: 1.0  Deg: 5:1222:5:44   T_00071664

Query:          1 TTGACAGAAGAGAGAGAGCAC
 22
                  *|||||||||||||||||||*
Sbjct:       1232 TACTGTCTTCTCTCTCTCGTGT 1211
                    |    ||      | |  |--------------------------------1
                    |    ||      | |-----------------------------------1
                    |    ||      |-------------------------------------1
                    |    ||-------------------------------------------5
                    |    |--------------------------------------------4
                    |-------------------------------------------------1

>miR156g
    Score: 1.0  Deg: 5:2528:631:10547  T_00071658

Query:          1 TTGACAGAAGAGAGAGAGCAC
 22
                  *|||||||||||||||||||*
Sbjct:       2538 TACTGTCTTCTCTCTCTCGTGT 2517
                    |    ||      | |  |--------------------------------1
                    |    ||      | |-----------------------------------1
                    |    ||      |-------------------------------------1
                    |    ||-------------------------------------------5
```

```
                       |   |-------------------------------------------4
                       |-----------------------------------------------1

>miR156g
    Score: 1.0  Deg: 5:1457:5:53   T_00071665

Query:          1 TTGACAGAAGAGAGAGAGCAC
 22
                  *|||||||||||||||||||*
Sbjct:       1467 TACTGTCTTCTCTCTCTCGTGT 1446
                       |   ||     | |  |-------------------------------1
                       |   ||     | |----------------------------------1
                       |   ||     |------------------------------------1
                       |   ||------------------------------------------5
                       |   |-------------------------------------------4
                       |-----------------------------------------------1

>miR156g
    Score: 1.0  Deg: 5:2084:631:10538  T_00071656

Query:          1 TTGACAGAAGAGAGAGAGCAC
 22
                  *|||||||||||||||||||*
Sbjct:       2094 TACTGTCTTCTCTCTCTCGTGT 2073
                       |   ||     | |  |-------------------------------1
                       |   ||     | |----------------------------------1
                       |   ||     |------------------------------------1
                       |   ||------------------------------------------5
                       |   |-------------------------------------------4
                       |-----------------------------------------------1

>miR156g
    Score: 1.0  Deg: 5:1826:5:53   T_00071661

Query:          1 TTGACAGAAGAGAGAGAGCAC
 22
                  *|||||||||||||||||||*
Sbjct:       1836 TACTGTCTTCTCTCTCTCGTGT 1815
                       |   ||     | |  |-------------------------------1
                       |   ||     | |----------------------------------1
                       |   ||     |------------------------------------1
                       |   ||------------------------------------------5
                       |   |-------------------------------------------4
                       |-----------------------------------------------1
```

```
>miR156g
    Score: 1.0  Deg: 5:1160:5:44   T_00071667

Query:          1 TTGACAGAAGAGAGAGAGCAC
 22
                  *|||||||||||||||||||*
Sbjct:       1170 TACTGTCTTCTCTCTCTCGTGT 1149
                       |   ||     | |  |-------------------------------1
                       |   ||     | |----------------------------------1
                       |   ||     |------------------------------------1
                       |   ||-----------------------------------------5
                       |   |------------------------------------------4
                       |----------------------------------------------1

>miR156g
    Score: 1.0  Deg: 5:2345:5:54   T_00071659

Query:          1 TTGACAGAAGAGAGAGAGCAC
 22
                  *|||||||||||||||||||*
Sbjct:       2355 TACTGTCTTCTCTCTCTCGTGT 2334
                       |   ||     | |  |-------------------------------1
                       |   ||     | |----------------------------------1
                       |   ||     |------------------------------------1
                       |   ||-----------------------------------------5
                       |   |------------------------------------------4
                       |----------------------------------------------1

>miR156g
    Score: 1.0  Deg: 5:1931:631:10538  T_00071660

Query:          1 TTGACAGAAGAGAGAGAGCAC
 22
                  *|||||||||||||||||||*
Sbjct:       1941 TACTGTCTTCTCTCTCTCGTGT 1920
                       |   ||     | |  |-------------------------------1
                       |   ||     | |----------------------------------1
                       |   ||     |------------------------------------1
                       |   ||-----------------------------------------5
                       |   |------------------------------------------4
                       |----------------------------------------------1

>miR156g
    Score: 1.0  Deg: 5:2217:631:10547  T_00071662
```

```
Query:          1 TTGACAGAAGAGAGAGAGCAC
 22
                  *|||||||||||||||||||*
Sbjct:       2227 TACTGTCTTCTCTCTCTCGTGT 2206
                       |   ||     | |  |-------------------------------1
                       |   ||     | |----------------------------------1
                       |   ||     |------------------------------------1
                       |   ||------------------------------------------5
                       |   |-------------------------------------------4
                       |-----------------------------------------------1

>miR156g
    Score: 1.0  Deg: 5:1161:5:44   T_00071666

Query:          1 TTGACAGAAGAGAGAGAGCAC
 22
                  *|||||||||||||||||||*
Sbjct:       1171 TACTGTCTTCTCTCTCTCGTGT 1150
                       |   ||     | |  |-------------------------------1
                       |   ||     | |----------------------------------1
                       |   ||     |------------------------------------1
                       |   ||------------------------------------------5
                       |   |-------------------------------------------4
                       |-----------------------------------------------1

>miR156h-5p
    Score: 1.5  Deg: 10:1311:27:223T_00091783

Query:          1 TGACAGAAGAGAGTGAGCAC
 21
                  ||||||||||||*||||||*
Sbjct:       1319 ACTGTCTTCTCTCTCTCGTGT 1299
                         ||||    |    |------------------------------1
                         ||||    |-----------------------------------2
                         ||||-----------------------------------------6
                         |||------------------------------------------6
                         ||-------------------------------------------10
                         |--------------------------------------------1

>miR156h-5p
    Score: 1.5  Deg: 10:1668:27:226T_00091779

Query:          1 TGACAGAAGAGAGTGAGCAC
 21
                  ||||||||||||*||||||*
```

```
Sbjct:       1676 ACTGTCTTCTCTCTCTCGTGT 1656
                         ||||    |    |------------------------------1
                         ||||    |-----------------------------------2
                         ||||----------------------------------------6
                         |||-----------------------------------------6
                         ||------------------------------------------10
                         |-------------------------------------------1
```

>miR156h-5p
    Score: 1.5  Deg: 10:2823:27:234T_00091781

```
Query:          1 TGACAGAAGAGAGTGAGCAC
 21
                  ||||||||||||*|||||*
Sbjct:       2831 ACTGTCTTCTCTCTCTCGTGT 2811
                         ||||    |    |------------------------------1
                         ||||    |-----------------------------------2
                         ||||----------------------------------------6
                         |||-----------------------------------------6
                         ||------------------------------------------10
                         |-------------------------------------------1
```

>miR156h-5p
    Score: 1.5  Deg: 10:1470:27:223T_00091782

```
Query:          1 TGACAGAAGAGAGTGAGCAC
 21
                  ||||||||||||*|||||*
Sbjct:       1478 ACTGTCTTCTCTCTCTCGTGT 1458
                         ||||    |    |------------------------------1
                         ||||    |-----------------------------------2
                         ||||----------------------------------------6
                         |||-----------------------------------------6
                         ||------------------------------------------10
                         |-------------------------------------------1
```

>miR156h-5p
    Score: 1.5  Deg: 10:1199:27:223T_00091784

```
Query:          1 TGACAGAAGAGAGTGAGCAC
 21
                  ||||||||||||*|||||*
Sbjct:       1207 ACTGTCTTCTCTCTCTCGTGT 1187
                         ||||    |    |------------------------------1
                         ||||    |-----------------------------------2
```

```
                         ||||-----------------------------------------6
                         |||------------------------------------------6
                         ||-------------------------------------------10
                         |--------------------------------------------1
```

>miR156h-5p
    Score: 1.5  Deg: 10:1496:27:226T_00091780

```
Query:          1 TGACAGAAGAGAGTGAGCAC
 21
                  ||||||||||||*||||||*
Sbjct:       1504 ACTGTCTTCTCTCTCTCGTGT 1484
                         ||||    |    |-------------------------------1
                         ||||    |------------------------------------2
                         ||||-----------------------------------------6
                         |||------------------------------------------6
                         ||-------------------------------------------10
                         |--------------------------------------------1
```

>miR156h-5p
    Score: 2.5  Deg: 22:1017:24:363T_00090332

```
Query:          1 TGACAGAAGAGAGTGAGCAC
 21
                  ||||||||||*||*||||||*
Sbjct:       1025 ACTGTCTTCTATCTCTCGTGC 1005
                      | | ||||||  |-----------------------------------2
                      | | ||||||--------------------------------------1
                      | | |||||---------------------------------------1
                      | | ||||----------------------------------------1
                      | | |||-----------------------------------------2
                      | | ||------------------------------------------3
                      | | |-------------------------------------------22
                      | |---------------------------------------------1
                      |-----------------------------------------------2
```

>miR156h-5p
    Score: 2.5  Deg: 22:1088:24:371T_00090336

```
Query:          1 TGACAGAAGAGAGTGAGCAC
 21
                  ||||||||||*||*||||||*
Sbjct:       1096 ACTGTCTTCTATCTCTCGTGC 1076
                      | | ||||||  |-----------------------------------2
                      | | ||||||--------------------------------------1
```

```
                    | | |||||-----------------------------------------1
                    | | ||||------------------------------------------1
                    | | |||-------------------------------------------2
                    | | ||--------------------------------------------3
                    | | |---------------------------------------------22
                    | |-----------------------------------------------1
                    |-------------------------------------------------2
```

>miR156h-5p
    Score: 2.5  Deg: 22:1171:24:378T_00090333

```
Query:          1 TGACAGAAGAGAGTGAGCAC
 21
                  ||||||||||*||*||||||*
Sbjct:       1179 ACTGTCTTCTATCTCTCGTGC 1159
                    | | ||||||   |------------------------------------2
                    | | ||||||----------------------------------------1
                    | | |||||-----------------------------------------1
                    | | ||||------------------------------------------1
                    | | |||-------------------------------------------2
                    | | ||--------------------------------------------3
                    | | |---------------------------------------------22
                    | |-----------------------------------------------1
                    |-------------------------------------------------2
```

>miR156h-5p
    Score: 2.5  Deg: 22:1129:24:363T_00090331

```
Query:          1 TGACAGAAGAGAGTGAGCAC
 21
                  ||||||||||*||*||||||*
Sbjct:       1137 ACTGTCTTCTATCTCTCGTGC 1117
                    | | ||||||   |------------------------------------2
                    | | ||||||----------------------------------------1
                    | | |||||-----------------------------------------1
                    | | ||||------------------------------------------1
                    | | |||-------------------------------------------2
                    | | ||--------------------------------------------3
                    | | |---------------------------------------------22
                    | |-----------------------------------------------1
                    |-------------------------------------------------2
```

>miR156h-5p
    Score: 2.5  Deg: 22:958:24:363 T_00090335

```
Query:          1 TGACAGAAGAGAGTGAGCAC
 21
                  ||||||||||*||*|||||*
Sbjct:        966 ACTGTCTTCTATCTCTCGTGC 946
                      | | ||||||  |----------------------------------2
                      | | ||||||-------------------------------------1
                      | | |||||--------------------------------------1
                      | | ||||---------------------------------------1
                      | | |||----------------------------------------2
                      | | ||-----------------------------------------3
                      | | |------------------------------------------22
                      | |--------------------------------------------1
                      |----------------------------------------------2
```

>miR156h-5p

```
    Score: 2.5  Deg: 22:941:24:361 T_00090334

Query:          1 TGACAGAAGAGAGTGAGCAC
 21
                  ||||||||||*||*|||||*
Sbjct:        949 ACTGTCTTCTATCTCTCGTGC 929
                      | | ||||||  |----------------------------------2
                      | | ||||||-------------------------------------1
                      | | |||||--------------------------------------1
                      | | ||||---------------------------------------1
                      | | |||----------------------------------------2
                      | | ||-----------------------------------------3
                      | | |------------------------------------------22
                      | |--------------------------------------------1
                      |----------------------------------------------2
```

>miR156h-5p

```
    Score: 2.0  Deg: 79:1218:79:519T_00059648

Query:          1 TGACAGAAGAGAGTGAGCAC
 21
                  |||||||||||||*|||||**
Sbjct:       1226 ACTGTCTTCTCTCTCTCGTAC 1206
                  ||   ||||||    |||---------------------------------1
                  ||   ||||||    ||----------------------------------3
                  ||   ||||||    |-----------------------------------2
                  ||   ||||||----------------------------------------2
                  ||   |||||-----------------------------------------50
                  ||   ||||------------------------------------------79
                  ||   |||-------------------------------------------3
```

```
                   ||    ||-------------------------------------------3
                   ||    |--------------------------------------------1
                   ||---------------------------------------------------1
                   |----------------------------------------------------1

>miR156h-5p
    Score: 1.5  Deg: 9:1100:9:46   T_00080616

Query:          1 TGACAGAAGAGAGTGAGCAC
 21
                  ||||||||||||*||||||*
Sbjct:       1109 ACTGTCTTCTCTCTCTCGTGT 1089
                          ||    |     | |------------------------------1
                          ||    |     |--------------------------------1
                          ||    |--------------------------------------6
                          ||-------------------------------------------9
                          |--------------------------------------------4

>miR156h-5p
    Score: 1.5  Deg: 9:1250:9:33   T_00080613

Query:          1 TGACAGAAGAGAGTGAGCAC
 21
                  ||||||||||||*||||||*
Sbjct:       1259 ACTGTCTTCTCTCTCTCGTGT 1239
                          ||    |     | |------------------------------1
                          ||    |     |--------------------------------1
                          ||    |--------------------------------------6
                          ||-------------------------------------------9
                          |--------------------------------------------4

>miR156h-5p
    Score: 1.5  Deg: 9:1195:9:64   T_00080614

Query:          1 TGACAGAAGAGAGTGAGCAC
 21
                  ||||||||||||*||||||*
Sbjct:       1204 ACTGTCTTCTCTCTCTCGTGT 1184
                          ||    |     | |------------------------------1
                          ||    |     |--------------------------------1
                          ||    |--------------------------------------6
                          ||-------------------------------------------9
                          |--------------------------------------------4

>miR156h-5p
```

```
    Score: 1.5  Deg: 9:1124:9:46   T_00080615

Query:          1 TGACAGAAGAGAGTGAGCAC
 21
                  ||||||||||||*|||||*
Sbjct:       1133 ACTGTCTTCTCTCTCTCGTGT 1113
                           ||   |    | |-------------------------------1
                           ||   |    |---------------------------------1
                           ||   |--------------------------------------6
                           ||------------------------------------------9
                           |-------------------------------------------4

>miR156h-5p
    Score: 1.5  Deg: 9:1250:9:64   T_00080612

Query:          1 TGACAGAAGAGAGTGAGCAC
 21
                  ||||||||||||*|||||*
Sbjct:       1259 ACTGTCTTCTCTCTCTCGTGT 1239
                           ||   |    | |-------------------------------1
                           ||   |    |---------------------------------1
                           ||   |--------------------------------------6
                           ||------------------------------------------9
                           |-------------------------------------------4

>miR156h-5p
    Score: 1.5  Deg: 12:1418:12:64 T_00046313

Query:          1 TGACAGAAGAGAGTGAGCAC
 21
                  ||||||||||||*|||||*
Sbjct:       1427 ACTGTCTTCTCTCTCTCGTGT 1407
                       |   |||  |   |  |-------------------------------1
                       |   |||  |   |----------------------------------1
                       |   |||  |--------------------------------------4
                       |   |||-----------------------------------------1
                       |   ||------------------------------------------12
                       |   |-------------------------------------------10
                       |-----------------------------------------------2

>miR156h-5p
    Score: 1.5  Deg: 77:2106:83:749T_00052738

Query:          1 TGACAGAAGAGAGTGAGCAC
 21
```

```
                 |||||||||||||*||||||*
Sbjct:      2114 ACTGTCTTCTCTCTCTCGTGC 2094
                       | ||||||| |||----------------------------------1
                       | ||||||| ||-----------------------------------1
                       | ||||||| |------------------------------------1
                       | |||||||--------------------------------------1
                       | ||||||---------------------------------------1
                       | |||||----------------------------------------1
                       | ||||-----------------------------------------1
                       | |||------------------------------------------9
                       | ||-------------------------------------------56
                       | |--------------------------------------------77
                       |----------------------------------------------1
```

```
>miR156h-5p
    Score: 1.5  Deg: 77:443:77:327 T_00052739

Query:         1 TGACAGAAGAGAGTGAGCAC
 21
                 |||||||||||||*||||||*
Sbjct:       451 ACTGTCTTCTCTCTCTCGTGC 431
                       | ||||||| |||----------------------------------1
                       | ||||||| ||-----------------------------------1
                       | ||||||| |------------------------------------1
                       | |||||||--------------------------------------1
                       | ||||||---------------------------------------1
                       | |||||----------------------------------------1
                       | ||||-----------------------------------------1
                       | |||------------------------------------------9
                       | ||-------------------------------------------56
                       | |--------------------------------------------77
                       |----------------------------------------------1
```

```
>miR156h-5p
    Score: 1.5  Deg: 77:2114:83:749T_00052737

Query:         1 TGACAGAAGAGAGTGAGCAC
 21
                 |||||||||||||*||||||*
Sbjct:      2122 ACTGTCTTCTCTCTCTCGTGC 2102
                       | ||||||| |||----------------------------------1
                       | ||||||| ||-----------------------------------1
                       | ||||||| |------------------------------------1
                       | |||||||--------------------------------------1
                       | ||||||---------------------------------------1
```

```
                         | |||||--------------------------------------1
                         | ||||---------------------------------------1
                         | |||----------------------------------------9
                         | ||-----------------------------------------56
                         | |------------------------------------------77
                         |--------------------------------------------1

>miR156h-5p
    Score: 1.5  Deg: 5:2012:5:54   T_00071663

Query:          1 TGACAGAAGAGAGTGAGCAC
 21
                  |||||||||||||*||||||*
Sbjct:       2021 ACTGTCTTCTCTCTCTCGTGT 2001
                       |   ||     | |  |------------------------------1
                       |   ||     | |---------------------------------1
                       |   ||     |-----------------------------------1
                       |   ||-----------------------------------------5
                       |   |------------------------------------------4
                       |----------------------------------------------1

>miR156h-5p
    Score: 1.5  Deg: 5:1901:5:45   T_00071657

Query:          1 TGACAGAAGAGAGTGAGCAC
 21
                  |||||||||||||*||||||*
Sbjct:       1910 ACTGTCTTCTCTCTCTCGTGT 1890
                       |   ||     | |  |------------------------------1
                       |   ||     | |---------------------------------1
                       |   ||     |-----------------------------------1
                       |   ||-----------------------------------------5
                       |   |------------------------------------------4
                       |----------------------------------------------1

>miR156h-5p
    Score: 1.5  Deg: 5:1222:5:44   T_00071664

Query:          1 TGACAGAAGAGAGTGAGCAC
 21
                  |||||||||||||*||||||*
Sbjct:       1231 ACTGTCTTCTCTCTCTCGTGT 1211
                       |   ||     | |  |------------------------------1
                       |   ||     | |---------------------------------1
                       |   ||     |-----------------------------------1
```

```
                |    ||-----------------------------------------5
                |    |------------------------------------------4
                |-----------------------------------------------1
```

```
>miR156h-5p
    Score: 1.5  Deg: 5:2528:631:10547  T_00071658

Query:          1 TGACAGAAGAGAGTGAGCAC
 21
                  ||||||||||||*|||||*
Sbjct:       2537 ACTGTCTTCTCTCTCTCGTGT 2517
                |    ||      | |  |-------------------------------1
                |    ||      | |----------------------------------1
                |    ||      |------------------------------------1
                |    ||-----------------------------------------5
                |    |------------------------------------------4
                |-----------------------------------------------1
```

```
>miR156h-5p
    Score: 1.5  Deg: 5:1457:5:53   T_00071665

Query:          1 TGACAGAAGAGAGTGAGCAC
 21
                  ||||||||||||*|||||*
Sbjct:       1466 ACTGTCTTCTCTCTCTCGTGT 1446
                |    ||      | |  |-------------------------------1
                |    ||      | |----------------------------------1
                |    ||      |------------------------------------1
                |    ||-----------------------------------------5
                |    |------------------------------------------4
                |-----------------------------------------------1
```

```
>miR156h-5p
    Score: 1.5  Deg: 5:2084:631:10538  T_00071656

Query:          1 TGACAGAAGAGAGTGAGCAC
 21
                  ||||||||||||*|||||*
Sbjct:       2093 ACTGTCTTCTCTCTCTCGTGT 2073
                |    ||      | |  |-------------------------------1
                |    ||      | |----------------------------------1
                |    ||      |------------------------------------1
                |    ||-----------------------------------------5
                |    |------------------------------------------4
                |-----------------------------------------------1
```

```
>miR156h-5p
    Score: 1.5  Deg: 5:1826:5:53   T_00071661

Query:          1 TGACAGAAGAGAGTGAGCAC
 21
                  ||||||||||||*||||||*
Sbjct:       1835 ACTGTCTTCTCTCTCTCGTGT 1815
                      |   ||     | |  |-------------------------------1
                      |   ||     | |----------------------------------1
                      |   ||     |------------------------------------1
                      |   ||------------------------------------------5
                      |   |-------------------------------------------4
                      |-----------------------------------------------1

>miR156h-5p
    Score: 1.5  Deg: 5:1160:5:44   T_00071667

Query:          1 TGACAGAAGAGAGTGAGCAC
 21
                  ||||||||||||*||||||*
Sbjct:       1169 ACTGTCTTCTCTCTCTCGTGT 1149
                      |   ||     | |  |-------------------------------1
                      |   ||     | |----------------------------------1
                      |   ||     |------------------------------------1
                      |   ||------------------------------------------5
                      |   |-------------------------------------------4
                      |-----------------------------------------------1

>miR156h-5p
    Score: 1.5  Deg: 5:2345:5:54   T_00071659

Query:          1 TGACAGAAGAGAGTGAGCAC
 21
                  ||||||||||||*||||||*
Sbjct:       2354 ACTGTCTTCTCTCTCTCGTGT 2334
                      |   ||     | |  |-------------------------------1
                      |   ||     | |----------------------------------1
                      |   ||     |------------------------------------1
                      |   ||------------------------------------------5
                      |   |-------------------------------------------4
                      |-----------------------------------------------1

>miR156h-5p
    Score: 1.5  Deg: 5:1931:631:10538  T_00071660
```

```
Query:          1 TGACAGAAGAGAGTGAGCAC
 21
                  |||||||||||||*||||||*
Sbjct:       1940 ACTGTCTTCTCTCTCTCGTGT 1920
                      |   ||     | |  |-------------------------------1
                      |   ||     | |----------------------------------1
                      |   ||     |------------------------------------1
                      |   ||------------------------------------------5
                      |   |-------------------------------------------4
                      |-----------------------------------------------1

>miR156h-5p
    Score: 1.5  Deg: 5:2217:631:10547  T_00071662

Query:          1 TGACAGAAGAGAGTGAGCAC
 21
                  |||||||||||||*||||||*
Sbjct:       2226 ACTGTCTTCTCTCTCTCGTGT 2206
                      |   ||     | |  |-------------------------------1
                      |   ||     | |----------------------------------1
                      |   ||     |------------------------------------1
                      |   ||------------------------------------------5
                      |   |-------------------------------------------4
                      |-----------------------------------------------1

>miR156h-5p
    Score: 1.5  Deg: 5:1161:5:44   T_00071666

Query:          1 TGACAGAAGAGAGTGAGCAC
 21
                  |||||||||||||*||||||*
Sbjct:       1170 ACTGTCTTCTCTCTCTCGTGT 1150
                      |   ||     | |  |-------------------------------1
                      |   ||     | |----------------------------------1
                      |   ||     |------------------------------------1
                      |   ||------------------------------------------5
                      |   |-------------------------------------------4
                      |-----------------------------------------------1

>miR156i-5p
    Score: 1.5  Deg: 10:1311:27:223T_00091783

Query:          1 TGACAGAAGAGAGTGAGCAC
 21
```

```
                 |||||||||||||*||||||*
Sbjct:       1319 ACTGTCTTCTCTCTCTCGTGT 1299
                         ||||    |    |------------------------------1
                         ||||    |-----------------------------------2
                         ||||----------------------------------------6
                         |||-----------------------------------------6
                         ||------------------------------------------10
                         |-------------------------------------------1

>miR156i-5p
    Score: 1.5  Deg: 10:1668:27:226T_00091779

Query:          1 TGACAGAAGAGAGTGAGCAC
 21
                 |||||||||||||*||||||*
Sbjct:       1676 ACTGTCTTCTCTCTCTCGTGT 1656
                         ||||    |    |------------------------------1
                         ||||    |-----------------------------------2
                         ||||----------------------------------------6
                         |||-----------------------------------------6
                         ||------------------------------------------10
                         |-------------------------------------------1

>miR156i-5p
    Score: 1.5  Deg: 10:2823:27:234T_00091781

Query:          1 TGACAGAAGAGAGTGAGCAC
 21
                 |||||||||||||*||||||*
Sbjct:       2831 ACTGTCTTCTCTCTCTCGTGT 2811
                         ||||    |    |------------------------------1
                         ||||    |-----------------------------------2
                         ||||----------------------------------------6
                         |||-----------------------------------------6
                         ||------------------------------------------10
                         |-------------------------------------------1

>miR156i-5p
    Score: 1.5  Deg: 10:1470:27:223T_00091782

Query:          1 TGACAGAAGAGAGTGAGCAC
 21
                 |||||||||||||*||||||*
Sbjct:       1478 ACTGTCTTCTCTCTCTCGTGT 1458
                         ||||    |    |------------------------------1
```

```
                         ||||    |-----------------------------------2
                         ||||-----------------------------------------6
                         |||------------------------------------------6
                         ||-------------------------------------------10
                         |--------------------------------------------1

>miR156i-5p
    Score: 1.5  Deg: 10:1199:27:223T_00091784

Query:          1 TGACAGAAGAGAGTGAGCAC
 21
                  ||||||||||||*|||||*
Sbjct:       1207 ACTGTCTTCTCTCTCTCGTGT 1187
                         ||||    |    |------------------------------1
                         ||||    |-----------------------------------2
                         ||||-----------------------------------------6
                         |||------------------------------------------6
                         ||-------------------------------------------10
                         |--------------------------------------------1

>miR156i-5p
    Score: 1.5  Deg: 10:1496:27:226T_00091780

Query:          1 TGACAGAAGAGAGTGAGCAC
 21
                  ||||||||||||*|||||*
Sbjct:       1504 ACTGTCTTCTCTCTCTCGTGT 1484
                         ||||    |    |------------------------------1
                         ||||    |-----------------------------------2
                         ||||-----------------------------------------6
                         |||------------------------------------------6
                         ||-------------------------------------------10
                         |--------------------------------------------1

>miR156i-5p
    Score: 2.5  Deg: 22:1017:24:363T_00090332

Query:          1 TGACAGAAGAGAGTGAGCAC
 21
                  |||||||||*||*|||||*
Sbjct:       1025 ACTGTCTTCTATCTCTCGTGC 1005
                      | | ||||||  |---------------------------------2
                      | | ||||||---------------------------------------1
                      | | |||||----------------------------------------1
                      | | ||||-----------------------------------------1
```

```
                    | | |||-----------------------------------------2
                    | | ||------------------------------------------3
                    | | |-------------------------------------------22
                    | |---------------------------------------------1
                    |-----------------------------------------------2

>miR156i-5p
    Score: 2.5  Deg: 22:1088:24:371T_00090336

Query:          1 TGACAGAAGAGAGTGAGCAC
 21
                  ||||||||||*||*||||||*
Sbjct:       1096 ACTGTCTTCTATCTCTCGTGC 1076
                    | | ||||||  |-----------------------------------2
                    | | ||||||--------------------------------------1
                    | | |||||---------------------------------------1
                    | | ||||----------------------------------------1
                    | | |||-----------------------------------------2
                    | | ||------------------------------------------3
                    | | |-------------------------------------------22
                    | |---------------------------------------------1
                    |-----------------------------------------------2

>miR156i-5p
    Score: 2.5  Deg: 22:1171:24:378T_00090333

Query:          1 TGACAGAAGAGAGTGAGCAC
 21
                  ||||||||||*||*||||||*
Sbjct:       1179 ACTGTCTTCTATCTCTCGTGC 1159
                    | | ||||||  |-----------------------------------2
                    | | ||||||--------------------------------------1
                    | | |||||---------------------------------------1
                    | | ||||----------------------------------------1
                    | | |||-----------------------------------------2
                    | | ||------------------------------------------3
                    | | |-------------------------------------------22
                    | |---------------------------------------------1
                    |-----------------------------------------------2

>miR156i-5p
    Score: 2.5  Deg: 22:1129:24:363T_00090331

Query:          1 TGACAGAAGAGAGTGAGCAC
 21
```

```
                    ||||||||||*||*||||||*
Sbjct:       1137 ACTGTCTTCTATCTCTCGTGC 1117
                        | | ||||||  |-----------------------------------2
                        | | ||||||---------------------------------------1
                        | | |||||----------------------------------------1
                        | | ||||-----------------------------------------1
                        | | |||------------------------------------------2
                        | | ||-------------------------------------------3
                        | | |--------------------------------------------22
                        | |----------------------------------------------1
                        |------------------------------------------------2
```

>miR156i-5p

```
    Score: 2.5  Deg: 22:958:24:363 T_00090335

Query:           1 TGACAGAAGAGAGTGAGCAC
 21
                    ||||||||||*||*||||||*
Sbjct:        966 ACTGTCTTCTATCTCTCGTGC 946
                        | | ||||||  |-----------------------------------2
                        | | ||||||---------------------------------------1
                        | | |||||----------------------------------------1
                        | | ||||-----------------------------------------1
                        | | |||------------------------------------------2
                        | | ||-------------------------------------------3
                        | | |--------------------------------------------22
                        | |----------------------------------------------1
                        |------------------------------------------------2
```

>miR156i-5p

```
    Score: 2.5  Deg: 22:941:24:361 T_00090334

Query:           1 TGACAGAAGAGAGTGAGCAC
 21
                    ||||||||||*||*||||||*
Sbjct:        949 ACTGTCTTCTATCTCTCGTGC 929
                        | | ||||||  |-----------------------------------2
                        | | ||||||---------------------------------------1
                        | | |||||----------------------------------------1
                        | | ||||-----------------------------------------1
                        | | |||------------------------------------------2
                        | | ||-------------------------------------------3
                        | | |--------------------------------------------22
                        | |----------------------------------------------1
                        |------------------------------------------------2
```

```
>miR156i-5p
    Score: 2.0  Deg: 79:1218:79:519T_00059648

Query:           1 TGACAGAAGAGAGTGAGCAC
 21
                  |||||||||||||*|||||**
Sbjct:       1226 ACTGTCTTCTCTCTCTCGTAC 1206
                  ||   ||||||    |||----------------------------------1
                  ||   ||||||    ||-----------------------------------3
                  ||   ||||||    |------------------------------------2
                  ||   ||||||-----------------------------------------2
                  ||   |||||------------------------------------------50
                  ||   ||||-------------------------------------------79
                  ||   |||--------------------------------------------3
                  ||   ||---------------------------------------------3
                  ||   |----------------------------------------------1
                  ||--------------------------------------------------1
                  |---------------------------------------------------1

>miR156i-5p
    Score: 1.5  Deg: 9:1100:9:46   T_00080616

Query:           1 TGACAGAAGAGAGTGAGCAC
 21
                  |||||||||||||*||||||*
Sbjct:       1109 ACTGTCTTCTCTCTCTCGTGT 1089
                          ||   |    | |-------------------------------1
                          ||   |    |---------------------------------1
                          ||   |--------------------------------------6
                          ||------------------------------------------9
                          |-------------------------------------------4

>miR156i-5p
    Score: 1.5  Deg: 9:1250:9:33   T_00080613

Query:           1 TGACAGAAGAGAGTGAGCAC
 21
                  |||||||||||||*||||||*
Sbjct:       1259 ACTGTCTTCTCTCTCTCGTGT 1239
                          ||   |    | |-------------------------------1
                          ||   |    |---------------------------------1
                          ||   |--------------------------------------6
                          ||------------------------------------------9
                          |-------------------------------------------4
```

```
>miR156i-5p
    Score: 1.5  Deg: 9:1195:9:64   T_00080614

Query:          1 TGACAGAAGAGAGTGAGCAC
 21
                  ||||||||||||*||||||*
Sbjct:       1204 ACTGTCTTCTCTCTCTCGTGT 1184
                          ||   |    | |------------------------------1
                          ||   |    |--------------------------------1
                          ||   |-------------------------------------6
                          ||-----------------------------------------9
                          |------------------------------------------4

>miR156i-5p
    Score: 1.5  Deg: 9:1124:9:46   T_00080615

Query:          1 TGACAGAAGAGAGTGAGCAC
 21
                  ||||||||||||*||||||*
Sbjct:       1133 ACTGTCTTCTCTCTCTCGTGT 1113
                          ||   |    | |------------------------------1
                          ||   |    |--------------------------------1
                          ||   |-------------------------------------6
                          ||-----------------------------------------9
                          |------------------------------------------4

>miR156i-5p
    Score: 1.5  Deg: 9:1250:9:64   T_00080612

Query:          1 TGACAGAAGAGAGTGAGCAC
 21
                  ||||||||||||*||||||*
Sbjct:       1259 ACTGTCTTCTCTCTCTCGTGT 1239
                          ||   |    | |------------------------------1
                          ||   |    |--------------------------------1
                          ||   |-------------------------------------6
                          ||-----------------------------------------9
                          |------------------------------------------4

>miR156i-5p
    Score: 1.5  Deg: 12:1418:12:64 T_00046313

Query:          1 TGACAGAAGAGAGTGAGCAC
 21
```

```
                  |||||||||||||*||||||*
Sbjct:       1427 ACTGTCTTCTCTCTCTCGTGT 1407
                      |   |||  |   |  |-----------------------------1
                      |   |||  |   |--------------------------------1
                      |   |||  |------------------------------------4
                      |   |||---------------------------------------1
                      |   ||----------------------------------------12
                      |   |-----------------------------------------10
                      |---------------------------------------------2
```

```
>miR156i-5p
    Score: 1.5  Deg: 77:2106:83:749T_00052738

Query:            1 TGACAGAAGAGAGTGAGCAC
 21
                  |||||||||||||*||||||*
Sbjct:       2114 ACTGTCTTCTCTCTCTCGTGC 2094
                        | ||||||| |||-------------------------------1
                        | ||||||| ||--------------------------------1
                        | ||||||| |---------------------------------1
                        | |||||||-----------------------------------1
                        | ||||||------------------------------------1
                        | |||||-------------------------------------1
                        | ||||--------------------------------------1
                        | |||---------------------------------------9
                        | ||----------------------------------------56
                        | |-----------------------------------------77
                        |-------------------------------------------1
```

```
>miR156i-5p
    Score: 1.5  Deg: 77:443:77:327 T_00052739

Query:            1 TGACAGAAGAGAGTGAGCAC
 21
                  |||||||||||||*||||||*
Sbjct:        451 ACTGTCTTCTCTCTCTCGTGC 431
                        | ||||||| |||-------------------------------1
                        | ||||||| ||--------------------------------1
                        | ||||||| |---------------------------------1
                        | |||||||-----------------------------------1
                        | ||||||------------------------------------1
                        | |||||-------------------------------------1
                        | ||||--------------------------------------1
                        | |||---------------------------------------9
                        | ||----------------------------------------56
```

```
                        | |-------------------------------------------77
                        |---------------------------------------------1

>miR156i-5p
    Score: 1.5  Deg: 77:2114:83:749T_00052737

Query:          1 TGACAGAAGAGAGTGAGCAC
 21
                  |||||||||||||*||||||*
Sbjct:       2122 ACTGTCTTCTCTCTCTCGTGC 2102
                        | ||||||| |||---------------------------------1
                        | ||||||| ||----------------------------------1
                        | ||||||| |-----------------------------------1
                        | |||||||-------------------------------------1
                        | ||||||--------------------------------------1
                        | |||||---------------------------------------1
                        | ||||----------------------------------------1
                        | |||-----------------------------------------9
                        | ||------------------------------------------56
                        | |-------------------------------------------77
                        |---------------------------------------------1

>miR156i-5p
    Score: 1.5  Deg: 5:2012:5:54   T_00071663

Query:          1 TGACAGAAGAGAGTGAGCAC
 21
                  |||||||||||||*||||||*
Sbjct:       2021 ACTGTCTTCTCTCTCTCGTGT 2001
                      |   ||     | |  |-------------------------------1
                      |   ||     | |----------------------------------1
                      |   ||     |------------------------------------1
                      |   ||------------------------------------------5
                      |   |-------------------------------------------4
                      |-----------------------------------------------1

>miR156i-5p
    Score: 1.5  Deg: 5:1901:5:45   T_00071657

Query:          1 TGACAGAAGAGAGTGAGCAC
 21
                  |||||||||||||*||||||*
Sbjct:       1910 ACTGTCTTCTCTCTCTCGTGT 1890
                      |   ||     | |  |-------------------------------1
                      |   ||     | |----------------------------------1
```

```
          |    ||      |-----------------------------------1
          |    ||------------------------------------------5
          |    |-------------------------------------------4
          |------------------------------------------------1
```

>miR156i-5p
    Score: 1.5  Deg: 5:1222:5:44   T_00071664

```
Query:          1 TGACAGAAGAGAGTGAGCAC
 21
                  |||||||||||||*||||||*
Sbjct:       1231 ACTGTCTTCTCTCTCTCGTGT 1211
                      |    ||      | |  |-------------------------------1
                      |    ||      | |----------------------------------1
                      |    ||      |-----------------------------------1
                      |    ||------------------------------------------5
                      |    |-------------------------------------------4
                      |------------------------------------------------1
```

>miR156i-5p
    Score: 1.5  Deg: 5:2528:631:10547  T_00071658

```
Query:          1 TGACAGAAGAGAGTGAGCAC
 21
                  |||||||||||||*||||||*
Sbjct:       2537 ACTGTCTTCTCTCTCTCGTGT 2517
                      |    ||      | |  |-------------------------------1
                      |    ||      | |----------------------------------1
                      |    ||      |-----------------------------------1
                      |    ||------------------------------------------5
                      |    |-------------------------------------------4
                      |------------------------------------------------1
```

>miR156i-5p
    Score: 1.5  Deg: 5:1457:5:53   T_00071665

```
Query:          1 TGACAGAAGAGAGTGAGCAC
 21
                  |||||||||||||*||||||*
Sbjct:       1466 ACTGTCTTCTCTCTCTCGTGT 1446
                      |    ||      | |  |-------------------------------1
                      |    ||      | |----------------------------------1
                      |    ||      |-----------------------------------1
                      |    ||------------------------------------------5
                      |    |-------------------------------------------4
```

```
                      |------------------------------------------------1

>miR156i-5p
    Score: 1.5  Deg: 5:2084:631:10538  T_00071656

Query:          1 TGACAGAAGAGAGTGAGCAC
 21
                  |||||||||||||*||||||*
Sbjct:       2093 ACTGTCTTCTCTCTCTCGTGT 2073
                      |   ||     | |  |--------------------------------1
                      |   ||     | |-----------------------------------1
                      |   ||     |-------------------------------------1
                      |   ||-------------------------------------------5
                      |   |--------------------------------------------4
                      |------------------------------------------------1

>miR156i-5p
    Score: 1.5  Deg: 5:1826:5:53   T_00071661

Query:          1 TGACAGAAGAGAGTGAGCAC
 21
                  |||||||||||||*||||||*
Sbjct:       1835 ACTGTCTTCTCTCTCTCGTGT 1815
                      |   ||     | |  |--------------------------------1
                      |   ||     | |-----------------------------------1
                      |   ||     |-------------------------------------1
                      |   ||-------------------------------------------5
                      |   |--------------------------------------------4
                      |------------------------------------------------1

>miR156i-5p
    Score: 1.5  Deg: 5:1160:5:44   T_00071667

Query:          1 TGACAGAAGAGAGTGAGCAC
 21
                  |||||||||||||*||||||*
Sbjct:       1169 ACTGTCTTCTCTCTCTCGTGT 1149
                      |   ||     | |  |--------------------------------1
                      |   ||     | |-----------------------------------1
                      |   ||     |-------------------------------------1
                      |   ||-------------------------------------------5
                      |   |--------------------------------------------4
                      |------------------------------------------------1

>miR156i-5p
```

```
    Score: 1.5  Deg: 5:2345:5:54   T_00071659

Query:          1 TGACAGAAGAGAGTGAGCAC
 21
                  ||||||||||||*|||||*
Sbjct:       2354 ACTGTCTTCTCTCTCTCGTGT 2334
                      |   ||     | |  |------------------------------1
                      |   ||     | |----------------------------------1
                      |   ||     |------------------------------------1
                      |   ||------------------------------------------5
                      |   |-------------------------------------------4
                      |-----------------------------------------------1

>miR156i-5p
    Score: 1.5  Deg: 5:1931:631:10538  T_00071660

Query:          1 TGACAGAAGAGAGTGAGCAC
 21
                  ||||||||||||*|||||*
Sbjct:       1940 ACTGTCTTCTCTCTCTCGTGT 1920
                      |   ||     | |  |------------------------------1
                      |   ||     | |----------------------------------1
                      |   ||     |------------------------------------1
                      |   ||------------------------------------------5
                      |   |-------------------------------------------4
                      |-----------------------------------------------1

>miR156i-5p
    Score: 1.5  Deg: 5:2217:631:10547  T_00071662

Query:          1 TGACAGAAGAGAGTGAGCAC
 21
                  ||||||||||||*|||||*
Sbjct:       2226 ACTGTCTTCTCTCTCTCGTGT 2206
                      |   ||     | |  |------------------------------1
                      |   ||     | |----------------------------------1
                      |   ||     |------------------------------------1
                      |   ||------------------------------------------5
                      |   |-------------------------------------------4
                      |-----------------------------------------------1

>miR156i-5p
    Score: 1.5  Deg: 5:1161:5:44   T_00071666

Query:          1 TGACAGAAGAGAGTGAGCAC
```

```
 21
                 |||||||||||||*||||||*
Sbjct:       1170 ACTGTCTTCTCTCTCTCGTGT 1150
                     |   ||     | |  |-------------------------------1
                     |   ||     | |----------------------------------1
                     |   ||     |------------------------------------1
                     |   ||------------------------------------------5
                     |   |-------------------------------------------4
                     |-----------------------------------------------1


>miR160a-5P
    Score: 1.0  Deg: 7:1830:8:191  T_00057636

Query:          1 TGCCTGGCTCCCTGTATGCCA
 22
                 ||||||||||||||||||||**
Sbjct:       1839 ACGGACCGAGGGACATACGGAC 1818
                          |    ||  |||--------------------------------1
                          |    ||  ||---------------------------------1
                          |    ||  |----------------------------------1
                          |    ||-------------------------------------3
                          |    |--------------------------------------1
                          |-------------------------------------------7

>miR160a-5P
    Score: 1.0  Deg: 7:1576:8:186  T_00057635

Query:          1 TGCCTGGCTCCCTGTATGCCA
 22
                 ||||||||||||||||||||**
Sbjct:       1585 ACGGACCGAGGGACATACGGAC 1564
                          |    ||  |||--------------------------------1
                          |    ||  ||---------------------------------1
                          |    ||  |----------------------------------1
                          |    ||-------------------------------------3
                          |    |--------------------------------------1
                          |-------------------------------------------7

>miR160a-5P
    Score: 1.0  Deg: 7:1535:8:185  T_00057634

Query:          1 TGCCTGGCTCCCTGTATGCCA
 22
                 ||||||||||||||||||||**
```

```
Sbjct:       1544 ACGGACCGAGGGACATACGGAC 1523
                           |    ||  |||--------------------------------1
                           |    ||  ||---------------------------------1
                           |    ||  |----------------------------------1
                           |    ||-------------------------------------3
                           |    |--------------------------------------1
                           |-------------------------------------------7
```

```
>miR160b-5P
    Score: 1.0  Deg: 7:1830:8:191  T_00057636

Query:          1 TGCCTGGCTCCCTGTATGCCA
 22
                  ||||||||||||||||||||**
Sbjct:       1839 ACGGACCGAGGGACATACGGAC 1818
                           |    ||  |||--------------------------------1
                           |    ||  ||---------------------------------1
                           |    ||  |----------------------------------1
                           |    ||-------------------------------------3
                           |    |--------------------------------------1
                           |-------------------------------------------7
```

```
>miR160b-5P
    Score: 1.0  Deg: 7:1576:8:186  T_00057635

Query:          1 TGCCTGGCTCCCTGTATGCCA
 22
                  ||||||||||||||||||||**
Sbjct:       1585 ACGGACCGAGGGACATACGGAC 1564
                           |    ||  |||--------------------------------1
                           |    ||  ||---------------------------------1
                           |    ||  |----------------------------------1
                           |    ||-------------------------------------3
                           |    |--------------------------------------1
                           |-------------------------------------------7
```

```
>miR160b-5P
    Score: 1.0  Deg: 7:1535:8:185  T_00057634

Query:          1 TGCCTGGCTCCCTGTATGCCA
 22
                  ||||||||||||||||||||**
Sbjct:       1544 ACGGACCGAGGGACATACGGAC 1523
                           |    ||  |||--------------------------------1
```

```
                           |    ||  ||---------------------------------1
                           |    ||  |----------------------------------1
                           |    ||-------------------------------------3
                           |    |--------------------------------------1
                           |-------------------------------------------7

>miR164a-5p
    Score: 1.0  Deg: 124:1010:124:445  T_00052644

Query:          1 TGGAGAAGCAGGGCACGTGCA
 22
                  |||||||||||||||||*||*
Sbjct:       1019 ACCTCTTCGTCCCGTGCAAGTA 998
                  ||   |  ||   || |  |---------------------------------1
                  ||   |  ||   || |------------------------------------2
                  ||   |  ||   ||--------------------------------------1
                  ||   |  ||   |---------------------------------------1
                  ||   |  ||-------------------------------------------124
                  ||   |  |--------------------------------------------4
                  ||   |-----------------------------------------------1
                  ||---------------------------------------------------1
                  |----------------------------------------------------1

>miR164a-5p
    Score: 2.0  Deg: 32:832:32:113 T_00059787

Query:          1 TGGAGAAGCAGGGCACGTGCA
 22
                  |||||||:||||:|||||||**
Sbjct:        841 ACCTCTTTGTCCTGTGCACGAG 820
                           |       |-----------------------------------1
                           |-------------------------------------------32

>miR164a-5p
    Score: 2.0  Deg: 32:813:32:113 T_00059786

Query:          1 TGGAGAAGCAGGGCACGTGCA
 22
                  |||||||:||||:|||||||**
Sbjct:        822 ACCTCTTTGTCCTGTGCACGAG 801
                           |       |-----------------------------------1
                           |-------------------------------------------32

>miR164a-5p
    Score: 2.2  Deg: 7:928:7:192   T_00079376
```

```
Query:          1 TGGAGAAGCAGGGCACGTGCA
 22
                  :|||||||||||*||||||*|*
Sbjct:        937 GCCTCTTCGTCCAGTGCAC-TC 917
                           |       |----------------------------------1
                           |------------------------------------------7

>miR164a-5p
    Score: 2.0  Deg: 7:955:205:1253T_00073807

Query:          1 TGGAGAAGCAGGGCACGTGCA
 22
                  ||||||||||||||||*|||**
Sbjct:        964 ACCTCTTCGTCCCGTGAACGAG 943
                           ||-----------------------------------------1
                           |------------------------------------------7

>miR164a-5p
    Score: 2.0  Deg: 7:984:205:1254T_00073806

Query:          1 TGGAGAAGCAGGGCACGTGCA
 22
                  ||||||||||||||||*|||**
Sbjct:        993 ACCTCTTCGTCCCGTGAACGAG 972
                           ||-----------------------------------------1
                           |------------------------------------------7

>miR164b-5p
    Score: 1.0  Deg: 124:1010:124:445  T_00052644

Query:          1 TGGAGAAGCAGGGCACGTGCA
 22
                  ||||||||||||||||||*||*
Sbjct:       1019 ACCTCTTCGTCCCGTGCAAGTA 998
                  ||   |  ||   || |  |--------------------------------1
                  ||   |  ||   || |-----------------------------------2
                  ||   |  ||   ||-------------------------------------1
                  ||   |  ||   |--------------------------------------1
                  ||   |  ||------------------------------------------124
                  ||   |  |-------------------------------------------4
                  ||   |----------------------------------------------1
                  ||--------------------------------------------------1
                  |---------------------------------------------------1
```

```
>miR164b-5p
    Score: 2.0  Deg: 32:832:32:113 T_00059787

Query:          1 TGGAGAAGCAGGGCACGTGCA
 22
                  |||||||:||||:|||||||**
Sbjct:        841 ACCTCTTTGTCCTGTGCACGAG 820
                           |       |----------------------------------1
                           |------------------------------------------32

>miR164b-5p
    Score: 2.0  Deg: 32:813:32:113 T_00059786

Query:          1 TGGAGAAGCAGGGCACGTGCA
 22
                  |||||||:||||:|||||||**
Sbjct:        822 ACCTCTTTGTCCTGTGCACGAG 801
                           |       |----------------------------------1
                           |------------------------------------------32

>miR164b-5p
    Score: 2.2  Deg: 7:928:7:192   T_00079376

Query:          1 TGGAGAAGCAGGGCACGTGCA
 22
                  :|||||||||||*||||||*|*
Sbjct:        937 GCCTCTTCGTCCAGTGCAC-TC 917
                           |       |----------------------------------1
                           |------------------------------------------7

>miR164b-5p
    Score: 2.0  Deg: 7:955:205:1253T_00073807

Query:          1 TGGAGAAGCAGGGCACGTGCA
 22
                  ||||||||||||||||*|||**
Sbjct:        964 ACCTCTTCGTCCCGTGAACGAG 943
                           ||-----------------------------------------1
                           |------------------------------------------7

>miR164b-5p
    Score: 2.0  Deg: 7:984:205:1254T_00073806

Query:          1 TGGAGAAGCAGGGCACGTGCA
 22
```

```
                  ||||||||||||||||*|||**
Sbjct:        993 ACCTCTTCGTCCCGTGAACGAG 972
                           ||------------------------------------------1
                           |-------------------------------------------7

>miR164c-5p
    Score: 1.0  Deg: 124:1010:124:445  T_00052644

Query:          1 TGGAGAAGCAGGGCACGTGCA
 22
                  |||||||||||||||||*||*
Sbjct:       1019 ACCTCTTCGTCCCGTGCAAGTA 998
                  ||   |  ||   || |  |---------------------------------1
                  ||   |  ||   || |------------------------------------2
                  ||   |  ||   ||--------------------------------------1
                  ||   |  ||   |---------------------------------------1
                  ||   |  ||-------------------------------------------124
                  ||   |  |--------------------------------------------4
                  ||   |-----------------------------------------------1
                  ||---------------------------------------------------1
                  |----------------------------------------------------1

>miR164c-5p
    Score: 2.0  Deg: 32:832:32:113 T_00059787

Query:          1 TGGAGAAGCAGGGCACGTGCA
 22
                  |||||||:||||:|||||||**
Sbjct:        841 ACCTCTTTGTCCTGTGCACGAG 820
                           |       |-----------------------------------1
                           |-------------------------------------------32

>miR164c-5p
    Score: 2.0  Deg: 32:813:32:113 T_00059786

Query:          1 TGGAGAAGCAGGGCACGTGCA
 22
                  |||||||:||||:|||||||**
Sbjct:        822 ACCTCTTTGTCCTGTGCACGAG 801
                           |       |-----------------------------------1
                           |-------------------------------------------32

>miR164c-5p
    Score: 2.2  Deg: 7:928:7:192   T_00079376
```

```
Query:          1 TGGAGAAGCAGGGCACGTGCA
 22
                  :|||||||||||*||||||*|*
Sbjct:        937 GCCTCTTCGTCCAGTGCAC-TC 917
                           |       |-----------------------------------1
                           |-------------------------------------------7

>miR164c-5p
    Score: 2.0  Deg: 7:955:205:1253T_00073807

Query:          1 TGGAGAAGCAGGGCACGTGCA
 22
                  ||||||||||||||||*|||**
Sbjct:        964 ACCTCTTCGTCCCGTGAACGAG 943
                           ||------------------------------------------1
                           |-------------------------------------------7

>miR164c-5p
    Score: 2.0  Deg: 7:984:205:1254T_00073806

Query:          1 TGGAGAAGCAGGGCACGTGCA
 22
                  ||||||||||||||||*|||**
Sbjct:        993 ACCTCTTCGTCCCGTGAACGAG 972
                           ||------------------------------------------1
                           |-------------------------------------------7

>miR164d-5p
    Score: 1.0  Deg: 124:1010:124:445  T_00052644

Query:          1 TGGAGAAGCAGGGCACGTGCA
 22
                  ||||||||||||||||||*||*
Sbjct:       1019 ACCTCTTCGTCCCGTGCAAGTA 998
                  ||   |  ||   || |  |---------------------------------1
                  ||   |  ||   || |------------------------------------2
                  ||   |  ||   ||--------------------------------------1
                  ||   |  ||   |---------------------------------------1
                  ||   |  ||-------------------------------------------124
                  ||   |  |--------------------------------------------4
                  ||   |-----------------------------------------------1
                  ||---------------------------------------------------1
                  |----------------------------------------------------1

>miR164d-5p
```

```
    Score: 2.0  Deg: 32:832:32:113 T_00059787

Query:           1 TGGAGAAGCAGGGCACGTGCA
 22
                   |||||||:||||:|||||||**
Sbjct:         841 ACCTCTTTGTCCTGTGCACGAG 820
                            |       |-----------------------------------1
                            |-------------------------------------------32

>miR164d-5p
    Score: 2.0  Deg: 32:813:32:113 T_00059786

Query:           1 TGGAGAAGCAGGGCACGTGCA
 22
                   |||||||:||||:|||||||**
Sbjct:         822 ACCTCTTTGTCCTGTGCACGAG 801
                            |       |-----------------------------------1
                            |-------------------------------------------32

>miR164d-5p
    Score: 2.2  Deg: 7:928:7:192   T_00079376

Query:           1 TGGAGAAGCAGGGCACGTGCA
 22
                   :|||||||||||*||||||*|*
Sbjct:         937 GCCTCTTCGTCCAGTGCAC-TC 917
                            |       |-----------------------------------1
                            |-------------------------------------------7

>miR164d-5p
    Score: 2.0  Deg: 7:955:205:1253T_00073807

Query:           1 TGGAGAAGCAGGGCACGTGCA
 22
                   ||||||||||||||||*|||**
Sbjct:         964 ACCTCTTCGTCCCGTGAACGAG 943
                            ||------------------------------------------1
                            |-------------------------------------------7

>miR164d-5p
    Score: 2.0  Deg: 7:984:205:1254T_00073806

Query:           1 TGGAGAAGCAGGGCACGTGCA
 22
                   ||||||||||||||||*|||**
```

```
Sbjct:        993 ACCTCTTCGTCCCGTGAACGAG 972
                           ||-----------------------------------------1
                           |------------------------------------------7

>miR164e-5p
    Score: 1.0  Deg: 124:1010:124:445  T_00052644

Query:          1 TGGAGAAGCAGGGCACGTGCA
 22
                  |||||||||||||||||*||*
Sbjct:       1019 ACCTCTTCGTCCCGTGCAAGTA 998
                  ||   |  ||   || |  |--------------------------------1
                  ||   |  ||   || |-----------------------------------2
                  ||   |  ||   ||-------------------------------------1
                  ||   |  ||   |--------------------------------------1
                  ||   |  ||------------------------------------------124
                  ||   |  |-------------------------------------------4
                  ||   |----------------------------------------------1
                  ||--------------------------------------------------1
                  |---------------------------------------------------1

>miR164e-5p
    Score: 2.0  Deg: 32:832:32:113 T_00059787

Query:          1 TGGAGAAGCAGGGCACGTGCA
 22
                  |||||||:||||:|||||||**
Sbjct:        841 ACCTCTTTGTCCTGTGCACGAG 820
                           |       |----------------------------------1
                           |------------------------------------------32

>miR164e-5p
    Score: 2.0  Deg: 32:813:32:113 T_00059786

Query:          1 TGGAGAAGCAGGGCACGTGCA
 22
                  |||||||:||||:|||||||**
Sbjct:        822 ACCTCTTTGTCCTGTGCACGAG 801
                           |       |----------------------------------1
                           |------------------------------------------32

>miR164e-5p
    Score: 2.2  Deg: 7:928:7:192   T_00079376

Query:          1 TGGAGAAGCAGGGCACGTGCA
```

```
 22
                 :|||||||||||*||||||*|*
Sbjct:        937 GCCTCTTCGTCCAGTGCAC-TC 917
                           |       |----------------------------------1
                           |------------------------------------------7

>miR164e-5p
    Score: 2.0  Deg: 7:955:205:1253T_00073807

Query:          1 TGGAGAAGCAGGGCACGTGCA
 22
                 ||||||||||||||||*|||**
Sbjct:        964 ACCTCTTCGTCCCGTGAACGAG 943
                           ||-----------------------------------------1
                           |------------------------------------------7

>miR164e-5p
    Score: 2.0  Deg: 7:984:205:1254T_00073806

Query:          1 TGGAGAAGCAGGGCACGTGCA
 22
                 ||||||||||||||||*|||**
Sbjct:        993 ACCTCTTCGTCCCGTGAACGAG 972
                           ||-----------------------------------------1
                           |------------------------------------------7

>miR166a-3p
    Score: 2.2  Deg: 168:1340:168:232  T_00088111

Query:          1 TCGGACCAGGCTTCATTCCCC
 22
                 :|||||||||||||||:||***
Sbjct:       1349 GGCCTGGTCCGAAGTAGGGTCC 1328
                          |||          |------------------------------1
                          |||-----------------------------------------2
                          ||------------------------------------------168
                          |-------------------------------------------2

>miR166a-3p
    Score: 2.2  Deg: 168:1211:168:231  T_00088109

Query:          1 TCGGACCAGGCTTCATTCCCC
 22
                 :|||||||||||||||:||***
Sbjct:       1220 GGCCTGGTCCGAAGTAGGGTCC 1199
```

```
                         |||          |------------------------------1
                         |||-----------------------------------------2
                         ||------------------------------------------168
                         |-------------------------------------------2

>miR166a-3p
    Score: 2.2  Deg: 168:1234:168:232  T_00088110

Query:          1 TCGGACCAGGCTTCATTCCCC
 22
                  :|||||||||||||||:||***
Sbjct:       1243 GGCCTGGTCCGAAGTAGGGTCC 1222
                         |||          |------------------------------1
                         |||-----------------------------------------2
                         ||------------------------------------------168
                         |-------------------------------------------2

>miR166a-3p
    Score: 2.2  Deg: 118:530:118:378   T_00058154

Query:          1 TCGGACCAGGCTTCATTCCCC
 22
                  :|||||||||||||||:||***
Sbjct:        539 GGCCTGGTCCGAAGTAGGGTTA 518
                         |||-----------------------------------------1
                         ||------------------------------------------118
                         |-------------------------------------------3

>miR166a-3p
    Score: 2.2  Deg: 118:569:118:461   T_00058151

Query:          1 TCGGACCAGGCTTCATTCCCC
 22
                  :|||||||||||||||:||***
Sbjct:        578 GGCCTGGTCCGAAGTAGGGTTA 557
                         |||-----------------------------------------1
                         ||------------------------------------------118
                         |-------------------------------------------3

>miR166a-3p
    Score: 2.2  Deg: 118:404:118:379   T_00058150

Query:          1 TCGGACCAGGCTTCATTCCCC
 22
                  :|||||||||||||||:||***
```

```
Sbjct:        413 GGCCTGGTCCGAAGTAGGGTTA 392
                          |||-----------------------------------------1
                          ||------------------------------------------118
                          |-------------------------------------------3

>miR166a-3p
    Score: 2.2  Deg: 118:569:118:379   T_00058152

Query:          1 TCGGACCAGGCTTCATTCCCC
 22
                  :|||||||||||||||:||***
Sbjct:        578 GGCCTGGTCCGAAGTAGGGTTA 557
                          |||-----------------------------------------1
                          ||------------------------------------------118
                          |-------------------------------------------3

>miR166a-3p
    Score: 2.2  Deg: 118:530:118:461   T_00058153

Query:          1 TCGGACCAGGCTTCATTCCCC
 22
                  :|||||||||||||||:||***
Sbjct:        539 GGCCTGGTCCGAAGTAGGGTTA 518
                          |||-----------------------------------------1
                          ||------------------------------------------118
                          |-------------------------------------------3

>miR166a-3p
    Score: 2.2  Deg: 118:404:118:461   T_00058149

Query:          1 TCGGACCAGGCTTCATTCCCC
 22
                  :|||||||||||||||:||***
Sbjct:        413 GGCCTGGTCCGAAGTAGGGTTA 392
                          |||-----------------------------------------1
                          ||------------------------------------------118
                          |-------------------------------------------3

>miR166a-3p
    Score: 2.2  Deg: 367:1463:631:8376 T_00069032

Query:          1 TCGGACCAGGCTTCATTCCCC
 22
                  :|||||||||||||||:||***
Sbjct:       1472 GGCCTGGTCCGAAGTAGGGTCC 1451
```

```
                    |   ||||          |------------------------------1
                    |   ||||------------------------------------------1
                    |   |||-------------------------------------------367
                    |   ||--------------------------------------------11
                    |   |---------------------------------------------1
                    |-------------------------------------------------2

>miR166a-3p
    Score: 2.2  Deg: 367:1457:631:8125 T_00069033

Query:          1 TCGGACCAGGCTTCATTCCCC
 22
                  :|||||||||||||||:||***
Sbjct:       1466 GGCCTGGTCCGAAGTAGGGTCC 1445
                    |   ||||          |------------------------------1
                    |   ||||------------------------------------------1
                    |   |||-------------------------------------------367
                    |   ||--------------------------------------------11
                    |   |---------------------------------------------1
                    |-------------------------------------------------2

>miR166b-3p
    Score: 2.2  Deg: 168:1340:168:232  T_00088111

Query:          1 TCGGACCAGGCTTCATTCCCC
 22
                  :|||||||||||||||:||***
Sbjct:       1349 GGCCTGGTCCGAAGTAGGGTCC 1328
                         |||          |------------------------------1
                         |||------------------------------------------2
                         ||-------------------------------------------168
                         |--------------------------------------------2

>miR166b-3p
    Score: 2.2  Deg: 168:1211:168:231  T_00088109

Query:          1 TCGGACCAGGCTTCATTCCCC
 22
                  :|||||||||||||||:||***
Sbjct:       1220 GGCCTGGTCCGAAGTAGGGTCC 1199
                         |||          |------------------------------1
                         |||------------------------------------------2
                         ||-------------------------------------------168
                         |--------------------------------------------2
```

```
>miR166b-3p
    Score: 2.2  Deg: 168:1234:168:232  T_00088110

Query:          1 TCGGACCAGGCTTCATTCCCC
 22
                  :|||||||||||||||:||***
Sbjct:       1243 GGCCTGGTCCGAAGTAGGGTCC 1222
                          |||         |------------------------------1
                          |||------------------------------------------2
                          ||-------------------------------------------168
                          |--------------------------------------------2

>miR166b-3p
    Score: 2.2  Deg: 118:530:118:378   T_00058154

Query:          1 TCGGACCAGGCTTCATTCCCC
 22
                  :|||||||||||||||:||***
Sbjct:        539 GGCCTGGTCCGAAGTAGGGTTA 518
                          |||------------------------------------------1
                          ||-------------------------------------------118
                          |--------------------------------------------3

>miR166b-3p
    Score: 2.2  Deg: 118:569:118:461   T_00058151

Query:          1 TCGGACCAGGCTTCATTCCCC
 22
                  :|||||||||||||||:||***
Sbjct:        578 GGCCTGGTCCGAAGTAGGGTTA 557
                          |||------------------------------------------1
                          ||-------------------------------------------118
                          |--------------------------------------------3

>miR166b-3p
    Score: 2.2  Deg: 118:404:118:379   T_00058150

Query:          1 TCGGACCAGGCTTCATTCCCC
 22
                  :|||||||||||||||:||***
Sbjct:        413 GGCCTGGTCCGAAGTAGGGTTA 392
                          |||------------------------------------------1
                          ||-------------------------------------------118
                          |--------------------------------------------3
```

```
>miR166b-3p
    Score: 2.2  Deg: 118:569:118:379   T_00058152

Query:           1 TCGGACCAGGCTTCATTCCCC
 22
                  :|||||||||||||||:||***
Sbjct:         578 GGCCTGGTCCGAAGTAGGGTTA 557
                          |||------------------------------------------1
                          ||-------------------------------------------118
                          |--------------------------------------------3

>miR166b-3p
    Score: 2.2  Deg: 118:530:118:461   T_00058153

Query:           1 TCGGACCAGGCTTCATTCCCC
 22
                  :|||||||||||||||:||***
Sbjct:         539 GGCCTGGTCCGAAGTAGGGTTA 518
                          |||------------------------------------------1
                          ||-------------------------------------------118
                          |--------------------------------------------3

>miR166b-3p
    Score: 2.2  Deg: 118:404:118:461   T_00058149

Query:           1 TCGGACCAGGCTTCATTCCCC
 22
                  :|||||||||||||||:||***
Sbjct:         413 GGCCTGGTCCGAAGTAGGGTTA 392
                          |||------------------------------------------1
                          ||-------------------------------------------118
                          |--------------------------------------------3

>miR166b-3p
    Score: 2.2  Deg: 367:1463:631:8376 T_00069032

Query:           1 TCGGACCAGGCTTCATTCCCC
 22
                  :|||||||||||||||:||***
Sbjct:        1472 GGCCTGGTCCGAAGTAGGGTCC 1451
                     |   ||||          |-------------------------------1
                     |   ||||------------------------------------------1
                     |   |||-------------------------------------------367
                     |   ||--------------------------------------------11
                     |   |---------------------------------------------1
```

```
                     |------------------------------------------------2

>miR166b-3p
    Score: 2.2  Deg: 367:1457:631:8125 T_00069033

Query:          1 TCGGACCAGGCTTCATTCCCC
 22
                  :|||||||||||||||:||***
Sbjct:       1466 GGCCTGGTCCGAAGTAGGGTCC 1445
                     |   ||||          |------------------------------1
                     |   ||||-----------------------------------------1
                     |   |||------------------------------------------367
                     |   ||-------------------------------------------11
                     |   |--------------------------------------------1
                     |------------------------------------------------2

>miR166c-3p
    Score: 2.2  Deg: 168:1340:168:232  T_00088111

Query:          1 TCGGACCAGGCTTCATTCCCC
 22
                  :|||||||||||||||:||***
Sbjct:       1349 GGCCTGGTCCGAAGTAGGGTCC 1328
                          |||          |------------------------------1
                          |||-----------------------------------------2
                          ||------------------------------------------168
                          |-------------------------------------------2

>miR166c-3p
    Score: 2.2  Deg: 168:1211:168:231  T_00088109

Query:          1 TCGGACCAGGCTTCATTCCCC
 22
                  :|||||||||||||||:||***
Sbjct:       1220 GGCCTGGTCCGAAGTAGGGTCC 1199
                          |||          |------------------------------1
                          |||-----------------------------------------2
                          ||------------------------------------------168
                          |-------------------------------------------2

>miR166c-3p
    Score: 2.2  Deg: 168:1234:168:232  T_00088110

Query:          1 TCGGACCAGGCTTCATTCCCC
 22
```

```
                   :|||||||||||||||:||***
Sbjct:       1243 GGCCTGGTCCGAAGTAGGGTCC 1222
                           |||          |-------------------------------1
                           |||------------------------------------------2
                           ||-------------------------------------------168
                           |--------------------------------------------2
```

```
>miR166c-3p
    Score: 2.2  Deg: 118:530:118:378   T_00058154

Query:          1 TCGGACCAGGCTTCATTCCCC
 22
                   :|||||||||||||||:||***
Sbjct:        539 GGCCTGGTCCGAAGTAGGGTTA 518
                           |||------------------------------------------1
                           ||-------------------------------------------118
                           |--------------------------------------------3
```

```
>miR166c-3p
    Score: 2.2  Deg: 118:569:118:461   T_00058151

Query:          1 TCGGACCAGGCTTCATTCCCC
 22
                   :|||||||||||||||:||***
Sbjct:        578 GGCCTGGTCCGAAGTAGGGTTA 557
                           |||------------------------------------------1
                           ||-------------------------------------------118
                           |--------------------------------------------3
```

```
>miR166c-3p
    Score: 2.2  Deg: 118:404:118:379   T_00058150

Query:          1 TCGGACCAGGCTTCATTCCCC
 22
                   :|||||||||||||||:||***
Sbjct:        413 GGCCTGGTCCGAAGTAGGGTTA 392
                           |||------------------------------------------1
                           ||-------------------------------------------118
                           |--------------------------------------------3
```

```
>miR166c-3p
    Score: 2.2  Deg: 118:569:118:379   T_00058152

Query:          1 TCGGACCAGGCTTCATTCCCC
 22
```

```
                  :|||||||||||||||:||***
Sbjct:        578 GGCCTGGTCCGAAGTAGGGTTA 557
                          |||------------------------------------------1
                          ||-------------------------------------------118
                          |--------------------------------------------3

>miR166c-3p
    Score: 2.2  Deg: 118:530:118:461   T_00058153

Query:          1 TCGGACCAGGCTTCATTCCCC
 22
                  :|||||||||||||||:||***
Sbjct:        539 GGCCTGGTCCGAAGTAGGGTTA 518
                          |||------------------------------------------1
                          ||-------------------------------------------118
                          |--------------------------------------------3

>miR166c-3p
    Score: 2.2  Deg: 118:404:118:461   T_00058149

Query:          1 TCGGACCAGGCTTCATTCCCC
 22
                  :|||||||||||||||:||***
Sbjct:        413 GGCCTGGTCCGAAGTAGGGTTA 392
                          |||------------------------------------------1
                          ||-------------------------------------------118
                          |--------------------------------------------3

>miR166c-3p
    Score: 2.2  Deg: 367:1463:631:8376 T_00069032

Query:          1 TCGGACCAGGCTTCATTCCCC
 22
                  :|||||||||||||||:||***
Sbjct:       1472 GGCCTGGTCCGAAGTAGGGTCC 1451
                     |   ||||          |-------------------------------1
                     |   ||||------------------------------------------1
                     |   |||-------------------------------------------367
                     |   ||--------------------------------------------11
                     |   |---------------------------------------------1
                     |-------------------------------------------------2

>miR166c-3p
    Score: 2.2  Deg: 367:1457:631:8125 T_00069033
```

```
Query:           1 TCGGACCAGGCTTCATTCCCC
 22
                   :|||||||||||||||:||***
Sbjct:        1466 GGCCTGGTCCGAAGTAGGGTCC 1445
                      |   ||||          |-------------------------------1
                      |   ||||------------------------------------------1
                      |   |||-------------------------------------------367
                      |   ||--------------------------------------------11
                      |   |---------------------------------------------1
                      |-------------------------------------------------2


>miR169c
    Score: 2.5  Deg: 19:1476:19:150T_00065068

Query:           1 TAGCCAAGGATGACTTGCCTG
 22
                   *|||||||:||||*|||||||*
Sbjct:        1485 TTCGGTTCTTACTTAACGGACT 1464
                        |   |-------------------------------------------19
                        |-----------------------------------------------2

>miR169c
    Score: 2.5  Deg: 19:1902:19:159T_00065067

Query:           1 TAGCCAAGGATGACTTGCCTG
 22
                   *|||||||:||||*|||||||*
Sbjct:        1911 TTCGGTTCTTACTTAACGGACT 1890
                        |   |-------------------------------------------19
                        |-----------------------------------------------2

>miR169c
    Score: 2.5  Deg: 14:1593:33:498T_00083345

Query:           1 TAGCCAAGGATGACTTGCCTG
 22
                   *|||||||||||||||*||::*
Sbjct:        1602 CTCGGTTCCTACTGAA-GGGTA 1582
                      |  ||||          ||------------------------------2
                      |  ||||          |-------------------------------2
                      |  ||||-------------------------------------------14
                      |  |||--------------------------------------------3
                      |  ||---------------------------------------------2
                      |  |----------------------------------------------1
```

```
                    |-----------------------------------------------1

>miR169c
    Score: 2.5  Deg: 14:1577:33:498T_00083347

Query:          1 TAGCCAAGGATGACTTGCCTG
 22
                  *|||||||||||||||*||::*
Sbjct:       1586 CTCGGTTCCTACTGAA-GGGTA 1566
                    |  ||||          ||-------------------------------2
                    |  ||||          |--------------------------------2
                    |  ||||-----------------------------------------14
                    |  |||------------------------------------------3
                    |  ||-------------------------------------------2
                    |  |--------------------------------------------1
                    |-----------------------------------------------1

>miR169c
    Score: 2.5  Deg: 14:1544:33:498T_00083343

Query:          1 TAGCCAAGGATGACTTGCCTG
 22
                  *|||||||||||||||*||::*
Sbjct:       1553 CTCGGTTCCTACTGAA-GGGTA 1533
                    |  ||||          ||-------------------------------2
                    |  ||||          |--------------------------------2
                    |  ||||-----------------------------------------14
                    |  |||------------------------------------------3
                    |  ||-------------------------------------------2
                    |  |--------------------------------------------1
                    |-----------------------------------------------1

>miR169c
    Score: 2.5  Deg: 14:1523:33:497T_00083346

Query:          1 TAGCCAAGGATGACTTGCCTG
 22
                  *|||||||||||||||*||::*
Sbjct:       1532 CTCGGTTCCTACTGAA-GGGTA 1512
                    |  ||||          ||-------------------------------2
                    |  ||||          |--------------------------------2
                    |  ||||-----------------------------------------14
                    |  |||------------------------------------------3
                    |  ||-------------------------------------------2
                    |  |--------------------------------------------1
```

```
                    |----------------------------------------------1

>miR169c
    Score: 2.5  Deg: 14:1538:33:497T_00083344

Query:          1 TAGCCAAGGATGACTTGCCTG
 22
                  *|||||||||||||||*||::*
Sbjct:       1547 CTCGGTTCCTACTGAA-GGGTA 1527
                     |  ||||         ||-----------------------------2
                     |  ||||         |------------------------------2
                     |  ||||----------------------------------------14
                     |  |||-----------------------------------------3
                     |  ||------------------------------------------2
                     |  |-------------------------------------------1
                     |----------------------------------------------1

>miR169c
    Score: 2.5  Deg: 14:1161:14:305T_00083342

Query:          1 TAGCCAAGGATGACTTGCCTG
 22
                  *|||||||||||||||*||::*
Sbjct:       1170 CTCGGTTCCTACTGAA-GGGTA 1150
                     |  ||||         ||-----------------------------2
                     |  ||||         |------------------------------2
                     |  ||||----------------------------------------14
                     |  |||-----------------------------------------3
                     |  ||------------------------------------------2
                     |  |-------------------------------------------1
                     |----------------------------------------------1

>miR169d
    Score: 2.5  Deg: 19:1476:19:150T_00065068

Query:          1 TAGCCAAGGATGACTTGCCTG
 22
                  *|||||||:||||*|||||||*
Sbjct:       1485 TTCGGTTCTTACTTAACGGACT 1464
                       |   |----------------------------------------19
                       |--------------------------------------------2

>miR169d
    Score: 2.5  Deg: 19:1902:19:159T_00065067
```

```
Query:          1 TAGCCAAGGATGACTTGCCTG
 22
                  *|||||||:||||*|||||||*
Sbjct:       1911 TTCGGTTCTTACTTAACGGACT 1890
                      |   |------------------------------------------19
                      |----------------------------------------------2

>miR169d
    Score: 2.5  Deg: 14:1593:33:498T_00083345

Query:          1 TAGCCAAGGATGACTTGCCTG
 22
                  *|||||||||||||||*||::*
Sbjct:       1602 CTCGGTTCCTACTGAA-GGGTA 1582
                    |  ||||          ||-------------------------------2
                    |  ||||          |--------------------------------2
                    |  ||||-------------------------------------------14
                    |  |||--------------------------------------------3
                    |  ||---------------------------------------------2
                    |  |----------------------------------------------1
                    |-------------------------------------------------1

>miR169d
    Score: 2.5  Deg: 14:1577:33:498T_00083347

Query:          1 TAGCCAAGGATGACTTGCCTG
 22
                  *|||||||||||||||*||::*
Sbjct:       1586 CTCGGTTCCTACTGAA-GGGTA 1566
                    |  ||||          ||-------------------------------2
                    |  ||||          |--------------------------------2
                    |  ||||-------------------------------------------14
                    |  |||--------------------------------------------3
                    |  ||---------------------------------------------2
                    |  |----------------------------------------------1
                    |-------------------------------------------------1

>miR169d
    Score: 2.5  Deg: 14:1544:33:498T_00083343

Query:          1 TAGCCAAGGATGACTTGCCTG
 22
                  *|||||||||||||||*||::*
Sbjct:       1553 CTCGGTTCCTACTGAA-GGGTA 1533
                    |  ||||          ||-------------------------------2
```

```
                     |  ||||          |--------------------------------2
                     |  ||||-------------------------------------------14
                     |  |||--------------------------------------------3
                     |  ||---------------------------------------------2
                     |  |----------------------------------------------1
                     |-------------------------------------------------1

>miR169d
    Score: 2.5  Deg: 14:1523:33:497T_00083346

Query:          1 TAGCCAAGGATGACTTGCCTG
 22
                  *||||||||||||||*||::*
Sbjct:       1532 CTCGGTTCCTACTGAA-GGGTA 1512
                     |  ||||          ||-------------------------------2
                     |  ||||          |--------------------------------2
                     |  ||||-------------------------------------------14
                     |  |||--------------------------------------------3
                     |  ||---------------------------------------------2
                     |  |----------------------------------------------1
                     |-------------------------------------------------1

>miR169d
    Score: 2.5  Deg: 14:1538:33:497T_00083344

Query:          1 TAGCCAAGGATGACTTGCCTG
 22
                  *||||||||||||||*||::*
Sbjct:       1547 CTCGGTTCCTACTGAA-GGGTA 1527
                     |  ||||          ||-------------------------------2
                     |  ||||          |--------------------------------2
                     |  ||||-------------------------------------------14
                     |  |||--------------------------------------------3
                     |  ||---------------------------------------------2
                     |  |----------------------------------------------1
                     |-------------------------------------------------1

>miR169d
    Score: 2.5  Deg: 14:1161:14:305T_00083342

Query:          1 TAGCCAAGGATGACTTGCCTG
 22
                  *||||||||||||||*||::*
Sbjct:       1170 CTCGGTTCCTACTGAA-GGGTA 1150
                     |  ||||          ||-------------------------------2
```

```
                     |  ||||          |--------------------------------2
                     |  ||||-----------------------------------------14
                     |  |||------------------------------------------3
                     |  ||-------------------------------------------2
                     |  |--------------------------------------------1
                     |-----------------------------------------------1


>miR172a-3p
    Score: 1.5  Deg: 9:748:12:67   T_00059075

Query:          1 AGAATCTTGATGATGCTGC-AT
 22
                  ||||||:||||||||||||*||*
Sbjct:        757 TCTTAGGACTACTACGACGTTAC 735
                           |         |----------------------------------1
                           |--------------------------------------------9

>miR172a-3p
    Score: 1.5  Deg: 9:792:12:67   T_00059076

Query:          1 AGAATCTTGATGATGCTGC-AT
 22
                  ||||||:||||||||||||*||*
Sbjct:        801 TCTTAGGACTACTACGACGTTAC 779
                           |         |----------------------------------1
                           |--------------------------------------------9

>miR172a-3p
    Score: 1.5  Deg: 621:1710:621:1460 T_00087826

Query:          1 AGAATCTTGATGATGCTGCA-T
 22
                  ||||||:|||||||||||||*|*
Sbjct:       1719 TCTTAGGACTACTACGACGTCAT 1697
                   | | |   || |       ||---------------------------------1
                   | | |   || |       |----------------------------------1
                   | | |   || |------------------------------------------1
                   | | |   ||--------------------------------------------621
                   | | |   |---------------------------------------------2
                   | | |-------------------------------------------------1
                   | |---------------------------------------------------2
                   |-----------------------------------------------------1

>miR172a-3p
```

```
    Score: 1.5  Deg: 621:1851:621:1456 T_00087824

Query:           1 AGAATCTTGATGATGCTGCA-T
 22
                   ||||||:|||||||||||||*|*
Sbjct:        1860 TCTTAGGACTACTACGACGTCAT 1838
                    | | |  || |       ||---------------------------------1
                    | | |  || |       |----------------------------------1
                    | | |  || |------------------------------------------1
                    | | |  ||--------------------------------------------621
                    | | |  |---------------------------------------------2
                    | | |------------------------------------------------1
                    | |--------------------------------------------------2
                    |----------------------------------------------------1

>miR172a-3p
    Score: 1.5  Deg: 621:734:621:1156  T_00087822

Query:           1 AGAATCTTGATGATGCTGCA-T
 22
                   ||||||:|||||||||||||*|*
Sbjct:         743 TCTTAGGACTACTACGACGTCAT 721
                    | | |  || |       ||---------------------------------1
                    | | |  || |       |----------------------------------1
                    | | |  || |------------------------------------------1
                    | | |  ||--------------------------------------------621
                    | | |  |---------------------------------------------2
                    | | |------------------------------------------------1
                    | |--------------------------------------------------2
                    |----------------------------------------------------1

>miR172a-3p
    Score: 1.5  Deg: 621:2169:621:1460 T_00087823

Query:           1 AGAATCTTGATGATGCTGCA-T
 22
                   ||||||:|||||||||||||*|*
Sbjct:        2178 TCTTAGGACTACTACGACGTCAT 2156
                    | | |  || |       ||---------------------------------1
                    | | |  || |       |----------------------------------1
                    | | |  || |------------------------------------------1
                    | | |  ||--------------------------------------------621
                    | | |  |---------------------------------------------2
                    | | |------------------------------------------------1
                    | |--------------------------------------------------2
```

```
                   |---------------------------------------------------1

>miR172a-3p
    Score: 1.5  Deg: 621:1808:621:1455 T_00087825

Query:          1 AGAATCTTGATGATGCTGCA-T
 22
                  ||||||:|||||||||||||*|*
Sbjct:       1817 TCTTAGGACTACTACGACGTCAT 1795
                   | | |  || |       ||--------------------------------1
                   | | |  || |       |---------------------------------1
                   | | |  || |-----------------------------------------1
                   | | |  ||-------------------------------------------621
                   | | |  |--------------------------------------------2
                   | | |-----------------------------------------------1
                   | |-------------------------------------------------2
                   |---------------------------------------------------1

>miR172a-3p
    Score: 1.5  Deg: 621:2219:621:1466 T_00087827

Query:          1 AGAATCTTGATGATGCTGCA-T
 22
                  ||||||:|||||||||||||*|*
Sbjct:       2228 TCTTAGGACTACTACGACGTCAT 2206
                   | | |  || |       ||--------------------------------1
                   | | |  || |       |---------------------------------1
                   | | |  || |-----------------------------------------1
                   | | |  ||-------------------------------------------621
                   | | |  |--------------------------------------------2
                   | | |-----------------------------------------------1
                   | |-------------------------------------------------2
                   |---------------------------------------------------1

>miR172a-3p
    Score: 1.5  Deg: 621:2130:621:1456 T_00087828

Query:          1 AGAATCTTGATGATGCTGCA-T
 22
                  ||||||:|||||||||||||*|*
Sbjct:       2139 TCTTAGGACTACTACGACGTCAT 2117
                   | | |  || |       ||--------------------------------1
                   | | |  || |       |---------------------------------1
                   | | |  || |-----------------------------------------1
                   | | |  ||-------------------------------------------621
```

```
                   | | |   |---------------------------------------------2
                   | | |------------------------------------------------1
                   | |--------------------------------------------------2
                   |----------------------------------------------------1

>miR172a-3p
    Score: 1.5  Deg: 9:561:17:50   T_00081649

Query:          1 AGAATCTTGATGATGCTGC-AT
 22
                  ||||||:||||||||||||*||*
Sbjct:        570 TCTTAGGACTACTACGACGTTAC 548
                           |         |-----------------------------------1
                           |---------------------------------------------9

>miR172a-3p
    Score: 2.0  Deg: 7:2392:205:1270   T_00014183

Query:          1 AGAATCTTGATGATGCTGCAT
 22
                  *|||||:|||||||||||||**
Sbjct:       2401 CCTTAGGACTACTACGACGTCG 2380
                           |   |      |--------------------------------1
                           |   |---------------------------------------1
                           |-------------------------------------------7

>miR172a-3p
    Score: 2.0  Deg: 7:2240:205:1269   T_00014181

Query:          1 AGAATCTTGATGATGCTGCAT
 22
                  *|||||:|||||||||||||**
Sbjct:       2249 CCTTAGGACTACTACGACGTCG 2228
                           |   |      |--------------------------------1
                           |   |---------------------------------------1
                           |-------------------------------------------7

>miR172a-3p
    Score: 2.0  Deg: 7:2189:205:1265   T_00014179

Query:          1 AGAATCTTGATGATGCTGCAT
 22
                  *|||||:|||||||||||||**
Sbjct:       2198 CCTTAGGACTACTACGACGTCG 2177
                           |   |      |--------------------------------1
```

```
                           |    |--------------------------------------1
                           |-------------------------------------------7

>miR172a-3p
    Score: 2.0  Deg: 7:1352:28:148 T_00014174

Query:          1 AGAATCTTGATGATGCTGCAT
 22
                  *|||||:|||||||||||||**
Sbjct:       1361 CCTTAGGACTACTACGACGTCG 1340
                           |    |      |-------------------------------1
                           |    |--------------------------------------1
                           |-------------------------------------------7

>miR172a-3p
    Score: 2.0  Deg: 7:2105:205:1265   T_00014178

Query:          1 AGAATCTTGATGATGCTGCAT
 22
                  *|||||:|||||||||||||**
Sbjct:       2114 CCTTAGGACTACTACGACGTCG 2093
                           |    |      |-------------------------------1
                           |    |--------------------------------------1
                           |-------------------------------------------7

>miR172a-3p
    Score: 2.0  Deg: 7:1349:28:146 T_00014175

Query:          1 AGAATCTTGATGATGCTGCAT
 22
                  *|||||:|||||||||||||**
Sbjct:       1358 CCTTAGGACTACTACGACGTCG 1337
                           |    |      |-------------------------------1
                           |    |--------------------------------------1
                           |-------------------------------------------7

>miR172a-3p
    Score: 2.0  Deg: 7:2420:205:1272   T_00014182

Query:          1 AGAATCTTGATGATGCTGCAT
 22
                  *|||||:|||||||||||||**
Sbjct:       2429 CCTTAGGACTACTACGACGTCG 2408
                           |    |      |-------------------------------1
                           |    |--------------------------------------1
```

```
                           |-----------------------------------------7

>miR172a-3p
    Score: 2.0  Deg: 7:2159:205:1265   T_00014177

Query:          1 AGAATCTTGATGATGCTGCAT
 22
                  *|||||:|||||||||||||**
Sbjct:       2168 CCTTAGGACTACTACGACGTCG 2147
                           |   |      |--------------------------------1
                           |   |-------------------------------------1
                           |-----------------------------------------7

>miR172a-3p
    Score: 2.0  Deg: 7:1349:28:146 T_00014176

Query:          1 AGAATCTTGATGATGCTGCAT
 22
                  *|||||:|||||||||||||**
Sbjct:       1358 CCTTAGGACTACTACGACGTCG 1337
                           |   |      |--------------------------------1
                           |   |-------------------------------------1
                           |-----------------------------------------7

>miR172a-3p
    Score: 2.0  Deg: 7:2235:205:1267   T_00014180

Query:          1 AGAATCTTGATGATGCTGCAT
 22
                  *|||||:|||||||||||||**
Sbjct:       2244 CCTTAGGACTACTACGACGTCG 2223
                           |   |      |--------------------------------1
                           |   |-------------------------------------1
                           |-----------------------------------------7

>miR172a-3p
    Score: 2.0  Deg: 7:2485:205:1273   T_00014186

Query:          1 AGAATCTTGATGATGCTGCAT
 22
                  *|||||:|||||||||||||**
Sbjct:       2494 CCTTAGGACTACTACGACGTCG 2473
                           |   |      |--------------------------------1
                           |   |-------------------------------------1
                           |-----------------------------------------7
```

```
>miR172a-3p
    Score: 2.0  Deg: 7:2415:205:1270   T_00014185

Query:          1 AGAATCTTGATGATGCTGCAT
 22
                  *|||||:|||||||||||||**
Sbjct:       2424 CCTTAGGACTACTACGACGTCG 2403
                           |   |      |--------------------------------1
                           |   |---------------------------------------1
                           |-------------------------------------------7

>miR172a-3p
    Score: 2.0  Deg: 7:2418:205:1272   T_00014184

Query:          1 AGAATCTTGATGATGCTGCAT
 22
                  *|||||:|||||||||||||**
Sbjct:       2427 CCTTAGGACTACTACGACGTCG 2406
                           |   |      |--------------------------------1
                           |   |---------------------------------------1
                           |-------------------------------------------7

>miR172b-3p
    Score: 1.5  Deg: 9:748:12:67   T_00059075

Query:          1 AGAATCTTGATGATGCTGTAG
 22
                  ||||||:|||||||||||:|:*
Sbjct:        757 TCTTAGGACTACTACGACGTTA 736
                           |         |---------------------------------1
                           |-------------------------------------------9

>miR172b-3p
    Score: 1.5  Deg: 9:792:12:67   T_00059076

Query:          1 AGAATCTTGATGATGCTGTAG
 22
                  ||||||:|||||||||||:|:*
Sbjct:        801 TCTTAGGACTACTACGACGTTA 780
                           |         |---------------------------------1
                           |-------------------------------------------9

>miR172b-3p
    Score: 1.2  Deg: 621:1710:621:1460 T_00087826
```

```
Query:          1 AGAATCTTGATGATGCTGTAG
 22
                  ||||||:|||||||||||:||*
Sbjct:       1719 TCTTAGGACTACTACGACGTCA 1698
                   | | |  || |       ||--------------------------------1
                   | | |  || |       |---------------------------------1
                   | | |  || |-----------------------------------------1
                   | | |  ||-------------------------------------------621
                   | | |  |--------------------------------------------2
                   | | |-----------------------------------------------1
                   | |-------------------------------------------------2
                   |---------------------------------------------------1

>miR172b-3p
    Score: 1.2  Deg: 621:1851:621:1456 T_00087824

Query:          1 AGAATCTTGATGATGCTGTAG
 22
                  ||||||:|||||||||||:||*
Sbjct:       1860 TCTTAGGACTACTACGACGTCA 1839
                   | | |  || |       ||--------------------------------1
                   | | |  || |       |---------------------------------1
                   | | |  || |-----------------------------------------1
                   | | |  ||-------------------------------------------621
                   | | |  |--------------------------------------------2
                   | | |-----------------------------------------------1
                   | |-------------------------------------------------2
                   |---------------------------------------------------1

>miR172b-3p
    Score: 1.2  Deg: 621:734:621:1156  T_00087822

Query:          1 AGAATCTTGATGATGCTGTAG
 22
                  ||||||:|||||||||||:||*
Sbjct:        743 TCTTAGGACTACTACGACGTCA 722
                   | | |  || |       ||--------------------------------1
                   | | |  || |       |---------------------------------1
                   | | |  || |-----------------------------------------1
                   | | |  ||-------------------------------------------621
                   | | |  |--------------------------------------------2
                   | | |-----------------------------------------------1
                   | |-------------------------------------------------2
                   |---------------------------------------------------1
```

```
>miR172b-3p
    Score: 1.2  Deg: 621:2169:621:1460 T_00087823

Query:           1 AGAATCTTGATGATGCTGTAG
 22
                   ||||||:|||||||||||:||*
Sbjct:        2178 TCTTAGGACTACTACGACGTCA 2157
                    | | |  || |       ||--------------------------------1
                    | | |  || |       |---------------------------------1
                    | | |  || |-----------------------------------------1
                    | | |  ||-------------------------------------------621
                    | | |  |--------------------------------------------2
                    | | |-----------------------------------------------1
                    | |-------------------------------------------------2
                    |---------------------------------------------------1

>miR172b-3p
    Score: 1.2  Deg: 621:1808:621:1455 T_00087825

Query:           1 AGAATCTTGATGATGCTGTAG
 22
                   ||||||:|||||||||||:||*
Sbjct:        1817 TCTTAGGACTACTACGACGTCA 1796
                    | | |  || |       ||--------------------------------1
                    | | |  || |       |---------------------------------1
                    | | |  || |-----------------------------------------1
                    | | |  ||-------------------------------------------621
                    | | |  |--------------------------------------------2
                    | | |-----------------------------------------------1
                    | |-------------------------------------------------2
                    |---------------------------------------------------1

>miR172b-3p
    Score: 1.2  Deg: 621:2219:621:1466 T_00087827

Query:           1 AGAATCTTGATGATGCTGTAG
 22
                   ||||||:|||||||||||:||*
Sbjct:        2228 TCTTAGGACTACTACGACGTCA 2207
                    | | |  || |       ||--------------------------------1
                    | | |  || |       |---------------------------------1
                    | | |  || |-----------------------------------------1
                    | | |  ||-------------------------------------------621
                    | | |  |--------------------------------------------2
```

```
                   | | |-----------------------------------------------1
                   | |-------------------------------------------------2
                   |---------------------------------------------------1

>miR172b-3p
    Score: 1.2  Deg: 621:2130:621:1456 T_00087828

Query:          1 AGAATCTTGATGATGCTGTAG
 22
                  ||||||:|||||||||||:||*
Sbjct:       2139 TCTTAGGACTACTACGACGTCA 2118
                   | | |  || |       ||--------------------------------1
                   | | |  || |       |---------------------------------1
                   | | |  || |-----------------------------------------1
                   | | |  ||-------------------------------------------621
                   | | |  |--------------------------------------------2
                   | | |-----------------------------------------------1
                   | |-------------------------------------------------2
                   |---------------------------------------------------1

>miR172b-3p
    Score: 1.5  Deg: 9:561:17:50   T_00081649

Query:          1 AGAATCTTGATGATGCTGTAG
 22
                  ||||||:|||||||||||:|:*
Sbjct:        570 TCTTAGGACTACTACGACGTTA 549
                           |         |---------------------------------1
                           |-------------------------------------------9

>miR172b-3p
    Score: 1.8  Deg: 7:2392:205:1270   T_00014183

Query:          1 AGAATCTTGATGATGCTGTAG
 22
                  *|||||:|||||||||||:||*
Sbjct:       2401 CCTTAGGACTACTACGACGTCG 2380
                           |   |      |--------------------------------1
                           |   |---------------------------------------1
                           |-------------------------------------------7

>miR172b-3p
    Score: 1.8  Deg: 7:2240:205:1269   T_00014181

Query:          1 AGAATCTTGATGATGCTGTAG
```

```
 22
                 *|||||:|||||||||||:||*
Sbjct:       2249 CCTTAGGACTACTACGACGTCG 2228
                           |   |      |--------------------------------1
                           |   |---------------------------------------1
                           |-------------------------------------------7

>miR172b-3p
    Score: 1.8  Deg: 7:2189:205:1265   T_00014179

Query:          1 AGAATCTTGATGATGCTGTAG
 22
                 *|||||:|||||||||||:||*
Sbjct:       2198 CCTTAGGACTACTACGACGTCG 2177
                           |   |      |--------------------------------1
                           |   |---------------------------------------1
                           |-------------------------------------------7

>miR172b-3p
    Score: 1.8  Deg: 7:1352:28:148 T_00014174

Query:          1 AGAATCTTGATGATGCTGTAG
 22
                 *|||||:|||||||||||:||*
Sbjct:       1361 CCTTAGGACTACTACGACGTCG 1340
                           |   |      |--------------------------------1
                           |   |---------------------------------------1
                           |-------------------------------------------7

>miR172b-3p
    Score: 1.8  Deg: 7:2105:205:1265   T_00014178

Query:          1 AGAATCTTGATGATGCTGTAG
 22
                 *|||||:|||||||||||:||*
Sbjct:       2114 CCTTAGGACTACTACGACGTCG 2093
                           |   |      |--------------------------------1
                           |   |---------------------------------------1
                           |-------------------------------------------7

>miR172b-3p
    Score: 1.8  Deg: 7:1349:28:146 T_00014175

Query:          1 AGAATCTTGATGATGCTGTAG
 22
```

```
                  *|||||:|||||||||||:||*
Sbjct:       1358 CCTTAGGACTACTACGACGTCG 1337
                           |   |      |--------------------------------1
                           |   |---------------------------------------1
                           |-------------------------------------------7

>miR172b-3p
    Score: 1.8  Deg: 7:2420:205:1272   T_00014182

Query:          1 AGAATCTTGATGATGCTGTAG
 22
                  *|||||:|||||||||||:||*
Sbjct:       2429 CCTTAGGACTACTACGACGTCG 2408
                           |   |      |--------------------------------1
                           |   |---------------------------------------1
                           |-------------------------------------------7

>miR172b-3p
    Score: 1.8  Deg: 7:2159:205:1265   T_00014177

Query:          1 AGAATCTTGATGATGCTGTAG
 22
                  *|||||:|||||||||||:||*
Sbjct:       2168 CCTTAGGACTACTACGACGTCG 2147
                           |   |      |--------------------------------1
                           |   |---------------------------------------1
                           |-------------------------------------------7

>miR172b-3p
    Score: 1.8  Deg: 7:1349:28:146 T_00014176

Query:          1 AGAATCTTGATGATGCTGTAG
 22
                  *|||||:|||||||||||:||*
Sbjct:       1358 CCTTAGGACTACTACGACGTCG 1337
                           |   |      |--------------------------------1
                           |   |---------------------------------------1
                           |-------------------------------------------7

>miR172b-3p
    Score: 1.8  Deg: 7:2235:205:1267   T_00014180

Query:          1 AGAATCTTGATGATGCTGTAG
 22
                  *|||||:|||||||||||:||*
```

```
Sbjct:       2244 CCTTAGGACTACTACGACGTCG 2223
                           |   |      |--------------------------------1
                           |   |---------------------------------------1
                           |-------------------------------------------7

>miR172b-3p
    Score: 1.8  Deg: 7:2485:205:1273   T_00014186

Query:          1 AGAATCTTGATGATGCTGTAG
 22
                  *|||||:|||||||||||:||*
Sbjct:       2494 CCTTAGGACTACTACGACGTCG 2473
                           |   |      |--------------------------------1
                           |   |---------------------------------------1
                           |-------------------------------------------7

>miR172b-3p
    Score: 1.8  Deg: 7:2415:205:1270   T_00014185

Query:          1 AGAATCTTGATGATGCTGTAG
 22
                  *|||||:|||||||||||:||*
Sbjct:       2424 CCTTAGGACTACTACGACGTCG 2403
                           |   |      |--------------------------------1
                           |   |---------------------------------------1
                           |-------------------------------------------7

>miR172b-3p
    Score: 1.8  Deg: 7:2418:205:1272   T_00014184

Query:          1 AGAATCTTGATGATGCTGTAG
 22
                  *|||||:|||||||||||:||*
Sbjct:       2427 CCTTAGGACTACTACGACGTCG 2406
                           |   |      |--------------------------------1
                           |   |---------------------------------------1
                           |-------------------------------------------7

>miR393a-5p
    Score: 1.5  Deg: 109:1993:631:3423 T_00032843

Query:          1 TCCAAAGGGATCGCATTG-ATCC
 23
                  |||||||||||||||||**|||*
Sbjct:       2002 AGGTTTCCCTAGCGTAACAGAGGT 1979
```

```
                     |||  |  ||   ||--------------------------------2
                     |||  |  ||   |---------------------------------1
                     |||  |  ||-------------------------------------2
                     |||  |  |--------------------------------------2
                     |||  |-----------------------------------------1
                     |||--------------------------------------------109
                     ||---------------------------------------------5
                     |----------------------------------------------1
```

>miR393a-5p
Score: 1.5 Deg: 222:1064:222:1728 T_00015134

```
Query:          1 TCCAAAGGGATCGCATTGATCC
 23
                  |||||||||||||||||*|*|*
Sbjct:       1073 AGGTTTCCCTAGCGTAACAAAGT 1051
                   ||| | ||||||||||   | |-------------------------------1
                   ||| | ||||||||||   |---------------------------------1
                   ||| | ||||||||||-------------------------------------2
                   ||| | |||||||||--------------------------------------3
                   ||| | ||||||||---------------------------------------2
                   ||| | |||||||----------------------------------------3
                   ||| | ||||||-----------------------------------------1
                   ||| | |||||------------------------------------------1
                   ||| | ||||-------------------------------------------1
                   ||| | |||--------------------------------------------222
                   ||| | ||---------------------------------------------1
                   ||| | |----------------------------------------------3
                   ||| |------------------------------------------------1
                   |||--------------------------------------------------1
                   ||---------------------------------------------------1
                   |----------------------------------------------------2
```

>miR393b-5p
Score: 1.5 Deg: 109:1993:631:3423 T_00032843

```
Query:          1 TCCAAAGGGATCGCATTG-ATCC
 23
                  |||||||||||||||||**|||*
Sbjct:       2002 AGGTTTCCCTAGCGTAACAGAGGT 1979
                     |||  |  ||   ||--------------------------------2
                     |||  |  ||   |---------------------------------1
                     |||  |  ||-------------------------------------2
                     |||  |  |--------------------------------------2
                     |||  |-----------------------------------------1
```

```
                         |||-------------------------------------------109
                         ||--------------------------------------------5
                         |---------------------------------------------1

>miR393b-5p
    Score: 1.5  Deg: 222:1064:222:1728 T_00015134

Query:           1 TCCAAAGGGATCGCATTGATCC
 23
                   |||||||||||||||||*|*|*
Sbjct:        1073 AGGTTTCCCTAGCGTAACAAAGT 1051
                    ||| | ||||||||||   | |------------------------------1
                    ||| | ||||||||||   |--------------------------------1
                    ||| | ||||||||||------------------------------------2
                    ||| | |||||||||-------------------------------------3
                    ||| | ||||||||--------------------------------------2
                    ||| | |||||||---------------------------------------3
                    ||| | ||||||----------------------------------------1
                    ||| | |||||-----------------------------------------1
                    ||| | ||||------------------------------------------1
                    ||| | |||-------------------------------------------222
                    ||| | ||--------------------------------------------1
                    ||| | |---------------------------------------------3
                    ||| |-----------------------------------------------1
                    |||-------------------------------------------------1
                    ||--------------------------------------------------1
                    |---------------------------------------------------2
>miR397-5p
    Score: 2.5  Deg: 16:256:36:4541T_00008962

Query:           1 TCATTGAGTGCAGCGTTGATG
 22
                   |*|||||||||||*|||||||*
Sbjct:         265 ACTAACTCACGTCTCAACTACT 244
                   |||||   ||||||||| ||||-------------------------------9
                   |||||   ||||||||| |||--------------------------------8
                   |||||   ||||||||| ||---------------------------------4
                   |||||   ||||||||| |----------------------------------4
                   |||||   |||||||||------------------------------------5
                   |||||   ||||||||-------------------------------------1
                   |||||   |||||||--------------------------------------2
                   |||||   ||||||---------------------------------------4
                   |||||   |||||----------------------------------------1
                   |||||   ||||-----------------------------------------2
                   |||||   |||------------------------------------------1
```

```
                  |||||   ||-------------------------------------------16
                  |||||   |--------------------------------------------5
                  |||||------------------------------------------------15
                  ||||-------------------------------------------------10
                  |||--------------------------------------------------6
                  ||---------------------------------------------------8
                  |----------------------------------------------------9

>miR397-5p
    Score: 1.2  Deg: 33:571:33:785 T_00059025

Query:          1 TCATTGAGTGCAGCGTTGATG
 22
                  ||||||||:|||||||||||:*
Sbjct:        580 AGTAACTCGCGTCGCAACTATT 559
                     | |||||||||||||||||-------------------------------2
                     | ||||||||||||||||--------------------------------1
                     | |||||||||||||||---------------------------------3
                     | ||||||||||||||----------------------------------3
                     | |||||||||||||-----------------------------------2
                     | ||||||||||||------------------------------------3
                     | |||||||||||-------------------------------------1
                     | ||||||||||--------------------------------------5
                     | |||||||||---------------------------------------1
                     | ||||||||----------------------------------------2
                     | |||||||-----------------------------------------3
                     | ||||||------------------------------------------2
                     | |||||-------------------------------------------33
                     | ||||--------------------------------------------3
                     | |||---------------------------------------------3
                     | ||----------------------------------------------4
                     | |-----------------------------------------------2
                     |-------------------------------------------------1

>miR399a-3p
    Score: 1.5  Deg: 5:369:6:352   T_00083396

Query:          1 TGCCAAAGGAGATTTGTCCTA
 22
                  ||||||||||||||||:||*|*
Sbjct:        378 ACGGTTTCCTCTAAACGGGTTG 357
                           |        |----------------------------------1
                           |-------------------------------------------5

>miR399a-3p
```

```
    Score: 1.5  Deg: 5:933:6:374   T_00083395

Query:          1 TGCCAAAGGAGATTTGTCCTA
 22
                  |||||||||||||||:||*|*
Sbjct:        942 ACGGTTTCCTCTAAACGGGTTG 921
                           |        |----------------------------------1
                           |-------------------------------------------5

>miR399a-3p
    Score: 1.5  Deg: 5:1027:433:7904   T_00083397

Query:          1 TGCCAAAGGAGATTTGTCCTA
 22
                  |||||||||||||||:||*|*
Sbjct:       1036 ACGGTTTCCTCTAAACGGGTTG 1015
                           |        |----------------------------------1
                           |-------------------------------------------5

>miR399a-3p
    Score: 1.5  Deg: 5:451:433:5148T_00083398

Query:          1 TGCCAAAGGAGATTTGTCCTA
 22
                  |||||||||||||||:||*|*
Sbjct:        460 ACGGTTTCCTCTAAACGGGTTG 439
                           |        |----------------------------------1
                           |-------------------------------------------5

>miR399b-3p
    Score: 1.5  Deg: 5:369:6:352   T_00083396

Query:          1 TGCCAAAGGAGATTTGTCCTA
 22
                  |||||||||||||||:||*|*
Sbjct:        378 ACGGTTTCCTCTAAACGGGTTG 357
                           |        |----------------------------------1
                           |-------------------------------------------5

>miR399b-3p
    Score: 1.5  Deg: 5:933:6:374   T_00083395

Query:          1 TGCCAAAGGAGATTTGTCCTA
 22
                  |||||||||||||||:||*|*
```

```
Sbjct:        942 ACGGTTTCCTCTAAACGGGTTG 921
                           |        |----------------------------------1
                           |------------------------------------------5

>miR399b-3p
    Score: 1.5  Deg: 5:1027:433:7904   T_00083397

Query:          1 TGCCAAAGGAGATTTGTCCTA
 22
                  ||||||||||||||||:||*|*
Sbjct:       1036 ACGGTTTCCTCTAAACGGGTTG 1015
                           |        |----------------------------------1
                           |------------------------------------------5

>miR399b-3p
    Score: 1.5  Deg: 5:451:433:5148T_00083398

Query:          1 TGCCAAAGGAGATTTGTCCTA
 22
                  ||||||||||||||||:||*|*
Sbjct:        460 ACGGTTTCCTCTAAACGGGTTG 439
                           |        |----------------------------------1
                           |------------------------------------------5

>miR399c-3P
    Score: 2.0  Deg: 63:403:63:419 T_00093857

Query:          1 TGCCAAAGGAGAGTTGCCCTG
 22
                  *|||||||||*|||||||||*
Sbjct:        412 TCGGTTTCCTCCCAACGGGACT 391
                  ||||||| || ||   |   ||-------------------------------2
                  ||||||| || ||   |   |--------------------------------2
                  ||||||| || ||   |------------------------------------2
                  ||||||| || ||----------------------------------------1
                  ||||||| || |-----------------------------------------1
                  ||||||| ||------------------------------------------63
                  ||||||| |-------------------------------------------5
                  |||||||---------------------------------------------1
                  ||||||----------------------------------------------3
                  |||||-----------------------------------------------2
                  ||||------------------------------------------------1
                  |||-------------------------------------------------3
                  ||--------------------------------------------------1
                  |---------------------------------------------------2
```

```
>miR399c-3P
    Score: 2.0  Deg: 63:403:63:492 T_00093858

Query:          1 TGCCAAAGGAGAGTTGCCCTG
 22
                  *||||||||||*|||||||||*
Sbjct:        412 TCGGTTTCCTCCCAACGGGACT 391
                  ||||||| || ||   |   ||-------------------------------2
                  ||||||| || ||   |   |--------------------------------2
                  ||||||| || ||   |------------------------------------2
                  ||||||| || ||----------------------------------------1
                  ||||||| || |-----------------------------------------1
                  ||||||| ||-------------------------------------------63
                  ||||||| |--------------------------------------------5
                  |||||||----------------------------------------------1
                  ||||||-----------------------------------------------3
                  |||||------------------------------------------------2
                  ||||-------------------------------------------------1
                  |||--------------------------------------------------3
                  ||---------------------------------------------------1
                  |----------------------------------------------------2

>miR399c-3P
    Score: 2.0  Deg: 63:367:63:262 T_00089452

Query:          1 TGCCAAAGGAGAGTTGCCCTG
 22
                  *||||||||||*|||||||||*
Sbjct:        376 TCGGTTTCCTCCCAACGGGACT 355
                  ||||||| || ||   |   ||-------------------------------2
                  ||||||| || ||   |   |--------------------------------2
                  ||||||| || ||   |------------------------------------2
                  ||||||| || ||----------------------------------------1
                  ||||||| || |-----------------------------------------1
                  ||||||| ||-------------------------------------------63
                  ||||||| |--------------------------------------------5
                  |||||||----------------------------------------------1
                  ||||||-----------------------------------------------3
                  |||||------------------------------------------------2
                  ||||-------------------------------------------------1
                  |||--------------------------------------------------3
                  ||---------------------------------------------------1
                  |----------------------------------------------------2
```

```
>miR399c-3P
    Score: 2.2  Deg: 5:369:6:352   T_00083396

Query:          1 TGCCAAAGGAGAGTTGCCCTG
 22
                  ||||||||||||*||||||*:*
Sbjct:        378 ACGGTTTCCTCTAAACGGGTTG 357
                           |        |----------------------------------1
                           |-------------------------------------------5

>miR399c-3P
    Score: 2.2  Deg: 5:933:6:374   T_00083395

Query:          1 TGCCAAAGGAGAGTTGCCCTG
 22
                  ||||||||||||*||||||*:*
Sbjct:        942 ACGGTTTCCTCTAAACGGGTTG 921
                           |        |----------------------------------1
                           |-------------------------------------------5
>miR399c-3P
    Score: 2.2  Deg: 5:1027:433:7904   T_00083397

Query:          1 TGCCAAAGGAGAGTTGCCCTG
 22
                  ||||||||||||*||||||*:*
Sbjct:       1036 ACGGTTTCCTCTAAACGGGTTG 1015
                           |        |----------------------------------1
                           |-------------------------------------------5
>miR399c-3P
    Score: 2.2  Deg: 5:451:433:5148T_00083398

Query:          1 TGCCAAAGGAGAGTTGCCCTG
 22
                  ||||||||||||*||||||*:*
Sbjct:        460 ACGGTTTCCTCTAAACGGGTTG 439
                           |        |----------------------------------1
                           |-------------------------------------------5
>miR399d-3p
    Score: 2.5  Deg: 63:403:63:419 T_00093857

Query:          1 TGCCAAAGGAGAGTTGCCCT-A
 22
                  *||||||||||*||||||||*|*
Sbjct:        412 TCGGTTTCCTCCCAACGGGACTA 390
                  ||||||| || ||    |    |||------------------------------1
```

```
                    ||||||| || ||    |    ||--------------------------------2
                    ||||||| || ||    |    |---------------------------------2
                    ||||||| || ||    |--------------------------------------2
                    ||||||| || ||-------------------------------------------1
                    ||||||| || |--------------------------------------------1
                    ||||||| ||----------------------------------------------63
                    ||||||| |-----------------------------------------------5
                    |||||||-------------------------------------------------1
                    ||||||--------------------------------------------------3
                    |||||---------------------------------------------------2
                    ||||----------------------------------------------------1
                    |||-----------------------------------------------------3
                    ||------------------------------------------------------1
                    |-------------------------------------------------------2

>miR399d-3p
    Score: 2.5  Deg: 63:403:63:492 T_00093858

Query:          1 TGCCAAAGGAGAGTTGCCCT-A
 22
                  *|||||||||*|||||||*|*
Sbjct:        412 TCGGTTTCCTCCCAACGGGACTA 390
                  ||||||| || ||    |    |||-------------------------------1
                  ||||||| || ||    |    ||--------------------------------2
                  ||||||| || ||    |    |---------------------------------2
                  ||||||| || ||    |--------------------------------------2
                  ||||||| || ||-------------------------------------------1
                  ||||||| || |--------------------------------------------1
                  ||||||| ||----------------------------------------------63
                  ||||||| |-----------------------------------------------5
                  |||||||-------------------------------------------------1
                  ||||||--------------------------------------------------3
                  |||||---------------------------------------------------2
                  ||||----------------------------------------------------1
                  |||-----------------------------------------------------3
                  ||------------------------------------------------------1
                  |-------------------------------------------------------2

>miR399d-3p
    Score: 2.5  Deg: 63:367:63:262 T_00089452

Query:          1 TGCCAAAGGAGAGTTGCCCT-A
 22
                  *|||||||||*|||||||*|*
Sbjct:        376 TCGGTTTCCTCCCAACGGGACTA 354
```

```
                    ||||||| || ||   |   |||------------------------------1
                    ||||||| || ||   |   ||-------------------------------2
                    ||||||| || ||   |   |--------------------------------2
                    ||||||| || ||   |------------------------------------2
                    ||||||| || ||----------------------------------------1
                    ||||||| || |-----------------------------------------1
                    ||||||| ||-------------------------------------------63
                    ||||||| |--------------------------------------------5
                    |||||||----------------------------------------------1
                    ||||||-----------------------------------------------3
                    |||||------------------------------------------------2
                    ||||-------------------------------------------------1
                    |||--------------------------------------------------3
                    ||---------------------------------------------------1
                    |----------------------------------------------------2

>miR399d-3p
    Score: 2.0  Deg: 5:369:6:352   T_00083396

Query:            1 TGCCAAAGGAGAGTTGCCCTA
 22
                    ||||||||||||*||||||*|*
Sbjct:          378 ACGGTTTCCTCTAAACGGGTTG 357
                             |        |----------------------------------1
                             |-------------------------------------------5

>miR399d-3p
    Score: 2.0  Deg: 5:933:6:374   T_00083395

Query:            1 TGCCAAAGGAGAGTTGCCCTA
 22
                    ||||||||||||*||||||*|*
Sbjct:          942 ACGGTTTCCTCTAAACGGGTTG 921
                             |        |----------------------------------1
                             |-------------------------------------------5

>miR399d-3p
    Score: 2.0  Deg: 5:1027:433:7904   T_00083397

Query:            1 TGCCAAAGGAGAGTTGCCCTA
 22
                    ||||||||||||*||||||*|*
Sbjct:         1036 ACGGTTTCCTCTAAACGGGTTG 1015
                             |        |----------------------------------1
                             |-------------------------------------------5
```

```
>miR399d-3p
    Score: 2.0  Deg: 5:451:433:5148T_00083398

Query:           1 TGCCAAAGGAGAGTTGCCCTA
 22
                   ||||||||||||*||||||*|*
Sbjct:         460 ACGGTTTCCTCTAAACGGGTTG 439
                            |        |----------------------------------1
                            |-------------------------------------------5

>miR403-3P
    Score: 0.8  Deg: 48:3295:48:229T_00080897

Query:           1 TTAGATTCACGCACAAACTCG
 22
                   ||||||||||||||||||:||*
Sbjct:        3304 AATCTAAGTGCGTGTTTGGGCT 3283
                            | |-----------------------------------------1
                            |-------------------------------------------48

>CsmiRNA-n5a-5p
    Score: 1.5  Deg: 18:1869:26:3282   T_00019479

Query:           1 CACATCAAAAAATGCTCGCACC
 23
                   ||||||||*||||||||||||*
Sbjct:        1878 GTGTAGTTTCTTACGAGCGTGGA 1856
                   |||||||||| | |     |   |-------------------------------1
                   |||||||||| | |     |-----------------------------------1
                   |||||||||| | |-----------------------------------------1
                   |||||||||| |-------------------------------------------1
                   ||||||||||---------------------------------------------18
                   |||||||||----------------------------------------------1
                   ||||||||-----------------------------------------------1
                   |||||||------------------------------------------------1
                   ||||||-------------------------------------------------2
                   |||||--------------------------------------------------2
                   ||||---------------------------------------------------2
                   |||----------------------------------------------------2
                   ||-----------------------------------------------------2
                   |------------------------------------------------------3

>CsmiRNA-n5b-5p
    Score: 1.5  Deg: 18:1869:26:3282   T_00019479
```

```
Query:          1 CACATCAAAAAATGCTCGCACC
 23
                  |||||||||*||||||||||||*
Sbjct:       1878 GTGTAGTTTCTTACGAGCGTGGA 1856
                  |||||||||| | |    |   |-------------------------------1
                  |||||||||| | |    |-----------------------------------1
                  |||||||||| | |----------------------------------------1
                  |||||||||| |------------------------------------------1
                  ||||||||||--------------------------------------------18
                  |||||||||---------------------------------------------1
                  ||||||||----------------------------------------------1
                  |||||||-----------------------------------------------1
                  ||||||------------------------------------------------2
                  |||||-------------------------------------------------2
                  ||||--------------------------------------------------2
                  |||---------------------------------------------------2
                  ||----------------------------------------------------2
                  |-----------------------------------------------------3

>CsmiRNA-n10a-3p
    Score: 2.0  Deg: 20:1660:21:1136   T_00069888

Query:          1 TAATGCAGTGCGAACTCTCTA
 22
                  |*|||||:|||||||||||||*
Sbjct:       1669 AATACGTTACGCTTGAGAGATA 1648
                    |   ||||  | || ||-----------------------------------1
                    |   ||||  | || |------------------------------------1
                    |   ||||  | ||--------------------------------------1
                    |   ||||  | |---------------------------------------1
                    |   ||||  |-----------------------------------------1
                    |   ||||--------------------------------------------20
                    |   |||---------------------------------------------1
                    |   ||----------------------------------------------2
                    |   |-----------------------------------------------1
                    |---------------------------------------------------2

>CsmiRNA-n10b-3p
    Score: 2.0  Deg: 20:1660:21:1136   T_00069888

Query:          1 TAATGCAGTGCGAACTCTCTA
 22
                  |*|||||:|||||||||||||*
Sbjct:       1669 AATACGTTACGCTTGAGAGATA 1648
                    |   ||||  | || ||-----------------------------------1
```

```
    |   ||||  | || |----------------------------------1
    |   ||||  | ||------------------------------------1
    |   ||||  | |-------------------------------------1
    |   ||||  |---------------------------------------1
    |   ||||------------------------------------------20
    |   |||-------------------------------------------1
    |   ||--------------------------------------------2
    |   |---------------------------------------------1
    |-------------------------------------------------2
```

>CsmiRNA-n10c-3p

```
    Score: 2.0  Deg: 20:1660:21:1136   T_00069888

Query:           1 TAATGCAGTGCGAACTCTCTA
 22
                   |*|||||:|||||||||||||*
Sbjct:        1669 AATACGTTACGCTTGAGAGATA 1648
                     |   ||||  | || ||---------------------------------1
                     |   ||||  | || |----------------------------------1
                     |   ||||  | ||------------------------------------1
                     |   ||||  | |-------------------------------------1
                     |   ||||  |---------------------------------------1
                     |   ||||------------------------------------------20
                     |   |||-------------------------------------------1
                     |   ||--------------------------------------------2
                     |   |---------------------------------------------1
                     |-------------------------------------------------2
```

>CsmiRNA-n10d-3p

```
    Score: 2.0  Deg: 20:1660:21:1136   T_00069888

Query:           1 TAATGCAGTGCGAACTCTCTA
 22
                   |*|||||:|||||||||||||*
Sbjct:        1669 AATACGTTACGCTTGAGAGATA 1648
                     |   ||||  | || ||---------------------------------1
                     |   ||||  | || |----------------------------------1
                     |   ||||  | ||------------------------------------1
                     |   ||||  | |-------------------------------------1
                     |   ||||  |---------------------------------------1
                     |   ||||------------------------------------------20
                     |   |||-------------------------------------------1
                     |   ||--------------------------------------------2
                     |   |---------------------------------------------1
                     |-------------------------------------------------2
```

```
>CsmiRNA-n27a.2
    Score: 2.5  Deg: 9:762:9:254   T_00013618

Query:          1 AATACAATACGACATAATACGGCG
 25
                  |||||||||||||**|||||||||*
Sbjct:        771 TTATGTTATGCTGGTTTATGCCGCA 747
                  || |||||||||||        ||--------------------------------1
                  || |||||||||||        |---------------------------------2
                  || |||||||||||------------------------------------------1
                  || ||||||||||-------------------------------------------6
                  || |||||||||--------------------------------------------1
                  || ||||||||---------------------------------------------2
                  || |||||||----------------------------------------------9
                  || ||||||-----------------------------------------------3
                  || |||||------------------------------------------------2
                  || ||||-------------------------------------------------1
                  || |||--------------------------------------------------7
                  || ||---------------------------------------------------4
                  || |----------------------------------------------------2
                  ||------------------------------------------------------2
                  |-------------------------------------------------------2

>CsmiRNA-n27a.2
    Score: 2.5  Deg: 9:1946:433:1737   T_00013615

Query:          1 AATACAATACGACATAATACGGCG
 25
                  |||||||||||||**|||||||||*
Sbjct:       1955 TTATGTTATGCTGGTTTATGCCGCA 1931
                  || |||||||||||        ||--------------------------------1
                  || |||||||||||        |---------------------------------2
                  || |||||||||||------------------------------------------1
                  || ||||||||||-------------------------------------------6
                  || |||||||||--------------------------------------------1
                  || ||||||||---------------------------------------------2
                  || |||||||----------------------------------------------9
                  || ||||||-----------------------------------------------3
                  || |||||------------------------------------------------2
                  || ||||-------------------------------------------------1
                  || |||--------------------------------------------------7
                  || ||---------------------------------------------------4
                  || |----------------------------------------------------2
                  ||------------------------------------------------------2
```

```
                  |-------------------------------------------------------2

>CsmiRNA-n27a.2
    Score: 2.5  Deg: 9:2739:433:1746   T_00013616

Query:          1 AATACAATACGACATAATACGGCG
 25
                  ||||||||||||**||||||||*
Sbjct:       2748 TTATGTTATGCTGGTTTATGCCGCA 2724
                  || |||||||||||        ||--------------------------------1
                  || |||||||||||        |---------------------------------2
                  || |||||||||||------------------------------------------1
                  || ||||||||||-------------------------------------------6
                  || |||||||||--------------------------------------------1
                  || ||||||||---------------------------------------------2
                  || |||||||----------------------------------------------9
                  || ||||||-----------------------------------------------3
                  || |||||------------------------------------------------2
                  || ||||-------------------------------------------------1
                  || |||--------------------------------------------------7
                  || ||---------------------------------------------------4
                  || |----------------------------------------------------2
                  ||------------------------------------------------------2
                  |-------------------------------------------------------2

>CsmiRNA-n27a.2
    Score: 2.5  Deg: 9:1977:433:1744   T_00013614

Query:          1 AATACAATACGACATAATACGGCG
 25
                  ||||||||||||**||||||||*
Sbjct:       1986 TTATGTTATGCTGGTTTATGCCGCA 1962
                  || |||||||||||        ||--------------------------------1
                  || |||||||||||        |---------------------------------2
                  || |||||||||||------------------------------------------1
                  || ||||||||||-------------------------------------------6
                  || |||||||||--------------------------------------------1
                  || ||||||||---------------------------------------------2
                  || |||||||----------------------------------------------9
                  || ||||||-----------------------------------------------3
                  || |||||------------------------------------------------2
                  || ||||-------------------------------------------------1
                  || |||--------------------------------------------------7
                  || ||---------------------------------------------------4
                  || |----------------------------------------------------2
```

```
                  ||------------------------------------------------------2
                  |-------------------------------------------------------2

>CsmiRNA-n27a.2
    Score: 2.5  Deg: 9:2108:433:1742   T_00013617

Query:          1 AATACAATACGACATAATACGGCG
 25
                  |||||||||||||**|||||||||*
Sbjct:       2117 TTATGTTATGCTGGTTTATGCCGCA 2093
                  || |||||||||||        ||--------------------------------1
                  || |||||||||||        |---------------------------------2
                  || |||||||||||------------------------------------------1
                  || ||||||||||-------------------------------------------6
                  || |||||||||--------------------------------------------1
                  || ||||||||---------------------------------------------2
                  || |||||||----------------------------------------------9
                  || ||||||-----------------------------------------------3
                  || |||||------------------------------------------------2
                  || ||||-------------------------------------------------1
                  || |||--------------------------------------------------7
                  || ||---------------------------------------------------4
                  || |----------------------------------------------------2
                  ||------------------------------------------------------2
                  |-------------------------------------------------------2

>CsmiRNA-n27g-3p
    Score: 2.0  Deg: 5:2286:631:2756   T_00015473

Query:          1 ATCTAATACGGCGTTCCAAACGAA
 25
                  |:||||||||:||||||||||||**
Sbjct:       2295 TGGATTATGCTGCAAGGTTTGCTCG 2271
                     |  | ||||||||| ||| |||-------------------------------1
                     |  | ||||||||| ||| ||--------------------------------2
                     |  | ||||||||| ||| |---------------------------------1
                     |  | ||||||||| |||-----------------------------------1
                     |  | ||||||||| ||------------------------------------1
                     |  | ||||||||| |-------------------------------------1
                     |  | |||||||||---------------------------------------2
                     |  | ||||||||----------------------------------------1
                     |  | |||||||-----------------------------------------1
                     |  | ||||||------------------------------------------1
                     |  | |||||-------------------------------------------2
                     |  | ||||--------------------------------------------4
```

```
                     |   | |||---------------------------------------------1
                     |   | ||----------------------------------------------5
                     |   | |-----------------------------------------------2
                     |   |-------------------------------------------------1
                     |---------------------------------------------------1

>CsmiRNA-n271
    Score: 1.5  Deg: 5:2286:631:2756   T_00015473

Query:          1 GACCTAATACGGTGTTCCAAACGA
 25
                  |||||||||||::|||||||||||*
Sbjct:       2296 CTGGATTATGCTGCAAGGTTTGCTC 2272
                      |  | ||||||||| ||| ||------------------------------2
                      |  | ||||||||| ||| |-------------------------------1
                      |  | ||||||||| |||---------------------------------1
                      |  | ||||||||| ||----------------------------------1
                      |  | ||||||||| |-----------------------------------1
                      |  | |||||||||-------------------------------------2
                      |  | ||||||||--------------------------------------1
                      |  | |||||||---------------------------------------1
                      |  | ||||||----------------------------------------1
                      |  | |||||-----------------------------------------2
                      |  | ||||------------------------------------------4
                      |  | |||-------------------------------------------1
                      |  | ||--------------------------------------------5
                      |  | |---------------------------------------------2
                      |  |-----------------------------------------------1
                      |--------------------------------------------------1

>CsmiRNA-n30n.2
    Score: 2.5  Deg: 6:183:6:60 T_00085947

Query:          1 ATTGTTGGATTTAATGACTTTTGT
 25
                  ||||||::||||||*|||||||||*
Sbjct:        192 TAACAATTTAAATTTCTGAAAACAG 168
                     ||    || | |------------------------------------2
                     ||    || |--------------------------------------1
                     ||    ||----------------------------------------6
                     ||    |-----------------------------------------2
                     ||----------------------------------------------1
                     |-----------------------------------------------1

>CsmiRNA-n30n.2
```

```
    Score: 2.5  Deg: 6:3060:6:267  T_00058209

Query:          1 ATTGTTGGATTTAATGACTTTTGT
 25
                  ||||||::||||||*|||||||||*
Sbjct:       3069 TAACAATTTAAATTTCTGAAAACAG 3045
                          || | |------------------------------------------2
                          || |--------------------------------------------1
                          ||----------------------------------------------6
                          |-----------------------------------------------2

>CsmiRNA-n30n.2
    Score: 2.5  Deg: 6:1259:6:104  T_00058207

Query:          1 ATTGTTGGATTTAATGACTTTTGT
 25
                  ||||||::||||||*|||||||||*
Sbjct:       1268 TAACAATTTAAATTTCTGAAAACAG 1244
                          || | |------------------------------------------2
                          || |--------------------------------------------1
                          ||----------------------------------------------6
                          |-----------------------------------------------2

>CsmiRNA-n30n.2
    Score: 2.5  Deg: 6:3031:6:305  T_00058208

Query:          1 ATTGTTGGATTTAATGACTTTTGT
 25
                  ||||||::||||||*|||||||||*
Sbjct:       3040 TAACAATTTAAATTTCTGAAAACAG 3016
                          || | |------------------------------------------2
                          || |--------------------------------------------1
                          ||----------------------------------------------6
                          |-----------------------------------------------2

>miR171a-3p
    Score: 1.0  Deg: 71:417:71:404 T_00060987

Query:          1 TGATTGAGCCGTGCCAATATC
 22
                  |||||||||||:|||||||||*
Sbjct:        429 ACTAACTCGGCGCGGTTATAGG 408
                           |  |------------------------------------------71
                           |---------------------------------------------4
>miR171a-3p
```

```
    Score: 1.0  Deg: 101:481:101:342   T_00010373

Query:          1 TGATTGAGCCGTGCCAATATC
 22
                  |||||||||||:|||||||||*
Sbjct:        493 ACTAACTCGGCGCGGTTATAGG 472
                           | ||----------------------------------------101
                           | |-----------------------------------------1
                           |-------------------------------------------18

>miR171b-3p
    Score: 1.0  Deg: 71:417:71:404 T_00060987

Query:          1 TGATTGAGCCGTGCCAATATC
 22
                  |||||||||||:|||||||||*
Sbjct:        429 ACTAACTCGGCGCGGTTATAGG 408
                           |  |----------------------------------------71
                           |-------------------------------------------4

>miR171b-3p
    Score: 1.0  Deg: 101:481:101:342   T_00010373

Query:          1 TGATTGAGCCGTGCCAATATC
 22
                  |||||||||||:|||||||||*
Sbjct:        493 ACTAACTCGGCGCGGTTATAGG 472
                           | ||----------------------------------------101
                           | |-----------------------------------------1
                           |-------------------------------------------18

>miR171c-3p
    Score: 1.0  Deg: 71:417:71:404 T_00060987

Query:          1 TGATTGAGCCGTGCCAATATC
 22
                  |||||||||||:|||||||||*
Sbjct:        429 ACTAACTCGGCGCGGTTATAGG 408
                           |  |----------------------------------------71
                           |-------------------------------------------4

>miR171c-3p
    Score: 1.0  Deg: 101:481:101:342   T_00010373

Query:          1 TGATTGAGCCGTGCCAATATC
```

```
 22
                 ||||||||||:|||||||||*
Sbjct:        493 ACTAACTCGGCGCGGTTATAGG 472
                          | ||----------------------------------------101
                          | |-----------------------------------------1
                          |-------------------------------------------18

>miR171d-3P
    Score: 1.0  Deg: 71:417:71:404 T_00060987

Query:          1 TGATTGAGCCGTGCCAATATC
 22
                 ||||||||||:|||||||||*
Sbjct:        429 ACTAACTCGGCGCGGTTATAGG 408
                          |  |----------------------------------------71
                          |-------------------------------------------4

>miR171d-3P
    Score: 1.0  Deg: 101:481:101:342   T_00010373

Query:          1 TGATTGAGCCGTGCCAATATC
 22
                 ||||||||||:|||||||||*
Sbjct:        493 ACTAACTCGGCGCGGTTATAGG 472
                          | ||----------------------------------------101
                          | |-----------------------------------------1
                          |-------------------------------------------18
>miR171e-3p
    Score: 1.0  Deg: 71:417:71:404 T_00060987

Query:          1 TGATTGAGCCGTGCCAATATC
 22
                 ||||||||||:|||||||||*
Sbjct:        429 ACTAACTCGGCGCGGTTATAGG 408
                          |  |----------------------------------------71
                          |-------------------------------------------4

>miR171e-3p
    Score: 1.0  Deg: 101:481:101:342   T_00010373

Query:          1 TGATTGAGCCGTGCCAATATC
 22
                 ||||||||||:|||||||||*
Sbjct:        493 ACTAACTCGGCGCGGTTATAGG 472
                          | ||----------------------------------------101
```

```
                       | |----------------------------------------1
                       |-----------------------------------------18
>miR171f-3p
    Score: 1.0  Deg: 71:417:71:404 T_00060987

Query:          1 TGATTGAGCCGTGCCAATATC
 22
                  |||||||||||:|||||||||*
Sbjct:        429 ACTAACTCGGCGCGGTTATAGG 408
                       |  |---------------------------------------71
                       |-----------------------------------------4

>miR171f-3p
    Score: 1.0  Deg: 101:481:101:342   T_00010373

Query:          1 TGATTGAGCCGTGCCAATATC
 22
                  |||||||||||:|||||||||*
Sbjct:        493 ACTAACTCGGCGCGGTTATAGG 472
                       | ||---------------------------------------101
                       | |----------------------------------------1
                       |-----------------------------------------18

>miR530-5P
    Score: 0.8  Deg: 24:1277:26:827T_00079842

Query:          1 TCTGCATTTGCACCTGCACCT
 22
                  :|||||||||||||||||||*
Sbjct:       1288 GGACGTAAACGTGGACGTGGAC 1267
                      |      |     |----------------------------------1
                      |      |----------------------------------------24
                      |-----------------------------------------------1

>miR530-5P
    Score: 0.8  Deg: 24:1277:26:815T_00079843

Query:          1 TCTGCATTTGCACCTGCACCT
 22
                  :|||||||||||||||||||*
Sbjct:       1288 GGACGTAAACGTGGACGTGGAC 1267
                      |      |     |----------------------------------1
                      |      |----------------------------------------24
                      |-----------------------------------------------1
```

```
>miR5658
    Score: 2.2  Deg: 6:1386:630:2486   T_00032450

Query:          1 ATGATGATCATCATGATGAT-A
 22
                  ||||||||*||||||||||:*|*
Sbjct:       1398 TACTACTACTAGTACTACTGCTC 1376
                         || | |-----------------------------------------6
                         || |-------------------------------------------2
                         ||---------------------------------------------1
                         |----------------------------------------------2
>miR5658
    Score: 2.2  Deg: 6:1386:630:2485   T_00032452

Query:          1 ATGATGATCATCATGATGAT-A
 22
                  ||||||||*||||||||||:*|*
Sbjct:       1398 TACTACTACTAGTACTACTGCTC 1376
                         || | |-----------------------------------------6
                         || |-------------------------------------------2
                         ||---------------------------------------------1
                         |----------------------------------------------2
>CsmiRNA-n27g-3p
    Score: 2.5  Deg: 6:759:9:254   T_00013618

Query:          1 ATCTAATACGGCGTTCCAAACGAA
 25
                  |:|*|||||||||||||||||||**
Sbjct:        760 TGGTTTATGCCGCAAGGTTTGCTCG 736
                  |||        ||    |      |-------------------------------1
                  |||        ||    |--------------------------------------1
                  |||        ||-------------------------------------------1
                  |||        |--------------------------------------------2
                  |||-----------------------------------------------------1
                  ||------------------------------------------------------6
                  |-------------------------------------------------------1
>CsmiRNA-n27g-3p
    Score: 2.5  Deg: 6:1943:433:1737   T_00013615

Query:          1 ATCTAATACGGCGTTCCAAACGAA
 25
                  |:|*|||||||||||||||||||**
Sbjct:       1944 TGGTTTATGCCGCAAGGTTTGCTCG 1920
                  |||        ||    |      |-------------------------------1
                  |||        ||    |--------------------------------------1
```

```
                 |||        ||-------------------------------------------1
                 |||        |--------------------------------------------2
                 |||---------------------------------------------------1
                 ||----------------------------------------------------6
                 |-----------------------------------------------------1

>CsmiRNA-n27g-3p
    Score: 2.5  Deg: 6:2736:433:1746   T_00013616

Query:          1 ATCTAATACGGCGTTCCAAACGAA
 25
                 |:|*||||||||||||||||||**
Sbjct:       2737 TGGTTTATGCCGCAAGGTTTGCTCG 2713
                 |||        ||     |      |-------------------------------1
                 |||        ||     |--------------------------------------1
                 |||        ||-------------------------------------------1
                 |||        |--------------------------------------------2
                 |||---------------------------------------------------1
                 ||----------------------------------------------------6
                 |-----------------------------------------------------1

>CsmiRNA-n27g-3p
    Score: 2.5  Deg: 6:1974:433:1744   T_00013614

Query:          1 ATCTAATACGGCGTTCCAAACGAA
 25
                 |:|*||||||||||||||||||**
Sbjct:       1975 TGGTTTATGCCGCAAGGTTTGCTCG 1951
                 |||        ||     |      |-------------------------------1
                 |||        ||     |--------------------------------------1
                 |||        ||-------------------------------------------1
                 |||        |--------------------------------------------2
                 |||---------------------------------------------------1
                 ||----------------------------------------------------6
                 |-----------------------------------------------------1

>CsmiRNA-n27g-3p
    Score: 2.5  Deg: 6:2105:433:1742   T_00013617

Query:          1 ATCTAATACGGCGTTCCAAACGAA
 25
                 |:|*||||||||||||||||||**
Sbjct:       2106 TGGTTTATGCCGCAAGGTTTGCTCG 2082
                 |||        ||     |      |-------------------------------1
                 |||        ||     |--------------------------------------1
```

```
                  |||        ||------------------------------------------1
                  |||        |-------------------------------------------2
                  |||----------------------------------------------------1
                  ||-----------------------------------------------------6
                  |------------------------------------------------------1
>CsmiRNA-n271
    Score: 2.0  Deg: 6:759:9:254   T_00013618

Query:          1 GACCTAATACGGTGTTCCAAACGA
 25
                  ||||*|||||||:|||||||||||*
Sbjct:        761 CTGGTTTATGCCGCAAGGTTTGCTC 737
                  ||||        ||     |------------------------------------1
                  ||||        ||-----------------------------------------1
                  ||||        |------------------------------------------2
                  ||||---------------------------------------------------1
                  |||----------------------------------------------------6
                  ||-----------------------------------------------------1
                  |------------------------------------------------------2

>CsmiRNA-n271
    Score: 2.0  Deg: 6:1943:433:1737   T_00013615

Query:          1 GACCTAATACGGTGTTCCAAACGA
 25
                  ||||*|||||||:|||||||||||*
Sbjct:       1945 CTGGTTTATGCCGCAAGGTTTGCTC 1921
                  ||||        ||     |------------------------------------1
                  ||||        ||-----------------------------------------1
                  ||||        |------------------------------------------2
                  ||||---------------------------------------------------1
                  |||----------------------------------------------------6
                  ||-----------------------------------------------------1
                  |------------------------------------------------------2

>CsmiRNA-n271
    Score: 2.0  Deg: 6:2736:433:1746   T_00013616

Query:          1 GACCTAATACGGTGTTCCAAACGA
 25
                  ||||*|||||||:|||||||||||*
Sbjct:       2738 CTGGTTTATGCCGCAAGGTTTGCTC 2714
                  ||||        ||     |------------------------------------1
                  ||||        ||-----------------------------------------1
                  ||||        |------------------------------------------2
```

```
                  ||||----------------------------------------------------1
                  |||-----------------------------------------------------6
                  ||------------------------------------------------------1
                  |-------------------------------------------------------2

>CsmiRNA-n271
    Score: 2.0  Deg: 6:1974:433:1744   T_00013614

Query:          1 GACCTAATACGGTGTTCCAAACGA
 25
                  ||||*|||||||:|||||||||||*
Sbjct:       1976 CTGGTTTATGCCGCAAGGTTTGCTC 1952
                  ||||        ||    |-------------------------------------1
                  ||||        ||------------------------------------------1
                  ||||        |-------------------------------------------2
                  ||||----------------------------------------------------1
                  |||-----------------------------------------------------6
                  ||------------------------------------------------------1
                  |-------------------------------------------------------2

>CsmiRNA-n271
    Score: 2.0  Deg: 6:2105:433:1742   T_00013617

Query:          1 GACCTAATACGGTGTTCCAAACGA
 25
                  ||||*|||||||:|||||||||||*
Sbjct:       2107 CTGGTTTATGCCGCAAGGTTTGCTC 2083
                  ||||        ||    |-------------------------------------1
                  ||||        ||------------------------------------------1
                  ||||        |-------------------------------------------2
                  ||||----------------------------------------------------1
                  |||-----------------------------------------------------6
                  ||------------------------------------------------------1
                  |-------------------------------------------------------2
>CsmiRNA-n30a
    Score: 2.0  Deg: 6:183:6:60T_00085947

Query:          1 AAGATTGTTGGATTTAAAGACTTT
 25
                  *||||||||::|||||||||||||*
Sbjct:        195 CTCTAACAATTTAAATTTCTGAAAA 171
                        ||   || | |---------------------------------------2
                        ||   || |-----------------------------------------1
                        ||   ||-------------------------------------------6
                        ||   |--------------------------------------------2
```

```
                       ||-------------------------------------------------1
                       |--------------------------------------------------1
>CsmiRNA-n30a
    Score: 2.0  Deg: 6:3060:6:267  T_00058209

Query:         1 AAGATTGTTGGATTTAAAGACTTT
 25
                 *||||||||::|||||||||||||*
Sbjct:      3072 CTCTAACAATTTAAATTTCTGAAAA 3048
                            || | |----------------------------------------2
                            || |------------------------------------------1
                            ||--------------------------------------------6
                            |---------------------------------------------2

>CsmiRNA-n30a
    Score: 2.0  Deg: 6:1259:6:104  T_00058207

Query:         1 AAGATTGTTGGATTTAAAGACTTT
 25
                 *||||||||::|||||||||||||*
Sbjct:      1271 CTCTAACAATTTAAATTTCTGAAAA 1247
                            || | |----------------------------------------2
                            || |------------------------------------------1
                            ||--------------------------------------------6
                            |---------------------------------------------2

>CsmiRNA-n30a
    Score: 2.0  Deg: 6:3031:6:305  T_00058208

Query:         1 AAGATTGTTGGATTTAAAGACTTT
 25
                 *||||||||::|||||||||||||*
Sbjct:      3043 CTCTAACAATTTAAATTTCTGAAAA 3019
                            || | |----------------------------------------2
                            || |------------------------------------------1
                            ||--------------------------------------------6
                            |---------------------------------------------2
>CsmiRNA-n30c.1-3p
    Score: 2.5  Deg: 5:725:631:5310T_00078015

Query:         1 AGTTCAGGGGGTAGGATTTGGTAC
 25
                 |||||||||||:|*|||||::|||*
Sbjct:       736 TCAAGTCCCCCGTACTAAATTATGT 712
                           ||     |---------------------------------------1
```

```
                    ||---------------------------------------------5
                    |----------------------------------------------2

>CsmiRNA-n30c.1-3p
    Score: 2.5  Deg: 5:1381:631:5317   T_00078013

Query:          1 AGTTCAGGGGGTAGGATTTGGTAC
 25
                  |||||||||||:|*|||||::|||*
Sbjct:       1392 TCAAGTCCCCCGTACTAAATTATGT 1368
                            ||      |---------------------------------------1
                            ||---------------------------------------------5
                            |----------------------------------------------2

>CsmiRNA-n30c.1-3p
    Score: 2.5  Deg: 5:1165:631:5317   T_00078014

Query:          1 AGTTCAGGGGGTAGGATTTGGTAC
 25
                  |||||||||||:|*|||||::|||*
Sbjct:       1176 TCAAGTCCCCCGTACTAAATTATGT 1152
                            ||      |---------------------------------------1
                            ||---------------------------------------------5
                            |----------------------------------------------2
>CsmiRNA-n30d
    Score: 2.0  Deg: 5:727:5:201   T_00053092

Query:          1 AACAATCTTAATAGTTCAGGAAGA
 25
                  ||||||||:||||||||||||**|*
Sbjct:        746 TTGTTAGAGTTATCAAGTCCTCATA 722
                                    |||------------------------------------1
                                    ||-------------------------------------5
                                    |--------------------------------------2
>CsmiRNA-n30e.2-5p
    Score: 1.5  Deg: 6:183:6:60T_00085947

Query:          1 TTGAGATGGTTAAATTTAAAGACT
 25
                  |||||||*|||||||||||||||*
Sbjct:        197 AACTCTAACAATTTAAATTTCTGAA 173
                   |      ||    || | |--------------------------------------2
                   |      ||    || |---------------------------------------1
                   |      ||    ||-----------------------------------------6
                   |      ||    |------------------------------------------2
```

```
                   |      ||-----------------------------------------------1
                   |      |------------------------------------------------1
                   |-------------------------------------------------------1
>CsmiRNA-n30e.2-5p
    Score: 2.5  Deg: 6:2168:6:31   T_00095104

Query:          1 TTGAGATGGTTAAATTTAAAGACT
 25
                  |||||||*|||||||||*||||*|*
Sbjct:       2182 AACTCTAACAATTTAAA-TTCTTAA 2159
                          |    || |  |-------------------------------------2
                          |    || |----------------------------------------1
                          |    ||------------------------------------------6
                          |    |-------------------------------------------2
                          |------------------------------------------------1

>CsmiRNA-n30e.2-5p
    Score: 1.5  Deg: 6:3060:6:267  T_00058209

Query:          1 TTGAGATGGTTAAATTTAAAGACT
 25
                  |||||||*||||||||||||||||*
Sbjct:       3074 AACTCTAACAATTTAAATTTCTGAA 3050
                  |            || | |--------------------------------------2
                  |            || |----------------------------------------1
                  |            ||------------------------------------------6
                  |            |-------------------------------------------2
                  |--------------------------------------------------------1

>CsmiRNA-n30e.2-5p
    Score: 1.5  Deg: 6:1259:6:104  T_00058207

Query:          1 TTGAGATGGTTAAATTTAAAGACT
 25
                  |||||||*||||||||||||||||*
Sbjct:       1273 AACTCTAACAATTTAAATTTCTGAA 1249
                  |            || | |--------------------------------------2
                  |            || |----------------------------------------1
                  |            ||------------------------------------------6
                  |            |-------------------------------------------2
                  |--------------------------------------------------------1

>CsmiRNA-n30e.2-5p
    Score: 1.5  Deg: 6:3031:6:305  T_00058208
```

```
Query:          1 TTGAGATGGTTAAATTTAAAGACT
 25
                  |||||||*|||||||||||||||*
Sbjct:       3045 AACTCTAACAATTTAAATTTCTGAA 3021
                  |            || | |--------------------------------------2
                  |            || |----------------------------------------1
                  |            ||------------------------------------------6
                  |            |-------------------------------------------2
                  |--------------------------------------------------------1

>CsmiRNA-n30e.2-5p
    Score: 2.5  Deg: 6:1025:6:56   T_00060631

Query:          1 TTGAGATGGTTAAATTTAAAGACT
 25
                  |||||||*|||||||||*||||*|*
Sbjct:       1039 AACTCTAACAATTTAAA-TTCTTAA 1016
                           |   || |   |------------------------------------2
                           |   || |---------------------------------------1
                           |   ||------------------------------------------6
                           |   |-------------------------------------------2
                           |-----------------------------------------------1

>CsmiRNA-n30g.1
    Score: 1.5  Deg: 6:1741:7:490  T_00037223

Query:          1 ATTGAGATTGTTACATTTAA
 21
                  ||||||||||||*||||||*
Sbjct:       1756 TAACTCTAACAATTTAAATTC 1736
                    |       | |  || | |-------------------------------2
                    |       | |  || |---------------------------------1
                    |       | |  ||-----------------------------------6
                    |       | |  |------------------------------------1
                    |       | |---------------------------------------1
                    |       |-----------------------------------------1
                    |-------------------------------------------------1
>CsmiRNA-n30g.1
    Score: 1.5  Deg: 6:995:6:138   T_00078037

Query:          1 ATTGAGATTGTTACATTTAA
 21
                  ||||||||||||*||||||*
Sbjct:       1010 TAACTCTAACAATTTAAATTC 990
                               | || | |-------------------------------2
```

```
                               | || |----------------------------------1
                               | ||------------------------------------6
                               | |-------------------------------------1
                               |---------------------------------------1
>CsmiRNA-n30g.1
    Score: 1.5  Deg: 6:12:10:680   T_00050514

Query:          1 ATTGAGATTGTTACATTTAA
 21
                  |||||||||||||*||||||*
Sbjct:         27 TAACTCTAACAATTTAAATTC 7
                               | || | |--------------------------------2
                               | || |----------------------------------1
                               | ||------------------------------------6
                               | |-------------------------------------1
                               |---------------------------------------1

>CsmiRNA-n30g.1
    Score: 1.5  Deg: 6:12:10:681   T_00050513

Query:          1 ATTGAGATTGTTACATTTAA
 21
                  |||||||||||||*||||||*
Sbjct:         27 TAACTCTAACAATTTAAATTC 7
                               | || | |--------------------------------2
                               | || |----------------------------------1
                               | ||------------------------------------6
                               | |-------------------------------------1
                               |---------------------------------------1

>CsmiRNA-n30g.1
    Score: 1.5  Deg: 6:12:10:678   T_00050515

Query:          1 ATTGAGATTGTTACATTTAA
 21
                  |||||||||||||*||||||*
Sbjct:         27 TAACTCTAACAATTTAAATTC 7
                               | || | |--------------------------------2
                               | || |----------------------------------1
                               | ||------------------------------------6
                               | |-------------------------------------1
                               |---------------------------------------1

>CsmiRNA-n30g.1
    Score: 1.5  Deg: 6:183:6:60T_00085947
```

```
Query:          1 ATTGAGATTGTTACATTTAA
 21
                  |||||||||||||*||||||*
Sbjct:        198 TAACTCTAACAATTTAAATTT 178
                    |      ||   || | |--------------------------------2
                    |      ||   || |----------------------------------1
                    |      ||   ||------------------------------------6
                    |      ||   |-------------------------------------2
                    |      ||-----------------------------------------1
                    |      |------------------------------------------1
                    |-------------------------------------------------1
>CsmiRNA-n30g.1
    Score: 1.5  Deg: 6:2168:6:31   T_00095104

Query:          1 ATTGAGATTGTTACATTTAA
 21
                  |||||||||||||*||||||*
Sbjct:       2183 TAACTCTAACAATTTAAATTC 2163
                           |    || | |--------------------------------2
                           |    || |----------------------------------1
                           |    ||------------------------------------6
                           |    |-------------------------------------2
                           |------------------------------------------1

>CsmiRNA-n30g.1
    Score: 1.5  Deg: 6:3060:6:267  T_00058209

Query:          1 ATTGAGATTGTTACATTTAA
 21
                  |||||||||||||*||||||*
Sbjct:       3075 TAACTCTAACAATTTAAATTT 3055
                   |            || | |--------------------------------2
                   |            || |----------------------------------1
                   |            ||------------------------------------6
                   |            |-------------------------------------2
                   |--------------------------------------------------1

>CsmiRNA-n30g.1
    Score: 1.5  Deg: 6:1259:6:104  T_00058207

Query:          1 ATTGAGATTGTTACATTTAA
 21
                  |||||||||||||*||||||*
Sbjct:       1274 TAACTCTAACAATTTAAATTT 1254
```

```
                   |            || | |--------------------------------2
                   |            || |----------------------------------1
                   |            ||------------------------------------6
                   |            |-------------------------------------2
                   |--------------------------------------------------1

>CsmiRNA-n30g.1
    Score: 1.5  Deg: 6:3031:6:305  T_00058208

Query:          1 ATTGAGATTGTTACATTTAA
 21
                  |||||||||||||*||||||*
Sbjct:       3046 TAACTCTAACAATTTAAATTT 3026
                   |            || | |--------------------------------2
                   |            || |----------------------------------1
                   |            ||------------------------------------6
                   |            |-------------------------------------2
                   |--------------------------------------------------1
>CsmiRNA-n30g.1
    Score: 1.5  Deg: 6:1025:6:56   T_00060631

Query:          1 ATTGAGATTGTTACATTTAA
 21
                  |||||||||||||*||||||*
Sbjct:       1040 TAACTCTAACAATTTAAATTC 1020
                           |    || | |--------------------------------2
                           |    || |----------------------------------1
                           |    ||------------------------------------6
                           |    |-------------------------------------2
                           |------------------------------------------1
>CsmiRNA-n30h.1
    Score: 2.0  Deg: 6:1741:7:490  T_00037223

Query:          1 TGAACTATCGAGATTGTTAGATTT
 25
                  :|||||||*||||||||||:||||*
Sbjct:       1762 GCTTGATAACTCTAACAATTTAAAT 1738
                  |       |      | |  || |---------------------------------1
                  |       |      | |  ||-----------------------------------6
                  |       |      | |  |------------------------------------1
                  |       |      | |---------------------------------------1
                  |       |      |-----------------------------------------1
                  |       |------------------------------------------------1
                  |--------------------------------------------------------1
>CsmiRNA-n30h.1
```

```
    Score: 2.0  Deg: 6:995:6:138   T_00078037

Query:           1 TGAACTATCGAGATTGTTAGATTT
 25
                   :|||||||*||||||||||:||||*
Sbjct:        1016 GCTTGATAACTCTAACAATTTAAAT 992
                                    | || |--------------------------------1
                                    | ||----------------------------------6
                                    | |-----------------------------------1
                                    |-------------------------------------1
>CsmiRNA-n30h.1
    Score: 2.0  Deg: 6:12:10:680   T_00050514

Query:           1 TGAACTATCGAGATTGTTAGATTT
 25
                   :|||||||*||||||||||:||||*
Sbjct:          33 GCTTGATAACTCTAACAATTTAAAT 9
                                    | || |--------------------------------1
                                    | ||----------------------------------6
                                    | |-----------------------------------1
                                    |-------------------------------------1

>CsmiRNA-n30h.1
    Score: 2.0  Deg: 6:12:10:681   T_00050513

Query:           1 TGAACTATCGAGATTGTTAGATTT
 25
                   :|||||||*||||||||||:||||*
Sbjct:          33 GCTTGATAACTCTAACAATTTAAAT 9
                                    | || |--------------------------------1
                                    | ||----------------------------------6
                                    | |-----------------------------------1
                                    |-------------------------------------1

>CsmiRNA-n30h.1
    Score: 2.0  Deg: 6:12:10:678   T_00050515

Query:           1 TGAACTATCGAGATTGTTAGATTT
 25
                   :|||||||*||||||||||:||||*
Sbjct:          33 GCTTGATAACTCTAACAATTTAAAT 9
                                    | || |--------------------------------1
                                    | ||----------------------------------6
                                    | |-----------------------------------1
                                    |-------------------------------------1
```

```
>CsmiRNA-n30h.1
    Score: 1.8  Deg: 6:183:6:60 T_00085947

Query:          1 TGAACTATCGAGATTGTTAGATTT
 25
                  ||||||||*||||||||||:||||*
Sbjct:        204 ACTTGATAACTCTAACAATTTAAAT 180
                          |      ||   || |--------------------------------1
                          |      ||   ||----------------------------------6
                          |      ||   |-----------------------------------2
                          |      ||-------------------------------------1
                          |      |--------------------------------------1
                          |----------------------------------------------1
>CsmiRNA-n30h.1
    Score: 1.8  Deg: 6:2168:6:31   T_00095104

Query:          1 TGAACTATCGAGATTGTTAGATTT
 25
                  ||||||||*||||||||||:||||*
Sbjct:       2189 ACTTGATAACTCTAACAATTTAAAT 2165
                                 |    || |--------------------------------1
                                 |    ||----------------------------------6
                                 |    |-----------------------------------2
                                 |--------------------------------------1

>CsmiRNA-n30h.1
    Score: 1.8  Deg: 6:3060:6:267  T_00058209

Query:          1 TGAACTATCGAGATTGTTAGATTT
 25
                  ||||||||*||||||||||:||||*
Sbjct:       3081 ACTTGATAACTCTAACAATTTAAAT 3057
                         |            || |--------------------------------1
                         |            ||----------------------------------6
                         |            |-----------------------------------2
                         |-----------------------------------------------1

>CsmiRNA-n30h.1
    Score: 1.8  Deg: 6:1259:6:104  T_00058207

Query:          1 TGAACTATCGAGATTGTTAGATTT
 25
                  ||||||||*||||||||||:||||*
Sbjct:       1280 ACTTGATAACTCTAACAATTTAAAT 1256
```

```
                       |            || |------------------------------1
                       |            ||--------------------------------6
                       |            |---------------------------------2
                       |----------------------------------------------1

>CsmiRNA-n30h.1
    Score: 1.8  Deg: 6:3031:6:305  T_00058208

Query:          1 TGAACTATCGAGATTGTTAGATTT
 25
                  ||||||||*||||||||||:||||*
Sbjct:       3052 ACTTGATAACTCTAACAATTTAAAT 3028
                       |            || |------------------------------1
                       |            ||--------------------------------6
                       |            |---------------------------------2
                       |----------------------------------------------1
>CsmiRNA-n30h.1
    Score: 1.8  Deg: 6:1025:6:56   T_00060631

Query:          1 TGAACTATCGAGATTGTTAGATTT
 25
                  ||||||||*||||||||||:||||*
Sbjct:       1046 ACTTGATAACTCTAACAATTTAAAT 1022
                               |    || |------------------------------1
                               |    ||--------------------------------6
                               |    |---------------------------------2
                               |--------------------------------------1
>CsmiRNA-n30m
    Score: 2.5  Deg: 6:183:6:60T_00085947

Query:          1 ATTGAGATTATTAGATTTAAGGAT
 25
                  |||||||||*|||:||||||:||:*
Sbjct:        198 TAACTCTAACAATTTAAATTTCTGA 174
                    |      ||   || | |----------------------------------2
                    |      ||   || |------------------------------------1
                    |      ||   ||--------------------------------------6
                    |      ||   |---------------------------------------2
                    |      ||-------------------------------------------1
                    |      |--------------------------------------------1
                    |---------------------------------------------------1
>CsmiRNA-n30m
    Score: 2.2  Deg: 6:2168:6:31   T_00095104

Query:          1 ATTGAGATTATTAGATTTAAGGAT
```

```
 25
                  |||||||||*|||:|||||||:||*
Sbjct:       2183 TAACTCTAACAATTTAAATTCTTAA 2159
                           |    || | |------------------------------------2
                           |    || |--------------------------------------1
                           |    ||----------------------------------------6
                           |    |-----------------------------------------2
                           |----------------------------------------------1

>CsmiRNA-n30m
    Score: 2.5  Deg: 6:3060:6:267  T_00058209

Query:          1 ATTGAGATTATTAGATTTAAGGAT
 25
                  |||||||||*|||:||||||:||:*
Sbjct:       3075 TAACTCTAACAATTTAAATTTCTGA 3051
                   |            || | |------------------------------------2
                   |            || |--------------------------------------1
                   |            ||----------------------------------------6
                   |            |-----------------------------------------2
                   |------------------------------------------------------1

>CsmiRNA-n30m
    Score: 2.5  Deg: 6:1259:6:104  T_00058207

Query:          1 ATTGAGATTATTAGATTTAAGGAT
 25
                  |||||||||*|||:||||||:||:*
Sbjct:       1274 TAACTCTAACAATTTAAATTTCTGA 1250
                   |            || | |------------------------------------2
                   |            || |--------------------------------------1
                   |            ||----------------------------------------6
                   |            |-----------------------------------------2
                   |------------------------------------------------------1

>CsmiRNA-n30m
    Score: 2.5  Deg: 6:3031:6:305  T_00058208

Query:          1 ATTGAGATTATTAGATTTAAGGAT
 25
                  |||||||||*|||:||||||:||:*
Sbjct:       3046 TAACTCTAACAATTTAAATTTCTGA 3022
                   |            || | |------------------------------------2
                   |            || |--------------------------------------1
                   |            ||----------------------------------------6
```

```
                    |             |------------------------------------------2
                    |----------------------------------------------------------1
>CsmiRNA-n30m
    Score: 2.2  Deg: 6:1025:6:56   T_00060631

Query:          1 ATTGAGATTATTAGATTTAAGGAT
 25
                  |||||||||*|||:|||||||:||*
Sbjct:       1040 TAACTCTAACAATTTAAATTCTTAA 1016
                           |    || | |-------------------------------------2
                           |    || |---------------------------------------1
                           |    ||-----------------------------------------6
                           |    |------------------------------------------2
                           |-----------------------------------------------1
>CsmiRNA-n30n.1
    Score: 2.0  Deg: 5:3074:5:57   T_00058193

Query:          1 GGGTATAATTTGGTACATGTCAAA
 25
                  |||:|:|||||:||||||||||||*
Sbjct:       3077 CCCGTGTTAAATCATGTACAGTTTT 3053
                    || |---------------------------------------------------1
                    ||-----------------------------------------------------5
                    |------------------------------------------------------2

>CsmiRNA-n30n.1
    Score: 2.0  Deg: 5:2998:5:57   T_00058194

Query:          1 GGGTATAATTTGGTACATGTCAAA
 25
                  |||:|:|||||:||||||||||||*
Sbjct:       3001 CCCGTGTTAAATCATGTACAGTTTT 2977
                    || |---------------------------------------------------1
                    ||-----------------------------------------------------5
                    |------------------------------------------------------2

>CsmiRNA-n30n.1
    Score: 2.0  Deg: 5:3148:5:62   T_00058192

Query:          1 GGGTATAATTTGGTACATGTCAAA
 25
                  |||:|:|||||:||||||||||||*
Sbjct:       3151 CCCGTGTTAAATCATGTACAGTTTT 3127
                    || |---------------------------------------------------1
                    ||-----------------------------------------------------5
```

```
                    |---------------------------------------------------2

>CsmiRNA-n30n.1
    Score: 2.0  Deg: 5:3023:5:57   T_00058196

Query:          1 GGGTATAATTTGGTACATGTCAAA
 25
                  |||:|:|||||:||||||||||||*
Sbjct:       3026 CCCGTGTTAAATCATGTACAGTTTT 3002
                    || |---------------------------------------------------1
                    ||-----------------------------------------------------5
                    |---------------------------------------------------2

>CsmiRNA-n30n.1
    Score: 2.0  Deg: 5:2944:5:57   T_00058195

Query:          1 GGGTATAATTTGGTACATGTCAAA
 25
                  |||:|:|||||:||||||||||||*
Sbjct:       2947 CCCGTGTTAAATCATGTACAGTTTT 2923
                    || |---------------------------------------------------1
                    ||-----------------------------------------------------5
                    |---------------------------------------------------2
>CsmiRNA-n30n.1
    Score: 2.0  Deg: 5:1086:26:635 T_00050741

Query:          1 GGGTATAATTTGGTACATGTCAAA
 25
                  |||:|:|||||:||||||||||||*
Sbjct:       1089 CCCGTGTTAAATCATGTACAGTTTC 1065
                    || |---------------------------------------------------1
                    ||-----------------------------------------------------5
                    |---------------------------------------------------2
>CsmiRNA-n30o-5p
    Score: 1.0  Deg: 5:140:5:56T_00091038

Query:          1 CTTTGACATGTACTAAATTATGGT
 25
                  |||||||||||||||||||||*|*
Sbjct:        156 GAAACTGTACATGATTTAATACAAA 132
                               | |   |-----------------------------------1
                               | |--------------------------------------5
                               |-----------------------------------------1

>CsmiRNA-n30o-5p
```

```
    Score: 1.0  Deg: 5:140:5:56 T_00091039

Query:           1 CTTTGACATGTACTAAATTATGGT
 25
                   |||||||||||||||||||||*|*
Sbjct:         156 GAAACTGTACATGATTTAATACAAA 132
                                 | |    |-----------------------------------1
                                 | |---------------------------------------5
                                 |-----------------------------------------1

>CsmiRNA-n30o-5p
    Score: 1.0  Deg: 5:359:5:55 T_00091040

Query:           1 CTTTGACATGTACTAAATTATGGT
 25
                   |||||||||||||||||||||*|*
Sbjct:         375 GAAACTGTACATGATTTAATACAAA 351
                                 | |    |-----------------------------------1
                                 | |---------------------------------------5
                                 |-----------------------------------------1

>CsmiRNA-n30o-5p
    Score: 1.0  Deg: 5:140:5:56 T_00091037

Query:           1 CTTTGACATGTACTAAATTATGGT
 25
                   |||||||||||||||||||||*|*
Sbjct:         156 GAAACTGTACATGATTTAATACAAA 132
                                 | |    |-----------------------------------1
                                 | |---------------------------------------5
                                 |-----------------------------------------1

>CsmiRNA-n30o-5p
    Score: 1.0  Deg: 5:140:5:58 T_00091036

Query:           1 CTTTGACATGTACTAAATTATGGT
 25
                   |||||||||||||||||||||*|*
Sbjct:         156 GAAACTGTACATGATTTAATACAAA 132
                                 | |    |-----------------------------------1
                                 | |---------------------------------------5
                                 |-----------------------------------------1
>CsmiRNA-n31p
    Score: 2.2  Deg: 6:183:6:60 T_00085947
```

```
Query:          1 GAGATGGTTAGATTTAAGGACTTT
 25
                  |||||*||||:||||||:||||||*
Sbjct:        195 CTCTAACAATTTAAATTTCTGAAAA 171
                        ||   || | |---------------------------------------2
                        ||   || |-----------------------------------------1
                        ||   ||-------------------------------------------6
                        ||   |--------------------------------------------2
                        ||------------------------------------------------1
                        |-------------------------------------------------1
>CsmiRNA-n31p
    Score: 2.2  Deg: 6:3060:6:267  T_00058209

Query:          1 GAGATGGTTAGATTTAAGGACTTT
 25
                  |||||*||||:||||||:||||||*
Sbjct:       3072 CTCTAACAATTTAAATTTCTGAAAA 3048
                             || | |---------------------------------------2
                             || |-----------------------------------------1
                             ||-------------------------------------------6
                             |--------------------------------------------2

>CsmiRNA-n31p
    Score: 2.2  Deg: 6:1259:6:104  T_00058207

Query:          1 GAGATGGTTAGATTTAAGGACTTT
 25
                  |||||*||||:||||||:||||||*
Sbjct:       1271 CTCTAACAATTTAAATTTCTGAAAA 1247
                             || | |---------------------------------------2
                             || |-----------------------------------------1
                             ||-------------------------------------------6
                             |--------------------------------------------2

>CsmiRNA-n31p
    Score: 2.2  Deg: 6:3031:6:305  T_00058208

Query:          1 GAGATGGTTAGATTTAAGGACTTT
 25
                  |||||*||||:||||||:||||||*
Sbjct:       3043 CTCTAACAATTTAAATTTCTGAAAA 3019
                             || | |---------------------------------------2
                             || |-----------------------------------------1
                             ||-------------------------------------------6
                             |--------------------------------------------2
```

```
>CsmiRNA-n54a.2
    Score: 1.8  Deg: 5:2430:33:75  T_00094820

Query:          1 ACTGCAACCACTTTAGAAACTCAA
 25
                  |||||||||||||||:|||*:|||*
Sbjct:       2432 TGACGTTGGTGAAATTTTTAGGTTT 2408
                    ||    |    |-------------------------------------------1
                    ||    |------------------------------------------------1
                    ||----------------------------------------------------1
                    |-----------------------------------------------------5

>CsmiRNA-n54a.2
    Score: 1.8  Deg: 5:2413:33:76  T_00094821

Query:          1 ACTGCAACCACTTTAGAAACTCAA
 25
                  |||||||||||||||:|||*:|||*
Sbjct:       2415 TGACGTTGGTGAAATTTTTAGGTTT 2391
                    ||    |    |-------------------------------------------1
                    ||    |------------------------------------------------1
                    ||----------------------------------------------------1
                    |-----------------------------------------------------5

>CsmiRNA-n54a.2
    Score: 1.8  Deg: 5:2498:33:76  T_00094823

Query:          1 ACTGCAACCACTTTAGAAACTCAA
 25
                  |||||||||||||||:|||*:|||*
Sbjct:       2500 TGACGTTGGTGAAATTTTTAGGTTT 2476
                    ||    |    |-------------------------------------------1
                    ||    |------------------------------------------------1
                    ||----------------------------------------------------1
                    |-----------------------------------------------------5

>CsmiRNA-n54a.2
    Score: 1.8  Deg: 5:2515:33:75  T_00094822

Query:          1 ACTGCAACCACTTTAGAAACTCAA
 25
                  |||||||||||||||:|||*:|||*
Sbjct:       2517 TGACGTTGGTGAAATTTTTAGGTTT 2493
                    ||    |    |-------------------------------------------1
```

```
                    ||    |-----------------------------------------------1
                    ||--------------------------------------------------1
                    |---------------------------------------------------5
>CsmiRNA-n56b.1
    Score: 2.5  Deg: 6:316:6:162   T_00067103

Query:           1 CTCTTCCTCTCTCTCAAACTG
 22
                   |||||*|||||||||||*||**
Sbjct:         329 GAGAAAGAGAGAGAGTTGGAAG 308
                    |        | |||------------------------------------2
                    |        | ||-------------------------------------6
                    |        | |--------------------------------------3
                    |        |----------------------------------------1
                    |-------------------------------------------------1

>CsmiRNA-n56b.1
    Score: 2.5  Deg: 6:605:6:166   T_00067102

Query:           1 CTCTTCCTCTCTCTCAAACTG
 22
                   |||||*|||||||||||*||**
Sbjct:         618 GAGAAAGAGAGAGAGTTGGAAG 597
                    |        | |||------------------------------------2
                    |        | ||-------------------------------------6
                    |        | |--------------------------------------3
                    |        |----------------------------------------1
                    |-------------------------------------------------1
```
