# Supplementary figures and images for: Genome-Wide Analysis of Alternative Splicing and Non-Coding RNAs Reveal Complicated Transcriptional Regulation in Cannabis sativa L
Source: Int J Mol Sci. 2021 Nov 5;22(21):11989. doi: 10.3390/ijms222111989 (PMC8584933; doi:10.3390/ijms222111989)

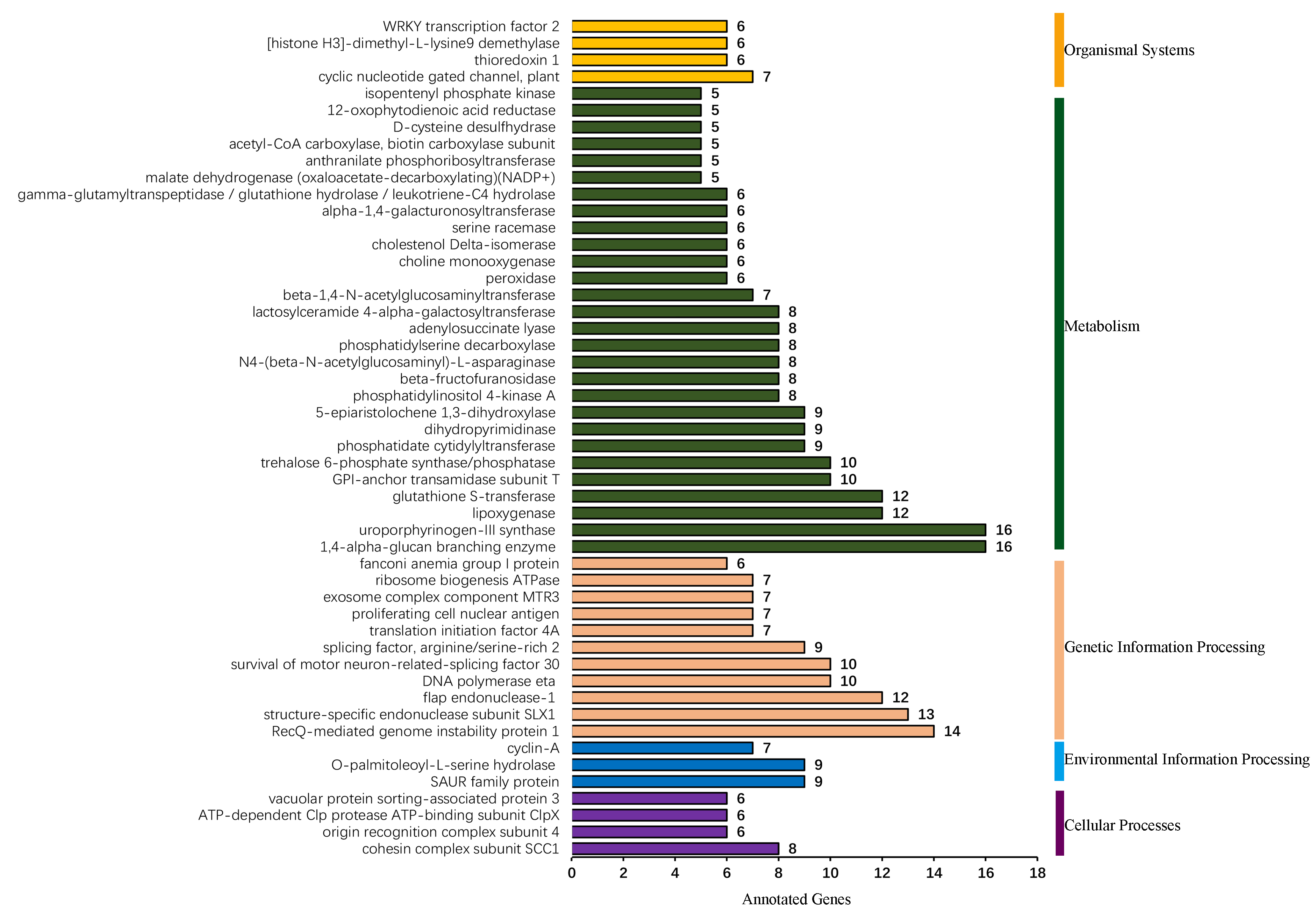

Supplement: Supplementary file 1 [file ijms-22-11989-s001.zip › Figure S2. KEGG enrichment of lncRNA targets.tif]

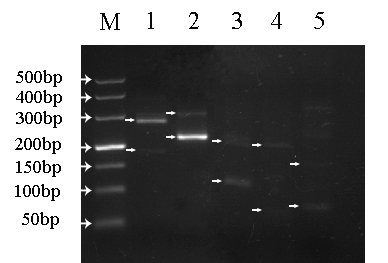

Supplement: Supplementary file 1 [file ijms-22-11989-s001.zip › Figure. S1. AS validation of 5 genes by RT-PCR..tif]
